# The impact of medical staff’s character strengths on job performance in Hangzhou hospitals

**DOI:** 10.3389/fpsyg.2023.1291851

**Published:** 2023-11-23

**Authors:** Xin Zhou, Yinrui Zhang, Yuhang Wang, Haixia Wang, Shuaijun Sun, Xianhong Huang

**Affiliations:** ^1^School of Public Policy and Administration, Xi'an Jiaotong University, Xi’an, China; ^2^First Affiliated Hospital, School of Medicine, Zhejiang University, Hangzhou, Zhejiang, China; ^3^Tianlai Primary School, Hebi City People's Hospital, Hebi, China; ^4^Department of Health Policy and Management, School of Public Health, Hangzhou Normal University, Hangzhou, Zhejiang, China; ^5^Jiangsu Key Laboratory for Pharmacology and Safety Evaluation of Chinese Materia Medica, School of Pharmacy, Nanjing University of Chinese Medicine, Nanjing, Jiangsu, China

**Keywords:** medical staff, character strengths, job performance, career calling, mediating role

## Abstract

**Background:**

Improving job performance has a significant effect on the quality of medical services and ensuring people’s health.

**Purpose:**

This study explores the influence and mechanism of the character strengths and career callings of medical staff as well as the intermediary role of such career calling.

**Methodology:**

A cross-sectional survey was conducted of 414 healthcare staff members in public hospitals in Hangzhou. Descriptive statistics and hierarchical linear regressions were used to analyze the medical staff’s job performance and related factors, and structural equation modeling path analysis was used to explore and validate the influence and mechanism of character advantage and career calling on job performance.

**Results:**

The results show that medical staff character strengths have a positive impact on job performance. Path analysis shows that character strengths indirectly affect job performance, and career calling plays a partial mediating role in character strengths and job performance.

**Conclusion:**

The results show that good personalities promote job performance, and the association is more significant under a high sense of career calling.

## 1 Introduction

Job performance refers to the performances and achievements of professional people in their work (Colquitt, 2001). The job performance of medical staff is an important measure of medical institutions’ ability to provide high-quality medical service, which is significant in their operation and development. Improving medical staff’s job performance helps improve the quality of hospital medical services and the relationship between doctors and patients, which plays a substantial role in the stable and rapid development of hospitals (Wu and Ma, 2018). Job performance is influenced by personal factors, organizational factors, and external environmental factors. Personal factors include the knowledge level, skills, and experience of medical staff. Organizational factors include their working environment and resources. External environmental factors include policies and regulations, field competition, and social demands. These factors affect the development strategy and objectives of medical institutions, in turn affecting the job performance of medical staff. Currently, research on medical staff job performance in domestic academic circles mostly considers the parameters of psychological capital, job competence, and organizational commitment (Meng and Li, 2017; Sun, 2017; Zhu et al., 2019). Our results show that psychological capital, job competence, organizational commitment, innovative behavior, and other factors affect job performance. This research also provides methods and strategies for enterprises and managers to help medical staff improve job performance. Although research on medical staff job performance has made some progress, there remain limitations, such as research being mostly confined to a certain hospital or region, which limits wide applicability. Moreover, researchers have only paid attention to influencing factors without exploring their mechanism. Research on medical staff job performance still lacks cross-cutting approaches with other fields, such as psychology and human resource management. Research results in these fields may provide new ideas and methods for research on medical staff job performance.

With social progress and the development of science and technology, people have higher requirements for the quality and efficiency of medical services, which are closely related to medical staff job performance. Lower job performance will directly lead to a decline in the quality and efficiency of medical services, thus affecting the normal functioning of the overall medical system. One of hospital management’s important goals is good job performance. High-level job performance can not only provide patients with high-quality medical services but also constitute one of the decisive conditions for maintaining hospital competitiveness (She, 2019). Numerous studies show that the job performance of domestic medical staff is at a medium level and there remains room for improvement (Yu et al., 2014; Han, 2018). Therefore, investigating the factors that affect medical staff job performance can provide information for medical policymakers and meaningful insights for improving medical staff performance.

Character strengths are positive qualities expressed through individual thoughts, feelings, and behaviors (Peterson and Seligman, 2004). Some studies have explored the interactions among character strengths, self-efficacy, social support, and depression and psychological well-being and report that character strengths directly and positively relate to mental health (Blanca et al., 2018). Pang and Ruch (2019b) conducted a cross-sectional study to evaluate the relationship between character strengths and job happiness, showing that the character strengths of hope and enthusiasm correlate with happiness. Harzer and Ruch (2014) showed that character strengths relate to work results.

Career calling refers to the individual’s meaningful involvement in a professional role; it regards the needs of others and social interests as an individual’s driving force in pursuing the meaning of life (Dobrow, 2004). Recently, with the rise of positive psychology, studies have shown that work resources correlate positively to career calling (Zhao et al., 2022). One survey found that teachers’ job burnout affects their career calling (Shang et al., 2022). Huang et al. (2022) showed that career calling had a positive moderating effect on the path of “job resources–job satisfaction.” Vianello et al. (2022) showed that employees’ occupational calling is positively related to their job performance. Similarly, Shen and Qian (2022) showed that a positive correlation exists between the career calling and job satisfaction of oncology nurses. However, at present, research on job calling’s mechanism in improving job performance is relatively simple and has not considered character strengths and other factors that improve job performance through job calling.

Healthcare professionals play a crucial role in medical services, and their personality traits may have an impact on their job performance, thus affecting their work outcomes. Therefore, investigating the influence of healthcare professionals’ character strengths on job performance is of great theoretical significance and practical value. By studying the relationship between healthcare professionals’ personality traits and job performance, it can provide a scientific basis for human resource management in the healthcare industry and improve healthcare professionals’ job performance. Although there have been research findings in the field of healthcare professionals’ job performance, issues remain. First, there is a lack of clear research and evidence on the relationship between healthcare professionals’ personality strengths and job performance. Second, there is a lack of research on whether the sense of calling (i.e., the motivation and sense of mission in choosing and remaining in the medical profession) plays an intermediate role in the relationship between character strengths and job performance. Therefore, it is necessary to conduct further research on this relationship.

The significance of this study lies in clarifying the mediating role of career calling, exploring and analyzing the influence of character strengths on sense of calling, and further investigating the mechanism through which career calling enhances job performance. This study aims to provide a scientific basis for the relationship between healthcare professionals’ character strengths and job performance in the healthcare industry in Hangzhou. On one hand, this study further expands the application boundary of self-determination theory. On the other, it explores the influencing factors of career calling, opens the black box regarding the relationship between medical staff career calling and job performance from a new perspective, deepens our understanding of the mechanism mediating career calling and job performance, explicates the promotion mechanism of career calling’s influence on job performance, and posits effective strategies for improving job performance. It provides a theoretical basis for systematically improving medical staff work performance while simultaneously providing new governance concepts and an empirical basis for health policymakers and hospital managers to solve the problem of improving medical staff work performance. This will stimulate the enthusiasm of medical staff, improving the quality of medical services, as well as better meet health needs and ensure the sustainable development of medical and health undertakings.

### 1.1 Theories and assumptions

#### 1.1.1 Character strength’s influence on job performance

Studies have shown that character strengths closely relates to job performance. One study abroad showed that separate dimensions of job performance differ because of the differences in character strengths (Harzer et al., 2021). From the study of character strengths’ influence on job performance, for example, Gander et al. (2020) showed that character strengths positively correlate with job performance. Pang and Ruch (2019a) showed that character strengths play an important role in job performance. Kalyar and Kalyar (2018) showed that a positive relationship between character strengths and the creative work performance of employees. Accordingly, we posit the following Hypothesis 1 (H1): Character strengths positively affect the job performance of medical staff.

#### 1.1.2 The influence of character advantage on career calling

Current research shows that personality advantage can positively impact career calling. Gander et al. (2020) showed that specific roles and character strengths accompany individual performance and work satisfaction. Haslip and Donaldson (2021) showed that character strengths improve performance and well-being. Darewych et al. (2021) showed that character strengths can motivate individuals to flourish in doing their work. Kachel et al. (2021) showed that character strengths can positively contribute to well-being and work-related health. Therefore, we propose Hypothesis 2 (H2): Character strengths have a positive effect on career calling.

#### 1.1.3 The impact of career calling on job performance and its mediating role

Studies have shown that career calling impacts job performance. For example, Ding and Liu (2022) found that career growth, career calling, and inclusive leadership can significantly improve the innovation performance of knowledge workers. Vianello et al. (2022) found that career calling fosters job performance. Laura et al. (2022) found that a sense of calling influences career choice and professional stability and might play a protective role in exhaustion or dissatisfaction at work. Based on the above evidence, we predict Hypothesis 3 (H3): Career calling positively impacts the job performance of medical staff.

**Figure 1:**
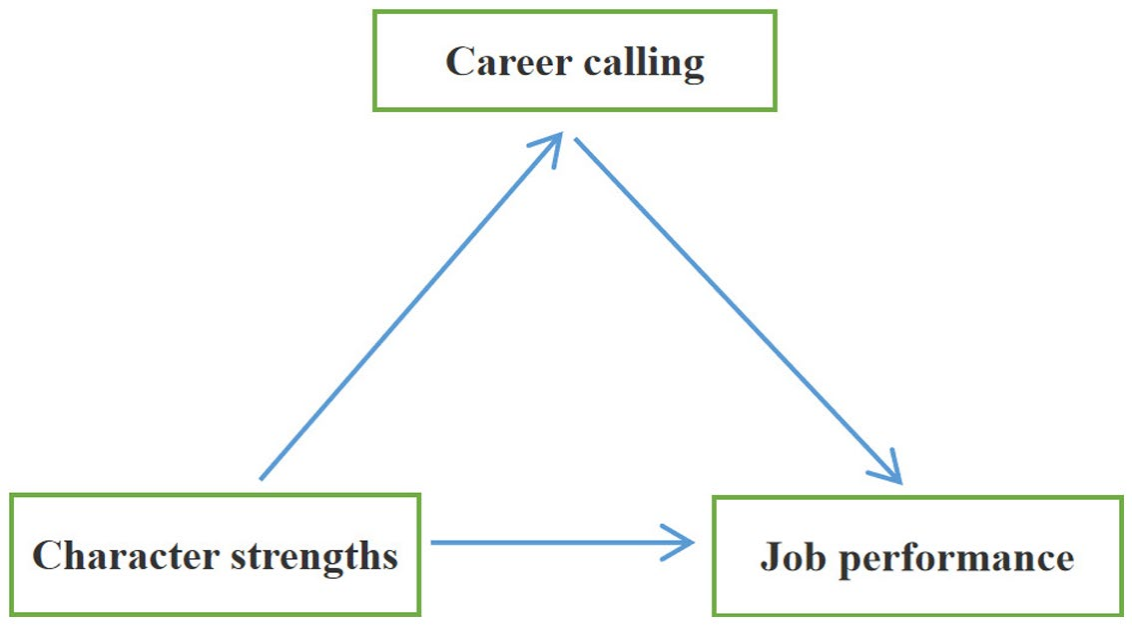
Hypothetical diagram of the relationship among medical staff character strengths, career calling, and job performance.

Studies have shown that there is a close relationship between career calling and job performance. Career development opportunities and staff working conditions impact job performance through career calling, and career calling plays an intermediary role (Wu et al., 2021). Liu et al. (2023) showed that career calling plays a mediating role between spiritual leadership and employee safety performance. Wu et al. (2019) showed that career calling negatively moderates the relationship between role ambiguity and job burnout and positively moderates the relationship between role conflict and job performance. Min et al. (2017) showed that career calling significantly moderates the relationship between perceiving a calling and career adaptability. Therefore, we posit Hypothesis 4 (H4): Career calling plays a mediating role in the path from character strengths to job performance. In brief, there are correlations among character strengths, career calling, and job performance (Figure 1).

## 2 Materials and methods

### 2.1 Participants and procedures

Hangzhou is an economically developed capital city located on the southeast coast of China, with a per capital GDP of about 151,700 yuan in 2021. There are more than 20 tertiary public hospitals, more than 30 secondary public hospitals, and more than 60 community health service centers in Hangzhou. In this study, a total of 32 survey questions were included in the questionnaire, with 12 items related to career calling, four items related to character strengths, and 16 items related to job performance. The Kendel1 sample estimation method was used, whereby the sample size was set to eight times the number of items, and an additional 20% sample size was added to account for potential inaccuracies or missing data in the survey process. Thus, the preliminary sample size was 461, and 414 qualified questionnaires were collected, resulting in an effective rate of 89.8%. We used a stratified random sampling method to investigate the medical staff working in different levels of Hangzhou medical institutions. We selected four tertiary hospitals, two secondary hospitals, and two grass-roots community health service centers from different levels of medical institutions and selected the medical staff from each hospital by convenience sampling. According to the size of hospitals, 414 people were selected from tertiary hospitals, 32 from secondary hospitals, and 14 from grass-roots community health service centers.

The inclusion criteria of study participants are that they be health workers directly involved in providing medical and nursing services for patients, including doctors, nurses, and medical technicians; persons who were working in the institution that day and have worked in the institution for more than half a year; and health workers who agreed to participate in the study. The exclusion criteria are health workers, interns, and trainees from other institutions; health workers who left work or did not agree to participate; and administrative or logistical personnel who are not medical professionals. Before the comprehensive investigation, a preliminary study was conducted to determine the specific implementation and the final version of the questionnaire. The formal investigation was conducted by two researchers and five graduate students with sufficient field investigation experience from July 1 to September 30, 2020. The investigators were trained to use the same standards and methods. During the site investigation, they adopted a one-to-one approach. Before the investigation, they obtained the informed consent of the respondents and gave necessary explanations. After the investigation, they checked the questionnaire to determine whether it met the requirements, numbered the questionnaires uniformly, and entered data twice to ensure accuracy. Table 1 lists the characteristics of the final 414 participants (Table 1).

**Table 1:** Descriptive statistics of medical staffs.

Variable	Category	Quantity (person)	Percentage (%)
Gender	Male	165	39.9
	Female	249	60.1
Age	29 years old and below	140	33.8
	30–39	202	48.8
	40–49	59	14.3
	50 years old and above	13	3.1
Professional post	Clinician	232	56.0
	Nurse	103	24.9
	Medical and technical staff	79	19.1
Academic degree	College degree or below	4	1.0
	Undergraduate course	234	56.5
	Master	140	33.8
	PhD	36	8.7
Working time	5 years and below	173	41.8
	6–10 years	111	26.8
	11–15 years	74	17.9
	16–20 years	33	8.0
	21 years and above	23	5.6
Professional title	No professional title	74	17.9
	Primary	122	29.5
	Middle	157	37.9
	Deputy senior	48	11.6
	Positive advanced	13	3.1
Authorized strength	Have a system	277	66.9
	Contract employment	137	33.1
Position	No position	328	79.2
	Treatment/Responsibility Team Leader	74	17.9
	Department leader	12	2.9
Departments	Internal medicine	80	19.3
	Surgery department	112	27.1
	Emergency department	19	4.6
	Pediatrics	23	5.6
	Gynecology and obstetrics	31	7.5
	Intensive care unit	11	2.7
	Medical department (inspection, radiation, etc.)	73	17.6
	Others	65	15.7
Hospital level	Tertiary hospital	373	90.1
	Secondary hospital	29	7.0
	community health service center	12	2.9

### 2.2 Measures

#### 2.2.1 General information

There are 10 items in the census form, including gender (male or female), age (29 years old and below, 30–39, 40–49, 50, and above), professional position (clinician, nurse, medical technician), educational background (junior college and below, undergraduate, master’s and doctoral students), and working time (5 years and below, 6–10 years, 11–15 years, 16–20 years, 21 years and above), professional title (no professional title, primary, middle, deputy senior, positive advanced), establishment (including establishment, contract employment), position (no position, treatment/responsibility team leader, department leader), department (internal medicine, surgery, emergency department, pediatrics, obstetrics and gynecology, intensive care unit, medical technology department, others), and hospital level (tertiary hospital, secondary hospital, community health service center).

#### 2.2.2 Career calling scale

The Career Calling Scale CQ12 compiled by Dobrow and Tosti-Kharas (2011) was selected for career calling measurement. The scale has one dimension and 12 items in total, all scored on a 5-point Likert scale, where 1 stands for *completely disagree* and 5 stands for *completely agree*. The scale has been widely used in the study of occupational calling, with good reliability and validity being reported. In this study, the Cronbach’s α coefficient of the occupational call scale is 0.955, which shows good reliability.

#### 2.2.3 Character strengths scale

The character strengths Scale of a medical staff member are measures based on the Character Strengths Scale for Medical Staff compiled by Lu (2010), which mainly includes three aspects: affinity, vitality, and willpower. Items are scored on a 5-point Likert scale, where 1 stands for *completely disagree* and 5 stands for *completely agree*. In this study, the Cronbach’s α coefficient of character strengths scale is 0.863, which has good reliability.

#### 2.2.4 Job performance scale

The Job Performance Scale for rural public health service personnel compiled by Fu (2018) was revised with reference to the relevant state documents regarding the performance appraisal of public hospitals and medical personnel. The scale includes 16 items along five dimensions: medical service performance, interpersonal promotion, work dedication, personal growth, and professional identity. All items are scored on a 5-point Likert scale, where 1 stands for *completely disagree* and 5 stands for *completely agree*. In this study, the overall Cronbach’s α coefficient of the job performance scale is 0.953, and the Cronbach’s α coefficients of medical service performance, interpersonal promotion, job dedication, personal growth, and professional identity are 0.931, 0.939, 0.872, 0.941, and 0.884, respectively, indicating good reliability. The Career Calling Scale, Character Strengths Scale, and Job Performance Scale show good validity.

### 2.3 Statistical analysis

SPSS 25.0 was used for statistical analysis. We used descriptive statistics such as frequency and composition ratio to analyze the data. Internal consistency (Cronbach’s α coefficient) and composite reliability (CR) were used to evaluate the reliability of the questionnaire, and the validity of the measurement was evaluated using content and aggregation validity. The differences among medical staff scores in character strengths, occupational calling, and job performance were analyzed by t-test and one-way ANOVA. The main factors affecting medical staff job performance and the promotion effect of career calling on job performance were analyzed by hierarchical linear regression. Using a forward stepwise multiple regression analysis with job performance as the dependent variable, all variables that might affect job performance were added to the model to manage any possible confusion. The structural equation model (SEM) was constructed with AMOS 28.0, and the influence mechanism of character strengths and career calling on job performance (directly or indirectly) was explored and their influences calculated. The main paths involved in the model are character strengths → career call → job performance. Career call judges the model’s adaptation through a chi-square fitness test and other related tests and modifies the basic model in combination with suggestions for model modification so as to achieve the best adaptation between the modified model and sample data. The bootstrap method was used to verify the intermediary role of career call, and Ping’s (1995) two-step method was used to analyze its regulatory effect. We judged the goodness of fit of the hypothetical model and modified it with the correction index, so that the modified model fit the sample data more closely.

## 3 Results

### 3.1 Descriptive statistics and correlation analysis of medical staff

There is a positive correlation between character strengths and all dimensions of job performance, and the correlation coefficient is *r* = 0.526–0.608 (all *p* < 0.01), among which character strengths have the highest correlation with job dedication (*r* = 0.608) and the lowest correlation with professional identity (*r* = 0.526). There is a positive correlation between occupational calling and job performance, and the correlation coefficient is in the range of *r* = 0.476–0.693 (all *p* < 0.01). Among them, occupational calling has the highest correlation with professional identity (*r* = 0.693) and the lowest correlation with medical service performance (*r* = 0.476). Table 2 presents the situation of medical staff job performance scores; their descriptive statistics are shown in Table 1, and the correlation analysis is shown in Table 3.

**Table 2:** Present situation of job performance scores of medical staffs (*n* = 414).

Job performance dimension	Mean standard deviation (minutes)
Medical service performance	
E1	4.33 ± 0.578
E2	4.29 ± 0.56
E3	4.28 ± 0.608
E4	4.32 ± 0.615
Interpersonal promotion	
E5	4.37 ± 0.561
E6	4.36 ± 0.576
E7	4.38 ± 0.552
E8	4.38 ± 0.561
Work contribution	
E9	4.11 ± 0.711
E10	4.14 ± 0.738
Personal growth	
E11	4.32 ± 0.653
E12	4.33 ± 0.656
E13	4.36 ± 0.649
Professional identity	
E14	4.3 ± 0.712
E15	4.27 ± 0.682
E16	4.1 ± 0.948

**Table 3:** Analysis of the scores and correlation of medical staff’s character strength, career calling and work performance.

	Job Performance	Medical service performance	Interpersonal facilitation	Work dedication	Personal growth	Career identification
Character strength	0.661^**^	0.535^**^	0.543^**^	0.608^**^	0.579^**^	0.526^**^
Career calling	0.687^**^	0.476^**^	0.530^**^	0.562^**^	0.617^**^	0.693^**^

^*^Indicates a statistically significant difference (*p < 0.05, **p < 0.01).

### 3.2 Analysis of job performance differences of medical staff with different demographic characteristics

There is no statistical significance in the influence of gender, professional post, working time, professional title, or position on the job performance of medical staff (*p* > 0.05). There are statistical differences in their job performance regarding age, academic degree, authorized strength, departments, and hospital level (*p* < 0.05). The medical staff whose department was obstetrics and gynecology scored the highest, and those in the intensive care unit scored the lowest, with a statistically significant difference (*p* < 0.005). The medical staff in tertiary hospitals scored the highest, while the medical staff in community health service centers scored the lowest, and the difference was statistically significant (*p* < 0.005). See Table 4 for details.

**Table 4:** Analysis of job performance differences of medical staff with different demographic characteristics.

Variance		Quantity (person)	Mean standard deviation (minutes)	*F* ratio	*P* ratio
Age	29 years and below	140	4.19 ± 0.47	3.126	0.033
	30–39 years	202	4.34 ± 0.54		
	40–49 years	59	4.37 ± 0.41		
	50 years and above	13	4.32 ± 0.50		
Academic degree	College degree or below	4	4.13 ± 0.60	3.063	0.028
	Undergraduate course	234	4.25 ± 0.5		
	Master	140	4.31 ± 0.49		
	PhD	36	4.51 ± 0.49		
Authorized strength	Have a system	277	4.33 ± 0.51	4.833	0.028
	Contract employment	137	4.21 ± 0.49		
Department	Internal medicine	80	4.16 ± 0.55	3.267	0.002
	Surgery department	112	4.37 ± 0.50		
	Emergency department	19	4.10 ± 0.52		
	Pediatrics	23	4.32 ± 0.50		
	Gynecology and obstetrics	31	4.53 ± 0.46		
	Intensive care unit	11	4.11 ± 0.52		
	Medical department (inspection, radiation, etc.)	73	4.35 ± 0.46		
	Others	65	4.21 ± 0.44		
Hospital grade	Tertiary hospitals	373	4.33 ± 0.47	12.472	0.000
	Secondary hospital	29	4.09 ± 0.63		
	Community service center	12	3.69 ± 0.58		

Compared with those aged 29 and below, doctoral students, unemployed, emergency departments and community health service centers.

### 3.3 Regression analysis of job performance

Job performance is used as the dependent variable, while character strength and career calling are used as independent variables. Taking into account the possible interference of demographic variables in the model, we include them as control variables. Model 1 examines the impact of control variables on the dependent variable, Model 2 adds the influence of character strength on the dependent variable while controlling for other variables, and Model 3 includes the influence of demographic variables, character strength, and career calling on the dependent variable.

The results show that Model 1 has an *R*-square of 0.147, indicating that the control variables explain 14.7% of the variance in the dependent variable. The *F*-value is 2.283, and the *value of p* is less than 0.001. Model 2 has an *R*-square of 0.509, indicating that the inclusion of character strength after controlling for other variables explains 50.9% of the variance in the dependent variable. The *F*-value is 13.215, and the *value of p* is less than 0.001. Model 3 has an *R*-square of 0.614, indicating that the inclusion of demographic variables, character strength, and career calling explains 61.4% of the variance in the dependent variable. The *F*-value is 19.602, and the *p* is less than 0.001. These findings suggest that the models we have constructed are meaningful. After controlling for the interference of educational background and department, character strength has a positive impact on job performance (*β* = 0.351, *p* < 0.001), and career calling also has a positive impact on job performance (*β* = 0.316, *p* < 0.001).

### 3.4 Model construction

**Figure 2:**
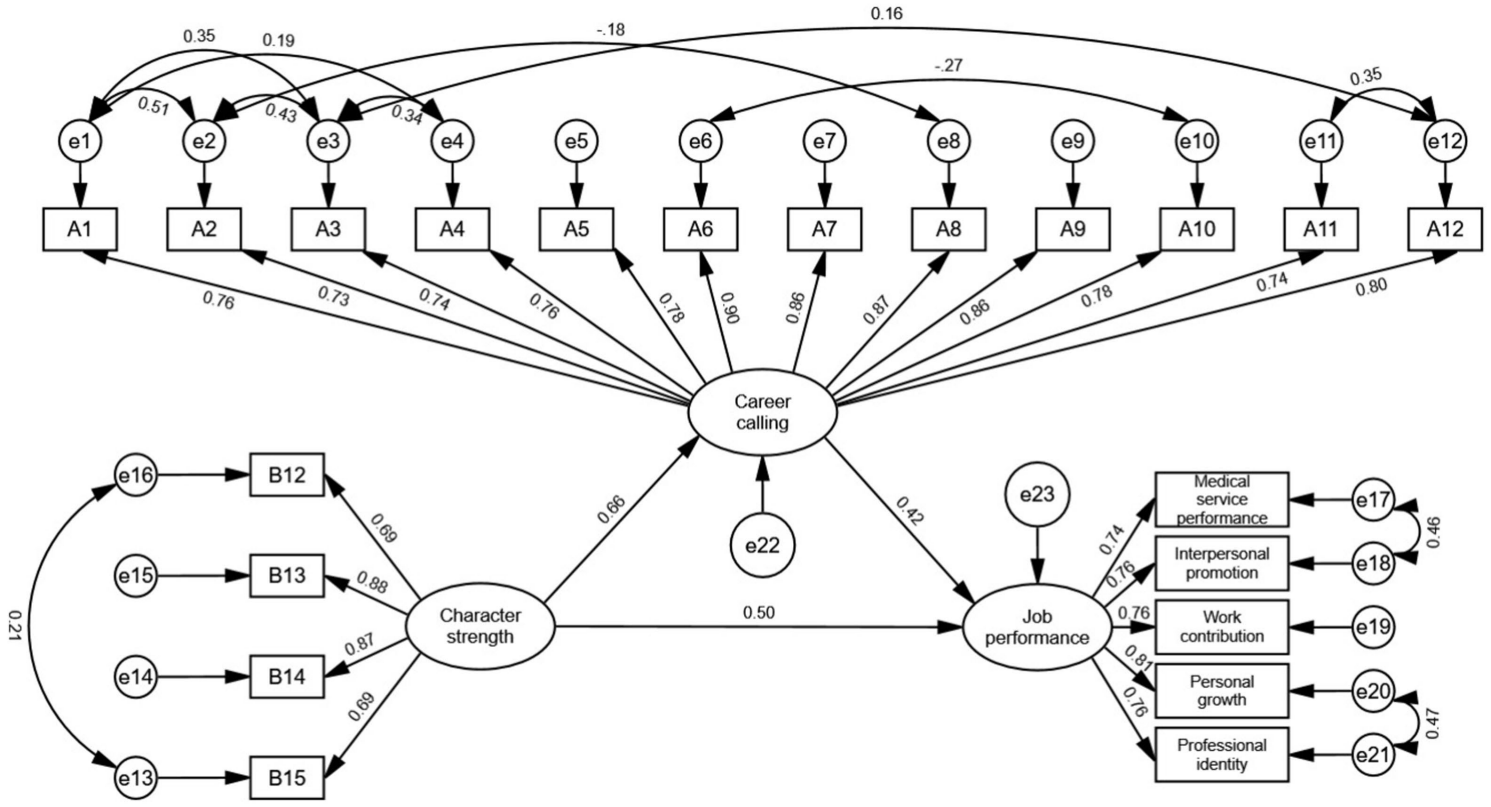
Modified structural equation model diagram.

The present study used structural equation modeling to empirically analyze the mechanism of the effects of character strength and career calling on job performance among healthcare professionals. As the fit indices of the initial model did not reach the desired threshold, the model was expanded based on the magnitude of the modification indices. Specifically, additional paths were added between the residual terms of e1 and e2, e17 and e18, e3 and e4, e11 and e12, e2 and e3, e1 and e3, e20 and e21, e6 and e10, e2 and e8, e3 and e12, e1 and e4, and e13 and e16. The final revised model is depicted in Figure 2, and the specific fit results are presented in Table 5. According to the model fit test results in Table 5, the revised model exhibited a CMIN/*df* (chi-square–degrees-of-freedom ratio) of 3.607, which falls within the range of 3–5, indicating an acceptable fit. The RMSEA (root mean square error of approximation) was 0.079, indicating a good fit (< 0.08). Additionally, the IFI, TLI, and CFI indices all exceeded 0.9, indicating excellent fit.

**Table 5:** Evaluation index of structural equation model.

Indicator	Evaluation criterion	Initial model	B-S modified model
CMIN/DF	3–5	6.465	3.607
RMSEA	<0.08	0.115	0.079
IFI	>0.9	0.864	0.939
TLI	>0.9	0.846	0.926
CFI	>0.9	0.863	0.939

CMIN/DF, Chi-Square/Degrees of Freedom ratio; RMSEA, Root Mean Square Error of Approximation; IFI, Incremental Fit Index; TLI, Tucker-Lewis Index; CFI, Comparative Fit Index.

### 3.5 Path analysis results of the fitting model

According to the analysis results in Table 2, it can be observed that in the path hypothesis testing of this study, character strength significantly and positively predicted career calling (*β* = 0.656, *p <* 0.001). Additionally, character strength and career calling both significantly and positively predicted job performance (*β* = 0.499, *β* = 0.422, *p <* 0.001), as shown in Table 6.

**Table 6:** Path coefficient among factors of medical staff’s job performance.

Variable relation	Non-standardized path estimation value	Standardized path estimation value	S.E.	*t*	*P*
Career calling←Character strength	0.801	0.656	0.075	10.687	***
Job performance←Career calling	0.265	0.422	0.036	7.374	***
Job performance←Character strength	0.382	0.499	0.048	7.934	***
Medical service performance←Job performance	1	0.739			
Interpersonal facilitation←Job performance	0.991	0.761	0.048	20.440	***
Work contribution←Job performance	1.305	0.760	0.089	14.667	***
Personal growth←Job performance	1.257	0.809	0.081	15.468	***
Occupational identity←Job performance	1.354	0.756	0.094	14.401	***

^*^Indicates a statistically significant difference (***p < 0.001).

### 3.6 A bootstrap test of career calling

The results show that the overall effect value, direct effect value, and indirect effect value of the character strength of healthcare professionals on job performance are 0.595, 0.382, and 0.212, respectively. The 95% confidence intervals for all three values do not include 0. Table 7 shows the presence of partial mediation effects for career calling.

**Table 7:** Bootstrap test PF intermediary effect (standardized coefficient).

Route	Type of interaction	SE	Effect value	Bias-corrected 95%Confidence Level		Percenti1e 95%Confidence Level	
				CL lower limit	CL higher limit	CL lower limit	CL higher limit
Character strengths→Job performance	Overall effect	0.067	0.595	0.477	0.739	0.474	0.735
	Direct effect	0.071	0.382	0.256	0.533	0.252	0.528
	Indirect effect	0.039	0.212	0.146	0.299	0.143	0.295
Career calling→Job performance	Overall effect	0.050	0.265	0.176	0.369	0.179	0.372
	Direct effect	0.050	0.265	0.176	0.369	0.179	0.372

## 4 Discussion

This project takes the mediating effect of career calling as the entry point to test career calling’s mediation in the relationship between character strengths and job performance. The results show that character strengths and career calling positively impact job performance. In addition, the mediating role of career calling shows that the richness of character strengths increases career calling, thus promoting job performance.

The above is based on empirical research that verifies the relationship between career calling, character strengths, and job performance. The main theoretical findings and innovations are as follows. First, the research is based on self-determination theory and focuses on the mediating effect of career calling, revealing its role between character strengths and job performance. It effectively validates the research achievements of positive psychology in healthcare professionals. Second, the research results demonstrate the positive impact of character strengths and career calling on job performance, further expanding the research outcomes in this field. Third, the mediating role of career calling suggests that the abundant presence of character strengths enhances career calling, thereby promoting job performance in healthcare professionals. This provides important insights and suggestions for future research on creative performance in human resource management.

### 4.1 Character strengths and job performance

The results of this study show that medical staff character strengths positively impact job performance, which is consistent with Huang’s (2021) and Sun et al.’s (2016) results. Therefore, knowing medical staff’s character strengths can help medical institutions better manage and train staff and improve their work performance. The results show that medical staff with a calm personality are diligent, meticulous, and conscientious in their work, calm in analysis when encountering problems, and able to better complete medical services. Helpful medical staff members are generally enthusiastic and cheerful, like to manage colleagues, and are willing to answer patients patiently, which plays a positive role in improving interpersonal promotion and willingness toward work contributions. Optimistic medical staff members accept new elements quickly, have a strong ability to adapt to the environment, and are more likely to actualize their potential. Facing work challenges and pressures, they will mature quickly and have a stronger professional sense. Medical staff members with strong interpersonal skills and communication skills are more likely to gain the trust and support of patients and their families, thus improving their work performance. Medical staff can thus explore and cultivate their own character strengths, improve their work performance, and provide better medical services for patients. Simultaneously, organizations can improve the quality and efficiency of medical services by effectively identifying and cultivating character strengths among medical staff.

### 4.2 Career calling and job performance

The results of this study show that career calling has a positive effect on job performance, which is consistent with the research results of scholars such as Wu et al. (2021). Career calling is a type of work passion and driving force, which can give individuals a sense of meaning in their work (Tan, 2018). Medical staff with a high level of professional calling have strong work passion and motivation, which improves their contribution to medical work. Second, a higher level of occupational summoning promotes the maintenance of positive work attitude and vitality, which enables staff to maintain a positive state at work and positively impacts the management of interpersonal relationships among colleagues and the improvement of medical service performance (Gao, 2019). In addition, medical staff with a high sense of professional calling sincerely regard their work as a way to realize their self-worth, enjoy career development and personal growth, and improve their interest and excitement in their work, thus further improving their performance. Therefore, to improve work performance when recruiting medical staff, it is possible to learn about their career calling through a questionnaire, giving priority to staff with a higher sense of career calling. In addition, hospitals must pay more attention to the cultivation of the medical staff’s professional calling level, work beliefs, and sense of mission so that medical staff can develop a strong sense of professional sacredness and honor in medical work, further improving their work performance.

### 4.3 Character strengths and career calling

Our research shows that character strengths positively influence career calling. This is consistent with the longstanding results of Huber et al. (2020). Medical staff with high scores in character strengths are more likely to have a strong sense of professional calling. This may be because character strengths lead to better adaptation to the working environment, enhanced working ability and self-confidence, and thus greater willingness for career devotion and enjoyment of accomplishment and satisfaction. Character strengths can enhance the sense of professional calling by improving work attitude and professional identity (Huber et al., 2021). This shows that character strengths can not only influence the individual’s internal psychological state but also positively impact external behaviors and attitudes. In summary, character strengths positively impact career calling, and this research achievement has been verified by many studies. Character strengths can influence career calling by enhancing individuals’ work ability and self-confidence, improving work attitude and professional identity. Therefore, hospitals and enterprise managers should pay attention to the cultivation of the character strengths of medical staff, which will improve their professional calling and work performance.

### 4.4 The mediating effect of career calling between character strengths and job performance

The results of the structural equation model show that career calling plays a partial mediating role in the path from character strengths to job performance, which is consistent with the research results of Fredrickson (2001), Duan and Wang (2019), and Zhang et al. (2023). Personality strengths play an important role in mediating job performance, and taking advantage of these strengths improves job performance. Character strengths enhance people’s belonging, expectations, goals, values, emotions, and self-control; they foster the emergence of career calling, thus promoting creative thinking and performance. Medical personnel should strengthen their own professional identity, enhance motivation, and self-efficacy; cultivate a positive character to actively manage work difficulties; and actively promote their career development to improve work performance.

### 4.5 Theoretical significance

This research has made several contributions to current knowledge. First, our research further explored the mechanism of character strengths’ influence on job performance. Second, it did the same regarding the influencing factors of occupational calling, illuminated the relationship between the occupational calling and job performance of medical staff using a new perspective, and deepened our understanding of the mechanism between the two. Third, our results expand research on the mechanism of occupational calling. Fourth, this study confirmed that job performance is positively correlated with character strengths.

### 4.6 Practical implications

This study showed that character strengths, occupational calling, and job performance are positively correlated, which has significance for improving medical staff job performance and promoting hospital management efficiency. In addition, the higher the character strengths score, the stronger the calling. Therefore, the hospital should pay attention to training related to the character strengths of medical staff. We also found that academic qualifications have a significant impact on job performance, such that the higher the academic qualification is, the higher the job performance level. Therefore, hospitals should not only provide employees with simple jobs but also provide medical staff with long-term opportunities for career development and academic upgrading. This will improve the satisfaction and loyalty of medical staff and patients. The results show that career calling mediates character strengths and job performance. Standardizing the model’s influence coefficient shows that character strengths and career calling positively affect job performance. This study enhanced the understanding of career calling’s promotion mechanism regarding job performance. Based on this, strategies for effectively improving job performance can be posited. A theoretical basis exists for systematically improving medical staff work performance, providing new governance ideas and an empirical basis for health policymakers and hospital managers to solve the problem of improving work performance. Improving the level and quality of medical services will better meet growing health needs. It is important to promote the professional development of medical personnel and the development and innovation of the medical cause.

### 4.7 Limitations and future research

This study has some limitations. First, this is a cross-sectional study, which can only determine correlation and not causality. Therefore, we need to design causality-focused longitudinal studies such as crossover studies or utilize research designs involving two time points. Second, the sample is not sufficiently representative. This study was conducted in Hangzhou, which is a relatively developed city. Therefore, the status of character strengths and career calling in underdeveloped areas will likely differ, and our results may not fully extend to other areas. Future research should expand the geographical scope and make the results more representative. Third, this study focuses on overall job performance without considering more detailed outcome variables. Fourth, the questionnaire survey variables are all self-reported by medical staff, which has certain subjective elements, affecting their authenticity and objectivity. It is inevitable that there is a specific common method deviation, which may not reflect the real feelings and opinions of medical staff. According to recent research, a high level of innovative personality traits is positively correlated with an increase in academic burnout (Khosravi et al., 2020; Khosravi, 2021), especially when personal achievement is considered a strong predictor of healthcare professionals’ performance. Therefore, in the future, incorporating personality traits into research will help provide a more accurate interpretation of study results. Finally, in future research, it is important to incorporate methodological advancements such as employing more experimental designs, increasing sample diversity, utilizing objective measurement tools, and conducting long-term longitudinal studies. These methodological improvements will contribute to enhancing the reliability and generalizability of the research findings, as well as providing more in-depth insights for practical applications.

## 5 Conclusion

This study shows that the Hangzhou health professionals’ overall job performance is middling, and both character strengths and occupational calling impact job performance. Moreover, staff character strengths positively impact job performance. Path analysis shows that character strengths and career calling directly affect job performance. Career calling mediates the relationship between personality advantage and job performance. These results demonstrate that a good personality promotes work performance, which is more significant under the regulation of a high sense of vocational calling. Therefore, medical and hospital managers should find ways to stimulate a sense of professional identity, promote the emergence of a sense of professional calling, and thus enhance creative thinking and performance. In addition, medical staff require a balance between work and life, awareness of emotional needs, and reasonable work demands. To relieve pressure and reduce conflicts, managers may try to improve career calling by cultivating a sense of professional mission, thus improving work performance.

## Data availability statement

The original contributions presented in the study are included in the article/Supplementary material, further inquiries can be directed to the corresponding author.

## Ethics statement

The study was approved by the institutional review board of Hangzhou Normal University. All study participants provided informed consent, and the study was performed in accordance with the ethical standards as laid down in the 1964 Declaration of Helsinki and its later amendments. The studies were conducted in accordance with the local legislation and institutional requirements. The participants provided their written informed consent to participate in this study.

## Author contributions

XZ: Writing – original draft. YZ: Data curation, Writing – review & editing. YW: Writing – review & editing. HW: Data curation, Writing – review & editing. SS: Formal analysis, Writing – review & editing. XH: Conceptualization, Funding acquisition, Writing – review & editing.
