# Supplementary material for: The impact of medical staff’s character strengths on job performance in Hangzhou hospitals
Source: Front Psychol. 2023 Nov 23;14:1291851. doi: 10.3389/fpsyg.2023.1291851 (PMC10701392; doi:10.3389/fpsyg.2023.1291851)
Supplement: Supplementary file 1 [file Data_Sheet_1.ZIP › statistical analysis process/AMOS analysis model outputresults/e17║═e18.AmosOutput]

e17和e18.amw


# C:\Users\zhx08\Desktop\2023.8.2再次核对修改\统计分析过程2023.8.2\8.7AMOS分析模型输出结果\e17和e18.amw

## Analysis Summary

## Date and Time

Date: 2023年8月6日

Time: 22:44:06

## Title

e17和e18: 2023年8月6日 22:44

## Groups

## Group number 1 (Group number 1)

## Notes for Group (Group number 1)

The model is recursive.

Sample size = 414

## Variable Summary (Group number 1)

## Your model contains the following variables (Group number 1)

Observed, endogenous variables

A1

A2

A3

A4

A5

A6

A7

A8

A9

A10

A11

A12

B15

B14

B13

B12

医疗服务绩效

人际促进

工作贡献

个人成长

职业认同

Unobserved, endogenous variables

职业召唤

工作绩效

Unobserved, exogenous variables

e1

e2

e3

e4

e5

e6

e7

e8

e9

e10

e11

e12

性格优势

e13

e14

e15

e16

e17

e18

e19

e20

e21

e22

e23

## Variable counts (Group number 1)

|  |  |
| --- | --- |
| Number of variables in your model: | 47 |
| Number of observed variables: | 21 |
| Number of unobserved variables: | 26 |
| Number of exogenous variables: | 24 |
| Number of endogenous variables: | 23 |

## Parameter Summary (Group number 1)

|  | Weights | Covariances | Variances | Means | Intercepts | Total |
| --- | --- | --- | --- | --- | --- | --- |
| Fixed | 26 | 0 | 0 | 0 | 0 | 26 |
| Labeled | 0 | 0 | 0 | 0 | 0 | 0 |
| Unlabeled | 21 | 3 | 24 | 0 | 0 | 48 |
| Total | 47 | 3 | 24 | 0 | 0 | 74 |

## Models

## Default model (Default model)

## Notes for Model (Default model)

## Computation of degrees of freedom (Default model)

|  |  |
| --- | --- |
| Number of distinct sample moments: | 231 |
| Number of distinct parameters to be estimated: | 48 |
| Degrees of freedom (231 - 48): | 183 |

## Result (Default model)

Minimum was achieved

Chi-square = 917.418

Degrees of freedom = 183

Probability level = .000

## Group number 1 (Group number 1 - Default model)

## Estimates (Group number 1 - Default model)

## Scalar Estimates (Group number 1 - Default model)

## Maximum Likelihood Estimates

## Regression Weights: (Group number 1 - Default model)

|  |  |  | Estimate | S.E. | C.R. | P | Label |
| --- | --- | --- | --- | --- | --- | --- | --- |
| 职业召唤 | <--- | 性格优势 | .795 | .073 | 10.946 | \*\*\* |  |
| 工作绩效 | <--- | 职业召唤 | .281 | .034 | 8.254 | \*\*\* |  |
| 工作绩效 | <--- | 性格优势 | .290 | .041 | 7.038 | \*\*\* |  |
| A1 | <--- | 职业召唤 | 1.000 |  |
| A2 | <--- | 职业召唤 | .945 | .042 | 22.769 | \*\*\* |  |
| A3 | <--- | 职业召唤 | 1.037 | .064 | 16.321 | \*\*\* |  |
| A4 | <--- | 职业召唤 | .950 | .058 | 16.496 | \*\*\* |  |
| A5 | <--- | 职业召唤 | .943 | .056 | 16.866 | \*\*\* |  |
| A6 | <--- | 职业召唤 | 1.120 | .056 | 20.087 | \*\*\* |  |
| A7 | <--- | 职业召唤 | 1.028 | .054 | 18.943 | \*\*\* |  |
| A8 | <--- | 职业召唤 | .997 | .052 | 19.209 | \*\*\* |  |
| A9 | <--- | 职业召唤 | 1.008 | .053 | 18.956 | \*\*\* |  |
| A10 | <--- | 职业召唤 | .878 | .053 | 16.431 | \*\*\* |  |
| A11 | <--- | 职业召唤 | 1.114 | .069 | 16.260 | \*\*\* |  |
| A12 | <--- | 职业召唤 | 1.170 | .065 | 17.897 | \*\*\* |  |
| B15 | <--- | 性格优势 | 1.000 |  |
| B14 | <--- | 性格优势 | 1.011 | .062 | 16.346 | \*\*\* |  |
| B13 | <--- | 性格优势 | .976 | .059 | 16.493 | \*\*\* |  |
| B12 | <--- | 性格优势 | .937 | .069 | 13.556 | \*\*\* |  |
| 医疗服务绩效 | <--- | 工作绩效 | 1.000 |  |
| 人际促进 | <--- | 工作绩效 | .988 | .050 | 19.761 | \*\*\* |  |
| 工作贡献 | <--- | 工作绩效 | 1.303 | .097 | 13.423 | \*\*\* |  |
| 个人成长 | <--- | 工作绩效 | 1.456 | .090 | 16.193 | \*\*\* |  |
| 职业认同 | <--- | 工作绩效 | 1.616 | .103 | 15.713 | \*\*\* |  |

## Standardized Regression Weights: (Group number 1 - Default model)

|  |  |  | Estimate |
| --- | --- | --- | --- |
| 职业召唤 | <--- | 性格优势 | .657 |
| 工作绩效 | <--- | 职业召唤 | .484 |
| 工作绩效 | <--- | 性格优势 | .412 |
| A1 | <--- | 职业召唤 | .761 |
| A2 | <--- | 职业召唤 | .736 |
| A3 | <--- | 职业召唤 | .755 |
| A4 | <--- | 职业召唤 | .762 |
| A5 | <--- | 职业召唤 | .777 |
| A6 | <--- | 职业召唤 | .896 |
| A7 | <--- | 职业召唤 | .855 |
| A8 | <--- | 职业召唤 | .864 |
| A9 | <--- | 职业召唤 | .855 |
| A10 | <--- | 职业召唤 | .760 |
| A11 | <--- | 职业召唤 | .753 |
| A12 | <--- | 职业召唤 | .816 |
| B15 | <--- | 性格优势 | .709 |
| B14 | <--- | 性格优势 | .866 |
| B13 | <--- | 性格优势 | .876 |
| B12 | <--- | 性格优势 | .708 |
| 医疗服务绩效 | <--- | 工作绩效 | .695 |
| 人际促进 | <--- | 工作绩效 | .713 |
| 工作贡献 | <--- | 工作绩效 | .713 |
| 个人成长 | <--- | 工作绩效 | .881 |
| 职业认同 | <--- | 工作绩效 | .849 |

## Covariances: (Group number 1 - Default model)

|  |  |  | Estimate | S.E. | C.R. | P | Label |
| --- | --- | --- | --- | --- | --- | --- | --- |
| e1 | <--> | e2 | .158 | .018 | 8.718 | \*\*\* |  |
| e17 | <--> | e18 | .075 | .009 | 8.416 | \*\*\* |  |
| e3 | <--> | e4 | .118 | .017 | 6.914 | \*\*\* |  |

## Correlations: (Group number 1 - Default model)

|  |  |  | Estimate |
| --- | --- | --- | --- |
| e1 | <--> | e2 | .517 |
| e17 | <--> | e18 | .534 |
| e3 | <--> | e4 | .394 |

## Variances: (Group number 1 - Default model)

|  |  |  | Estimate | S.E. | C.R. | P | Label |
| --- | --- | --- | --- | --- | --- | --- | --- |
| 性格优势 |  |  | .282 | .035 | 7.995 | \*\*\* |  |
| e22 |  |  | .235 | .027 | 8.584 | \*\*\* |  |
| e23 |  |  | .046 | .007 | 6.944 | \*\*\* |  |
| e1 |  |  | .300 | .022 | 13.512 | \*\*\* |  |
| e2 |  |  | .313 | .023 | 13.629 | \*\*\* |  |
| e3 |  |  | .334 | .025 | 13.531 | \*\*\* |  |
| e4 |  |  | .269 | .020 | 13.497 | \*\*\* |  |
| e5 |  |  | .242 | .018 | 13.437 | \*\*\* |  |
| e6 |  |  | .128 | .011 | 11.865 | \*\*\* |  |
| e7 |  |  | .161 | .013 | 12.702 | \*\*\* |  |
| e8 |  |  | .139 | .011 | 12.551 | \*\*\* |  |
| e9 |  |  | .154 | .012 | 12.695 | \*\*\* |  |
| e10 |  |  | .234 | .017 | 13.533 | \*\*\* |  |
| e11 |  |  | .392 | .029 | 13.567 | \*\*\* |  |
| e12 |  |  | .284 | .022 | 13.146 | \*\*\* |  |
| e13 |  |  | .278 | .022 | 12.783 | \*\*\* |  |
| e14 |  |  | .097 | .010 | 9.471 | \*\*\* |  |
| e15 |  |  | .082 | .009 | 9.012 | \*\*\* |  |
| e16 |  |  | .246 | .019 | 12.793 | \*\*\* |  |
| e17 |  |  | .149 | .012 | 12.971 | \*\*\* |  |
| e18 |  |  | .131 | .010 | 12.829 | \*\*\* |  |
| e19 |  |  | .228 | .018 | 12.870 | \*\*\* |  |
| e20 |  |  | .085 | .009 | 9.210 | \*\*\* |  |
| e21 |  |  | .141 | .013 | 10.526 | \*\*\* |  |

## Squared Multiple Correlations: (Group number 1 - Default model)

|  |  |  | Estimate |
| --- | --- | --- | --- |
| 职业召唤 |  |  | .431 |
| 工作绩效 |  |  | .667 |
| 职业认同 |  |  | .720 |
| 个人成长 |  |  | .777 |
| 工作贡献 |  |  | .509 |
| 人际促进 |  |  | .509 |
| 医疗服务绩效 |  |  | .483 |
| B12 |  |  | .502 |
| B13 |  |  | .767 |
| B14 |  |  | .749 |
| B15 |  |  | .503 |
| A12 |  |  | .666 |
| A11 |  |  | .567 |
| A10 |  |  | .577 |
| A9 |  |  | .731 |
| A8 |  |  | .747 |
| A7 |  |  | .730 |
| A6 |  |  | .802 |
| A5 |  |  | .603 |
| A4 |  |  | .581 |
| A3 |  |  | .571 |
| A2 |  |  | .541 |
| A1 |  |  | .579 |

## Matrices (Group number 1 - Default model)

## Total Effects (Group number 1 - Default model)

|  | 性格优势 | 职业召唤 | 工作绩效 |
| --- | --- | --- | --- |
| 职业召唤 | .795 | .000 | .000 |
| 工作绩效 | .513 | .281 | .000 |
| 职业认同 | .829 | .454 | 1.616 |
| 个人成长 | .747 | .409 | 1.456 |
| 工作贡献 | .668 | .366 | 1.303 |
| 人际促进 | .507 | .278 | .988 |
| 医疗服务绩效 | .513 | .281 | 1.000 |
| B12 | .937 | .000 | .000 |
| B13 | .976 | .000 | .000 |
| B14 | 1.011 | .000 | .000 |
| B15 | 1.000 | .000 | .000 |
| A12 | .930 | 1.170 | .000 |
| A11 | .885 | 1.114 | .000 |
| A10 | .698 | .878 | .000 |
| A9 | .801 | 1.008 | .000 |
| A8 | .792 | .997 | .000 |
| A7 | .817 | 1.028 | .000 |
| A6 | .890 | 1.120 | .000 |
| A5 | .749 | .943 | .000 |
| A4 | .755 | .950 | .000 |
| A3 | .824 | 1.037 | .000 |
| A2 | .751 | .945 | .000 |
| A1 | .795 | 1.000 | .000 |

## Standardized Total Effects (Group number 1 - Default model)

|  | 性格优势 | 职业召唤 | 工作绩效 |
| --- | --- | --- | --- |
| 职业召唤 | .657 | .000 | .000 |
| 工作绩效 | .730 | .484 | .000 |
| 职业认同 | .620 | .411 | .849 |
| 个人成长 | .644 | .427 | .881 |
| 工作贡献 | .521 | .345 | .713 |
| 人际促进 | .521 | .345 | .713 |
| 医疗服务绩效 | .507 | .336 | .695 |
| B12 | .708 | .000 | .000 |
| B13 | .876 | .000 | .000 |
| B14 | .866 | .000 | .000 |
| B15 | .709 | .000 | .000 |
| A12 | .536 | .816 | .000 |
| A11 | .494 | .753 | .000 |
| A10 | .499 | .760 | .000 |
| A9 | .561 | .855 | .000 |
| A8 | .567 | .864 | .000 |
| A7 | .561 | .855 | .000 |
| A6 | .588 | .896 | .000 |
| A5 | .510 | .777 | .000 |
| A4 | .501 | .762 | .000 |
| A3 | .496 | .755 | .000 |
| A2 | .483 | .736 | .000 |
| A1 | .500 | .761 | .000 |

## Direct Effects (Group number 1 - Default model)

|  | 性格优势 | 职业召唤 | 工作绩效 |
| --- | --- | --- | --- |
| 职业召唤 | .795 | .000 | .000 |
| 工作绩效 | .290 | .281 | .000 |
| 职业认同 | .000 | .000 | 1.616 |
| 个人成长 | .000 | .000 | 1.456 |
| 工作贡献 | .000 | .000 | 1.303 |
| 人际促进 | .000 | .000 | .988 |
| 医疗服务绩效 | .000 | .000 | 1.000 |
| B12 | .937 | .000 | .000 |
| B13 | .976 | .000 | .000 |
| B14 | 1.011 | .000 | .000 |
| B15 | 1.000 | .000 | .000 |
| A12 | .000 | 1.170 | .000 |
| A11 | .000 | 1.114 | .000 |
| A10 | .000 | .878 | .000 |
| A9 | .000 | 1.008 | .000 |
| A8 | .000 | .997 | .000 |
| A7 | .000 | 1.028 | .000 |
| A6 | .000 | 1.120 | .000 |
| A5 | .000 | .943 | .000 |
| A4 | .000 | .950 | .000 |
| A3 | .000 | 1.037 | .000 |
| A2 | .000 | .945 | .000 |
| A1 | .000 | 1.000 | .000 |

## Standardized Direct Effects (Group number 1 - Default model)

|  | 性格优势 | 职业召唤 | 工作绩效 |
| --- | --- | --- | --- |
| 职业召唤 | .657 | .000 | .000 |
| 工作绩效 | .412 | .484 | .000 |
| 职业认同 | .000 | .000 | .849 |
| 个人成长 | .000 | .000 | .881 |
| 工作贡献 | .000 | .000 | .713 |
| 人际促进 | .000 | .000 | .713 |
| 医疗服务绩效 | .000 | .000 | .695 |
| B12 | .708 | .000 | .000 |
| B13 | .876 | .000 | .000 |
| B14 | .866 | .000 | .000 |
| B15 | .709 | .000 | .000 |
| A12 | .000 | .816 | .000 |
| A11 | .000 | .753 | .000 |
| A10 | .000 | .760 | .000 |
| A9 | .000 | .855 | .000 |
| A8 | .000 | .864 | .000 |
| A7 | .000 | .855 | .000 |
| A6 | .000 | .896 | .000 |
| A5 | .000 | .777 | .000 |
| A4 | .000 | .762 | .000 |
| A3 | .000 | .755 | .000 |
| A2 | .000 | .736 | .000 |
| A1 | .000 | .761 | .000 |

## Indirect Effects (Group number 1 - Default model)

|  | 性格优势 | 职业召唤 | 工作绩效 |
| --- | --- | --- | --- |
| 职业召唤 | .000 | .000 | .000 |
| 工作绩效 | .223 | .000 | .000 |
| 职业认同 | .829 | .454 | .000 |
| 个人成长 | .747 | .409 | .000 |
| 工作贡献 | .668 | .366 | .000 |
| 人际促进 | .507 | .278 | .000 |
| 医疗服务绩效 | .513 | .281 | .000 |
| B12 | .000 | .000 | .000 |
| B13 | .000 | .000 | .000 |
| B14 | .000 | .000 | .000 |
| B15 | .000 | .000 | .000 |
| A12 | .930 | .000 | .000 |
| A11 | .885 | .000 | .000 |
| A10 | .698 | .000 | .000 |
| A9 | .801 | .000 | .000 |
| A8 | .792 | .000 | .000 |
| A7 | .817 | .000 | .000 |
| A6 | .890 | .000 | .000 |
| A5 | .749 | .000 | .000 |
| A4 | .755 | .000 | .000 |
| A3 | .824 | .000 | .000 |
| A2 | .751 | .000 | .000 |
| A1 | .795 | .000 | .000 |

## Standardized Indirect Effects (Group number 1 - Default model)

|  | 性格优势 | 职业召唤 | 工作绩效 |
| --- | --- | --- | --- |
| 职业召唤 | .000 | .000 | .000 |
| 工作绩效 | .318 | .000 | .000 |
| 职业认同 | .620 | .411 | .000 |
| 个人成长 | .644 | .427 | .000 |
| 工作贡献 | .521 | .345 | .000 |
| 人际促进 | .521 | .345 | .000 |
| 医疗服务绩效 | .507 | .336 | .000 |
| B12 | .000 | .000 | .000 |
| B13 | .000 | .000 | .000 |
| B14 | .000 | .000 | .000 |
| B15 | .000 | .000 | .000 |
| A12 | .536 | .000 | .000 |
| A11 | .494 | .000 | .000 |
| A10 | .499 | .000 | .000 |
| A9 | .561 | .000 | .000 |
| A8 | .567 | .000 | .000 |
| A7 | .561 | .000 | .000 |
| A6 | .588 | .000 | .000 |
| A5 | .510 | .000 | .000 |
| A4 | .501 | .000 | .000 |
| A3 | .496 | .000 | .000 |
| A2 | .483 | .000 | .000 |
| A1 | .500 | .000 | .000 |

## Modification Indices (Group number 1 - Default model)

## Covariances: (Group number 1 - Default model)

|  |  |  | M.I. | Par Change |
| --- | --- | --- | --- | --- |
| e21 | <--> | 性格优势 | 6.400 | -.030 |
| e21 | <--> | e22 | 28.542 | .059 |
| e20 | <--> | e22 | 6.856 | -.024 |
| e20 | <--> | e23 | 5.817 | .010 |
| e20 | <--> | e21 | 21.863 | .033 |
| e19 | <--> | 性格优势 | 4.458 | .029 |
| e19 | <--> | e23 | 6.743 | -.016 |
| e19 | <--> | e21 | 4.213 | -.021 |
| e19 | <--> | e20 | 16.100 | -.034 |
| e18 | <--> | e21 | 8.110 | -.019 |
| e17 | <--> | e22 | 7.383 | -.023 |
| e17 | <--> | e21 | 4.707 | -.015 |
| e17 | <--> | e19 | 11.366 | .027 |
| e16 | <--> | e19 | 11.420 | .044 |
| e16 | <--> | e17 | 6.780 | .022 |
| e15 | <--> | e21 | 21.520 | -.034 |
| e15 | <--> | e19 | 4.672 | -.018 |
| e15 | <--> | e18 | 16.373 | .021 |
| e14 | <--> | e20 | 6.566 | .016 |
| e14 | <--> | e16 | 8.277 | -.027 |
| e14 | <--> | e15 | 7.436 | .016 |
| e13 | <--> | e22 | 8.197 | .041 |
| e13 | <--> | e21 | 4.259 | .024 |
| e13 | <--> | e20 | 20.654 | -.044 |
| e13 | <--> | e19 | 42.703 | .090 |
| e13 | <--> | e16 | 14.292 | .054 |
| e13 | <--> | e15 | 6.564 | -.024 |
| e12 | <--> | e23 | 6.374 | .018 |
| e12 | <--> | e21 | 16.648 | .048 |
| e12 | <--> | e18 | 8.890 | -.025 |
| e12 | <--> | e16 | 9.947 | .045 |
| e12 | <--> | e15 | 5.320 | -.022 |
| e12 | <--> | e14 | 4.595 | -.022 |
| e11 | <--> | e23 | 7.620 | .023 |
| e11 | <--> | e21 | 20.551 | .062 |
| e11 | <--> | e19 | 6.917 | .042 |
| e11 | <--> | e15 | 15.325 | -.043 |
| e11 | <--> | e13 | 11.685 | .060 |
| e11 | <--> | e12 | 51.337 | .126 |
| e10 | <--> | 性格优势 | 5.784 | .033 |
| e10 | <--> | e22 | 4.576 | -.027 |
| e10 | <--> | e21 | 6.621 | -.027 |
| e10 | <--> | e19 | 9.507 | .038 |
| e10 | <--> | e13 | 8.545 | .040 |
| e10 | <--> | e11 | 8.777 | .047 |
| e9 | <--> | e23 | 5.635 | -.013 |
| e9 | <--> | e20 | 6.145 | -.018 |
| e9 | <--> | e12 | 12.262 | -.040 |
| e9 | <--> | e10 | 9.394 | .031 |
| e8 | <--> | e23 | 5.933 | -.012 |
| e8 | <--> | e19 | 4.981 | -.022 |
| e8 | <--> | e16 | 19.887 | -.046 |
| e8 | <--> | e15 | 4.460 | .014 |
| e8 | <--> | e12 | 4.237 | -.022 |
| e8 | <--> | e11 | 10.807 | -.041 |
| e8 | <--> | e9 | 11.504 | .027 |
| e7 | <--> | e23 | 6.481 | -.014 |
| e7 | <--> | e20 | 6.303 | -.018 |
| e7 | <--> | e13 | 6.381 | .029 |
| e7 | <--> | e11 | 4.871 | .030 |
| e6 | <--> | e17 | 5.002 | -.014 |
| e6 | <--> | e10 | 15.807 | -.038 |
| e5 | <--> | e16 | 16.721 | -.053 |
| e5 | <--> | e12 | 9.976 | -.044 |
| e5 | <--> | e10 | 5.252 | -.028 |
| e5 | <--> | e8 | 5.028 | .022 |
| e5 | <--> | e6 | 8.792 | .029 |
| e4 | <--> | e21 | 5.001 | -.023 |
| e4 | <--> | e14 | 4.034 | .018 |
| e4 | <--> | e13 | 6.523 | -.034 |
| e4 | <--> | e9 | 7.175 | -.026 |
| e3 | <--> | e23 | 8.904 | .020 |
| e3 | <--> | e21 | 14.198 | .043 |
| e3 | <--> | e16 | 5.757 | .033 |
| e3 | <--> | e14 | 14.922 | -.038 |
| e3 | <--> | e12 | 20.526 | .067 |
| e3 | <--> | e10 | 10.604 | -.043 |
| e3 | <--> | e9 | 11.888 | -.038 |
| e3 | <--> | e7 | 6.794 | -.029 |
| e3 | <--> | e5 | 6.018 | -.033 |
| e2 | <--> | e16 | 21.361 | .057 |
| e2 | <--> | e14 | 8.601 | -.026 |
| e2 | <--> | e12 | 5.882 | .032 |
| e2 | <--> | e8 | 16.602 | -.038 |
| e2 | <--> | e3 | 49.884 | .090 |
| e1 | <--> | e23 | 4.678 | .013 |
| e1 | <--> | e20 | 6.166 | .020 |
| e1 | <--> | e13 | 10.191 | -.041 |
| e1 | <--> | e11 | 11.859 | -.052 |
| e1 | <--> | e8 | 7.096 | .025 |
| e1 | <--> | e7 | 4.836 | -.022 |
| e1 | <--> | e4 | 9.693 | .035 |

## Variances: (Group number 1 - Default model)

|  |  |  | M.I. | Par Change |
| --- | --- | --- | --- | --- |

## Regression Weights: (Group number 1 - Default model)

|  |  |  | M.I. | Par Change |
| --- | --- | --- | --- | --- |
| 职业认同 | <--- | 性格优势 | 6.400 | -.106 |
| 职业认同 | <--- | 职业召唤 | 4.871 | .074 |
| 职业认同 | <--- | 人际促进 | 10.803 | -.134 |
| 职业认同 | <--- | 医疗服务绩效 | 9.675 | -.122 |
| 职业认同 | <--- | B12 | 4.122 | -.061 |
| 职业认同 | <--- | B13 | 15.691 | -.141 |
| 职业认同 | <--- | B14 | 5.908 | -.083 |
| 职业认同 | <--- | A12 | 16.204 | .092 |
| 职业认同 | <--- | A11 | 20.526 | .100 |
| 职业认同 | <--- | A6 | 5.815 | .063 |
| 职业认同 | <--- | A3 | 13.454 | .088 |
| 职业认同 | <--- | A2 | 5.917 | .062 |
| 个人成长 | <--- | 职业召唤 | 4.831 | -.060 |
| 个人成长 | <--- | 职业认同 | 5.348 | .056 |
| 个人成长 | <--- | 工作贡献 | 7.506 | -.070 |
| 个人成长 | <--- | B15 | 11.485 | -.078 |
| 个人成长 | <--- | A11 | 6.966 | -.048 |
| 个人成长 | <--- | A10 | 4.282 | -.048 |
| 个人成长 | <--- | A9 | 9.321 | -.070 |
| 个人成长 | <--- | A7 | 9.423 | -.069 |
| 个人成长 | <--- | A6 | 4.663 | -.046 |
| 工作贡献 | <--- | 性格优势 | 4.458 | .103 |
| 工作贡献 | <--- | 人际促进 | 6.869 | .125 |
| 工作贡献 | <--- | 医疗服务绩效 | 12.012 | .159 |
| 工作贡献 | <--- | B12 | 13.610 | .130 |
| 工作贡献 | <--- | B15 | 33.640 | .191 |
| 工作贡献 | <--- | A11 | 5.615 | .061 |
| 工作贡献 | <--- | A10 | 6.971 | .088 |
| 人际促进 | <--- | B13 | 6.955 | .068 |
| 医疗服务绩效 | <--- | 工作贡献 | 5.074 | .054 |
| 医疗服务绩效 | <--- | B12 | 5.077 | .052 |
| 医疗服务绩效 | <--- | A6 | 4.806 | -.044 |
| B12 | <--- | 工作贡献 | 7.451 | .103 |
| B12 | <--- | B15 | 6.481 | .087 |
| B12 | <--- | A12 | 5.030 | .062 |
| B12 | <--- | A5 | 4.099 | -.067 |
| B12 | <--- | A3 | 4.269 | .060 |
| B12 | <--- | A2 | 11.026 | .103 |
| B13 | <--- | 职业认同 | 8.502 | -.070 |
| B13 | <--- | 工作贡献 | 4.340 | -.052 |
| B13 | <--- | 人际促进 | 6.927 | .087 |
| B13 | <--- | A11 | 9.218 | -.054 |
| B14 | <--- | A3 | 8.498 | -.060 |
| B14 | <--- | A2 | 6.544 | -.056 |
| B15 | <--- | 职业召唤 | 4.226 | .089 |
| B15 | <--- | 工作贡献 | 23.796 | .196 |
| B15 | <--- | B12 | 6.505 | .099 |
| B15 | <--- | A12 | 5.539 | .070 |
| B15 | <--- | A11 | 13.724 | .106 |
| B15 | <--- | A10 | 11.403 | .124 |
| B15 | <--- | A7 | 8.748 | .104 |
| B15 | <--- | A6 | 6.765 | .088 |
| A12 | <--- | 职业认同 | 7.586 | .106 |
| A12 | <--- | A11 | 21.155 | .132 |
| A12 | <--- | A3 | 7.577 | .085 |
| A11 | <--- | 职业认同 | 12.527 | .158 |
| A11 | <--- | 工作贡献 | 8.777 | .138 |
| A11 | <--- | B15 | 8.507 | .123 |
| A11 | <--- | A12 | 15.852 | .137 |
| A11 | <--- | A2 | 4.440 | -.081 |
| A11 | <--- | A1 | 8.619 | -.110 |
| A10 | <--- | 性格优势 | 5.784 | .117 |
| A10 | <--- | 工作贡献 | 7.315 | .097 |
| A10 | <--- | B14 | 5.730 | .094 |
| A10 | <--- | B15 | 12.813 | .117 |
| A9 | <--- | A4 | 7.903 | -.072 |
| A9 | <--- | A3 | 10.088 | -.074 |
| A8 | <--- | 职业认同 | 4.246 | -.057 |
| A8 | <--- | 工作贡献 | 6.352 | -.072 |
| A8 | <--- | B12 | 11.970 | -.096 |
| A8 | <--- | A11 | 4.466 | -.043 |
| A8 | <--- | A2 | 4.518 | -.050 |
| A7 | <--- | 个人成长 | 4.974 | -.076 |
| A7 | <--- | A1 | 4.256 | -.051 |
| A6 | <--- | A10 | 6.389 | -.065 |
| A5 | <--- | B12 | 8.291 | -.102 |
| A3 | <--- | 职业认同 | 5.909 | .090 |
| A3 | <--- | B14 | 8.731 | -.126 |
| A3 | <--- | A12 | 6.320 | .072 |
| A3 | <--- | A10 | 4.242 | -.073 |
| A3 | <--- | A2 | 36.651 | .194 |
| A3 | <--- | A1 | 13.531 | .115 |
| A2 | <--- | B12 | 5.707 | .081 |
| A2 | <--- | B14 | 4.449 | -.081 |
| A2 | <--- | A3 | 21.805 | .125 |
| A1 | <--- | B15 | 4.564 | -.067 |
| A1 | <--- | A11 | 4.868 | -.054 |
| A1 | <--- | A4 | 6.271 | .073 |

## Bootstrap (Group number 1 - Default model)

## Bootstrap standard errors (Group number 1 - Default model)

## Scalar Estimates (Group number 1 - Default model)

## Regression Weights: (Group number 1 - Default model)

| Parameter | | | SE | SE-SE | Mean | Bias | SE-Bias |
| --- | --- | --- | --- | --- | --- | --- | --- |
| 职业召唤 | <--- | 性格优势 | .073 | .001 | .793 | -.002 | .001 |
| 工作绩效 | <--- | 职业召唤 | .046 | .000 | .284 | .003 | .001 |
| 工作绩效 | <--- | 性格优势 | .064 | .001 | .291 | .001 | .001 |
| A1 | <--- | 职业召唤 | .000 | .000 | 1.000 | .000 | .000 |
| A2 | <--- | 职业召唤 | .048 | .000 | .946 | .001 | .001 |
| A3 | <--- | 职业召唤 | .057 | .001 | 1.039 | .002 | .001 |
| A4 | <--- | 职业召唤 | .053 | .001 | .952 | .002 | .001 |
| A5 | <--- | 职业召唤 | .073 | .001 | .947 | .004 | .001 |
| A6 | <--- | 职业召唤 | .063 | .001 | 1.125 | .005 | .001 |
| A7 | <--- | 职业召唤 | .069 | .001 | 1.032 | .004 | .001 |
| A8 | <--- | 职业召唤 | .054 | .001 | 1.001 | .004 | .001 |
| A9 | <--- | 职业召唤 | .062 | .001 | 1.011 | .003 | .001 |
| A10 | <--- | 职业召唤 | .063 | .001 | .880 | .002 | .001 |
| A11 | <--- | 职业召唤 | .085 | .001 | 1.119 | .005 | .001 |
| A12 | <--- | 职业召唤 | .075 | .001 | 1.174 | .004 | .001 |
| B15 | <--- | 性格优势 | .000 | .000 | 1.000 | .000 | .000 |
| B14 | <--- | 性格优势 | .070 | .001 | 1.013 | .002 | .001 |
| B13 | <--- | 性格优势 | .071 | .001 | .978 | .002 | .001 |
| B12 | <--- | 性格优势 | .066 | .001 | .937 | .000 | .001 |
| 医疗服务绩效 | <--- | 工作绩效 | .000 | .000 | 1.000 | .000 | .000 |
| 人际促进 | <--- | 工作绩效 | .055 | .001 | .990 | .002 | .001 |
| 工作贡献 | <--- | 工作绩效 | .091 | .001 | 1.305 | .002 | .001 |
| 个人成长 | <--- | 工作绩效 | .110 | .001 | 1.463 | .007 | .002 |
| 职业认同 | <--- | 工作绩效 | .139 | .001 | 1.623 | .007 | .002 |

## Standardized Regression Weights: (Group number 1 - Default model)

| Parameter | | | SE | SE-SE | Mean | Bias | SE-Bias |
| --- | --- | --- | --- | --- | --- | --- | --- |
| 职业召唤 | <--- | 性格优势 | .049 | .000 | .656 | .000 | .001 |
| 工作绩效 | <--- | 职业召唤 | .065 | .001 | .486 | .002 | .001 |
| 工作绩效 | <--- | 性格优势 | .071 | .001 | .411 | -.001 | .001 |
| A1 | <--- | 职业召唤 | .037 | .000 | .761 | .000 | .001 |
| A2 | <--- | 职业召唤 | .043 | .000 | .736 | .000 | .001 |
| A3 | <--- | 职业召唤 | .037 | .000 | .755 | -.001 | .001 |
| A4 | <--- | 职业召唤 | .029 | .000 | .762 | .000 | .000 |
| A5 | <--- | 职业召唤 | .030 | .000 | .777 | .001 | .000 |
| A6 | <--- | 职业召唤 | .013 | .000 | .896 | .000 | .000 |
| A7 | <--- | 职业召唤 | .021 | .000 | .854 | .000 | .000 |
| A8 | <--- | 职业召唤 | .017 | .000 | .865 | .000 | .000 |
| A9 | <--- | 职业召唤 | .021 | .000 | .855 | .000 | .000 |
| A10 | <--- | 职业召唤 | .035 | .000 | .758 | -.002 | .000 |
| A11 | <--- | 职业召唤 | .030 | .000 | .752 | -.001 | .000 |
| A12 | <--- | 职业召唤 | .028 | .000 | .815 | -.001 | .000 |
| B15 | <--- | 性格优势 | .036 | .000 | .710 | .001 | .001 |
| B14 | <--- | 性格优势 | .019 | .000 | .866 | .000 | .000 |
| B13 | <--- | 性格优势 | .020 | .000 | .876 | .000 | .000 |
| B12 | <--- | 性格优势 | .037 | .000 | .708 | -.001 | .001 |
| 医疗服务绩效 | <--- | 工作绩效 | .036 | .000 | .694 | -.001 | .001 |
| 人际促进 | <--- | 工作绩效 | .036 | .000 | .713 | .000 | .001 |
| 工作贡献 | <--- | 工作绩效 | .035 | .000 | .713 | .000 | .000 |
| 个人成长 | <--- | 工作绩效 | .019 | .000 | .881 | .000 | .000 |
| 职业认同 | <--- | 工作绩效 | .023 | .000 | .849 | .000 | .000 |

## Covariances: (Group number 1 - Default model)

| Parameter | | | SE | SE-SE | Mean | Bias | SE-Bias |
| --- | --- | --- | --- | --- | --- | --- | --- |
| e1 | <--> | e2 | .048 | .000 | .158 | .000 | .001 |
| e17 | <--> | e18 | .011 | .000 | .074 | .000 | .000 |
| e3 | <--> | e4 | .036 | .000 | .119 | .000 | .001 |

## Correlations: (Group number 1 - Default model)

| Parameter | | | SE | SE-SE | Mean | Bias | SE-Bias |
| --- | --- | --- | --- | --- | --- | --- | --- |
| e1 | <--> | e2 | .089 | .001 | .510 | -.007 | .001 |
| e17 | <--> | e18 | .053 | .001 | .534 | .000 | .001 |
| e3 | <--> | e4 | .086 | .001 | .393 | -.001 | .001 |

## Variances: (Group number 1 - Default model)

| Parameter | | | SE | SE-SE | Mean | Bias | SE-Bias |
| --- | --- | --- | --- | --- | --- | --- | --- |
| 性格优势 |  |  | .040 | .000 | .284 | .002 | .001 |
| e22 |  |  | .043 | .000 | .234 | -.001 | .001 |
| e23 |  |  | .008 | .000 | .046 | -.001 | .000 |
| e1 |  |  | .053 | .001 | .299 | -.001 | .001 |
| e2 |  |  | .058 | .001 | .312 | -.001 | .001 |
| e3 |  |  | .059 | .001 | .334 | .000 | .001 |
| e4 |  |  | .037 | .000 | .268 | -.001 | .001 |
| e5 |  |  | .028 | .000 | .239 | -.003 | .000 |
| e6 |  |  | .013 | .000 | .127 | -.001 | .000 |
| e7 |  |  | .023 | .000 | .161 | .000 | .000 |
| e8 |  |  | .017 | .000 | .138 | -.001 | .000 |
| e9 |  |  | .017 | .000 | .153 | -.001 | .000 |
| e10 |  |  | .031 | .000 | .234 | .000 | .000 |
| e11 |  |  | .045 | .000 | .391 | -.001 | .001 |
| e12 |  |  | .045 | .000 | .284 | .000 | .001 |
| e13 |  |  | .033 | .000 | .276 | -.002 | .000 |
| e14 |  |  | .013 | .000 | .096 | -.001 | .000 |
| e15 |  |  | .012 | .000 | .081 | -.001 | .000 |
| e16 |  |  | .032 | .000 | .245 | -.001 | .000 |
| e17 |  |  | .013 | .000 | .149 | .000 | .000 |
| e18 |  |  | .012 | .000 | .130 | -.001 | .000 |
| e19 |  |  | .025 | .000 | .227 | -.002 | .000 |
| e20 |  |  | .012 | .000 | .085 | .000 | .000 |
| e21 |  |  | .021 | .000 | .141 | -.001 | .000 |

## Squared Multiple Correlations: (Group number 1 - Default model)

| Parameter | | | SE | SE-SE | Mean | Bias | SE-Bias |
| --- | --- | --- | --- | --- | --- | --- | --- |
| 职业召唤 |  |  | .064 | .001 | .433 | .002 | .001 |
| 工作绩效 |  |  | .050 | .000 | .671 | .004 | .001 |
| 职业认同 |  |  | .039 | .000 | .721 | .000 | .001 |
| 个人成长 |  |  | .034 | .000 | .776 | .000 | .000 |
| 工作贡献 |  |  | .050 | .000 | .510 | .001 | .001 |
| 人际促进 |  |  | .051 | .001 | .510 | .001 | .001 |
| 医疗服务绩效 |  |  | .049 | .000 | .483 | .000 | .001 |
| B12 |  |  | .052 | .001 | .502 | .000 | .001 |
| B13 |  |  | .035 | .000 | .767 | .000 | .001 |
| B14 |  |  | .032 | .000 | .750 | .001 | .000 |
| B15 |  |  | .051 | .001 | .506 | .003 | .001 |
| A12 |  |  | .045 | .000 | .665 | .000 | .001 |
| A11 |  |  | .044 | .000 | .567 | .000 | .001 |
| A10 |  |  | .053 | .001 | .575 | -.001 | .001 |
| A9 |  |  | .035 | .000 | .731 | .000 | .001 |
| A8 |  |  | .029 | .000 | .748 | .001 | .000 |
| A7 |  |  | .035 | .000 | .730 | .000 | .000 |
| A6 |  |  | .023 | .000 | .803 | .001 | .000 |
| A5 |  |  | .046 | .000 | .605 | .002 | .001 |
| A4 |  |  | .044 | .000 | .582 | .001 | .001 |
| A3 |  |  | .056 | .001 | .571 | .001 | .001 |
| A2 |  |  | .062 | .001 | .543 | .001 | .001 |
| A1 |  |  | .055 | .001 | .581 | .001 | .001 |

## Matrices (Group number 1 - Default model)

## Total Effects - Standard Errors (Group number 1 - Default model)

|  | 性格优势 | 职业召唤 | 工作绩效 |
| --- | --- | --- | --- |
| 职业召唤 | .073 | .000 | .000 |
| 工作绩效 | .064 | .046 | .000 |
| 职业认同 | .079 | .083 | .139 |
| 个人成长 | .073 | .070 | .110 |
| 工作贡献 | .077 | .062 | .091 |
| 人际促进 | .061 | .045 | .055 |
| 医疗服务绩效 | .064 | .046 | .000 |
| B12 | .066 | .000 | .000 |
| B13 | .071 | .000 | .000 |
| B14 | .070 | .000 | .000 |
| B15 | .000 | .000 | .000 |
| A12 | .074 | .075 | .000 |
| A11 | .077 | .085 | .000 |
| A10 | .071 | .063 | .000 |
| A9 | .072 | .062 | .000 |
| A8 | .068 | .054 | .000 |
| A7 | .070 | .069 | .000 |
| A6 | .073 | .063 | .000 |
| A5 | .074 | .073 | .000 |
| A4 | .076 | .053 | .000 |
| A3 | .072 | .057 | .000 |
| A2 | .068 | .048 | .000 |
| A1 | .073 | .000 | .000 |

## Standardized Total Effects - Standard Errors (Group number 1 - Default model)

|  | 性格优势 | 职业召唤 | 工作绩效 |
| --- | --- | --- | --- |
| 职业召唤 | .049 | .000 | .000 |
| 工作绩效 | .040 | .065 | .000 |
| 职业认同 | .035 | .060 | .023 |
| 个人成长 | .035 | .059 | .019 |
| 工作贡献 | .047 | .046 | .035 |
| 人际促进 | .045 | .046 | .036 |
| 医疗服务绩效 | .045 | .044 | .036 |
| B12 | .037 | .000 | .000 |
| B13 | .020 | .000 | .000 |
| B14 | .019 | .000 | .000 |
| B15 | .036 | .000 | .000 |
| A12 | .042 | .028 | .000 |
| A11 | .041 | .030 | .000 |
| A10 | .048 | .035 | .000 |
| A9 | .043 | .021 | .000 |
| A8 | .044 | .017 | .000 |
| A7 | .043 | .021 | .000 |
| A6 | .044 | .013 | .000 |
| A5 | .043 | .030 | .000 |
| A4 | .046 | .029 | .000 |
| A3 | .042 | .037 | .000 |
| A2 | .043 | .043 | .000 |
| A1 | .044 | .037 | .000 |

## Direct Effects - Standard Errors (Group number 1 - Default model)

|  | 性格优势 | 职业召唤 | 工作绩效 |
| --- | --- | --- | --- |
| 职业召唤 | .073 | .000 | .000 |
| 工作绩效 | .064 | .046 | .000 |
| 职业认同 | .000 | .000 | .139 |
| 个人成长 | .000 | .000 | .110 |
| 工作贡献 | .000 | .000 | .091 |
| 人际促进 | .000 | .000 | .055 |
| 医疗服务绩效 | .000 | .000 | .000 |
| B12 | .066 | .000 | .000 |
| B13 | .071 | .000 | .000 |
| B14 | .070 | .000 | .000 |
| B15 | .000 | .000 | .000 |
| A12 | .000 | .075 | .000 |
| A11 | .000 | .085 | .000 |
| A10 | .000 | .063 | .000 |
| A9 | .000 | .062 | .000 |
| A8 | .000 | .054 | .000 |
| A7 | .000 | .069 | .000 |
| A6 | .000 | .063 | .000 |
| A5 | .000 | .073 | .000 |
| A4 | .000 | .053 | .000 |
| A3 | .000 | .057 | .000 |
| A2 | .000 | .048 | .000 |
| A1 | .000 | .000 | .000 |

## Standardized Direct Effects - Standard Errors (Group number 1 - Default model)

|  | 性格优势 | 职业召唤 | 工作绩效 |
| --- | --- | --- | --- |
| 职业召唤 | .049 | .000 | .000 |
| 工作绩效 | .071 | .065 | .000 |
| 职业认同 | .000 | .000 | .023 |
| 个人成长 | .000 | .000 | .019 |
| 工作贡献 | .000 | .000 | .035 |
| 人际促进 | .000 | .000 | .036 |
| 医疗服务绩效 | .000 | .000 | .036 |
| B12 | .037 | .000 | .000 |
| B13 | .020 | .000 | .000 |
| B14 | .019 | .000 | .000 |
| B15 | .036 | .000 | .000 |
| A12 | .000 | .028 | .000 |
| A11 | .000 | .030 | .000 |
| A10 | .000 | .035 | .000 |
| A9 | .000 | .021 | .000 |
| A8 | .000 | .017 | .000 |
| A7 | .000 | .021 | .000 |
| A6 | .000 | .013 | .000 |
| A5 | .000 | .030 | .000 |
| A4 | .000 | .029 | .000 |
| A3 | .000 | .037 | .000 |
| A2 | .000 | .043 | .000 |
| A1 | .000 | .037 | .000 |

## Indirect Effects - Standard Errors (Group number 1 - Default model)

|  | 性格优势 | 职业召唤 | 工作绩效 |
| --- | --- | --- | --- |
| 职业召唤 | .000 | .000 | .000 |
| 工作绩效 | .037 | .000 | .000 |
| 职业认同 | .079 | .083 | .000 |
| 个人成长 | .073 | .070 | .000 |
| 工作贡献 | .077 | .062 | .000 |
| 人际促进 | .061 | .045 | .000 |
| 医疗服务绩效 | .064 | .046 | .000 |
| B12 | .000 | .000 | .000 |
| B13 | .000 | .000 | .000 |
| B14 | .000 | .000 | .000 |
| B15 | .000 | .000 | .000 |
| A12 | .074 | .000 | .000 |
| A11 | .077 | .000 | .000 |
| A10 | .071 | .000 | .000 |
| A9 | .072 | .000 | .000 |
| A8 | .068 | .000 | .000 |
| A7 | .070 | .000 | .000 |
| A6 | .073 | .000 | .000 |
| A5 | .074 | .000 | .000 |
| A4 | .076 | .000 | .000 |
| A3 | .072 | .000 | .000 |
| A2 | .068 | .000 | .000 |
| A1 | .073 | .000 | .000 |

## Standardized Indirect Effects - Standard Errors (Group number 1 - Default model)

|  | 性格优势 | 职业召唤 | 工作绩效 |
| --- | --- | --- | --- |
| 职业召唤 | .000 | .000 | .000 |
| 工作绩效 | .048 | .000 | .000 |
| 职业认同 | .035 | .060 | .000 |
| 个人成长 | .035 | .059 | .000 |
| 工作贡献 | .047 | .046 | .000 |
| 人际促进 | .045 | .046 | .000 |
| 医疗服务绩效 | .045 | .044 | .000 |
| B12 | .000 | .000 | .000 |
| B13 | .000 | .000 | .000 |
| B14 | .000 | .000 | .000 |
| B15 | .000 | .000 | .000 |
| A12 | .042 | .000 | .000 |
| A11 | .041 | .000 | .000 |
| A10 | .048 | .000 | .000 |
| A9 | .043 | .000 | .000 |
| A8 | .044 | .000 | .000 |
| A7 | .043 | .000 | .000 |
| A6 | .044 | .000 | .000 |
| A5 | .043 | .000 | .000 |
| A4 | .046 | .000 | .000 |
| A3 | .042 | .000 | .000 |
| A2 | .043 | .000 | .000 |
| A1 | .044 | .000 | .000 |

## Bootstrap Confidence (Group number 1 - Default model)

## Percentile method (Group number 1 - Default model)

## 95% confidence intervals (percentile method)

## Scalar Estimates (Group number 1 - Default model)

## Regression Weights: (Group number 1 - Default model)

| Parameter | | | Estimate | Lower | Upper | P |
| --- | --- | --- | --- | --- | --- | --- |
| 职业召唤 | <--- | 性格优势 | .795 | .656 | .944 | .000 |
| 工作绩效 | <--- | 职业召唤 | .281 | .200 | .381 | .000 |
| 工作绩效 | <--- | 性格优势 | .290 | .172 | .421 | .000 |
| A1 | <--- | 职业召唤 | 1.000 | 1.000 | 1.000 | ... |
| A2 | <--- | 职业召唤 | .945 | .855 | 1.045 | .000 |
| A3 | <--- | 职业召唤 | 1.037 | .931 | 1.157 | .000 |
| A4 | <--- | 职业召唤 | .950 | .852 | 1.062 | .000 |
| A5 | <--- | 职业召唤 | .943 | .807 | 1.094 | .000 |
| A6 | <--- | 职业召唤 | 1.120 | 1.008 | 1.259 | .000 |
| A7 | <--- | 职业召唤 | 1.028 | .907 | 1.175 | .000 |
| A8 | <--- | 职业召唤 | .997 | .902 | 1.115 | .000 |
| A9 | <--- | 职业召唤 | 1.008 | .891 | 1.137 | .000 |
| A10 | <--- | 职业召唤 | .878 | .762 | 1.010 | .000 |
| A11 | <--- | 职业召唤 | 1.114 | .960 | 1.295 | .000 |
| A12 | <--- | 职业召唤 | 1.170 | 1.033 | 1.332 | .000 |
| B15 | <--- | 性格优势 | 1.000 | 1.000 | 1.000 | ... |
| B14 | <--- | 性格优势 | 1.011 | .888 | 1.162 | .000 |
| B13 | <--- | 性格优势 | .976 | .842 | 1.122 | .000 |
| B12 | <--- | 性格优势 | .937 | .812 | 1.071 | .000 |
| 医疗服务绩效 | <--- | 工作绩效 | 1.000 | 1.000 | 1.000 | ... |
| 人际促进 | <--- | 工作绩效 | .988 | .888 | 1.104 | .000 |
| 工作贡献 | <--- | 工作绩效 | 1.303 | 1.140 | 1.501 | .000 |
| 个人成长 | <--- | 工作绩效 | 1.456 | 1.273 | 1.699 | .000 |
| 职业认同 | <--- | 工作绩效 | 1.616 | 1.384 | 1.923 | .000 |

## Standardized Regression Weights: (Group number 1 - Default model)

| Parameter | | | Estimate | Lower | Upper | P |
| --- | --- | --- | --- | --- | --- | --- |
| 职业召唤 | <--- | 性格优势 | .657 | .555 | .747 | .000 |
| 工作绩效 | <--- | 职业召唤 | .484 | .359 | .612 | .000 |
| 工作绩效 | <--- | 性格优势 | .412 | .269 | .546 | .000 |
| A1 | <--- | 职业召唤 | .761 | .681 | .826 | .000 |
| A2 | <--- | 职业召唤 | .736 | .645 | .813 | .000 |
| A3 | <--- | 职业召唤 | .755 | .676 | .824 | .000 |
| A4 | <--- | 职业召唤 | .762 | .705 | .816 | .000 |
| A5 | <--- | 职业召唤 | .777 | .714 | .832 | .000 |
| A6 | <--- | 职业召唤 | .896 | .869 | .920 | .000 |
| A7 | <--- | 职业召唤 | .855 | .810 | .891 | .000 |
| A8 | <--- | 职业召唤 | .864 | .830 | .896 | .000 |
| A9 | <--- | 职业召唤 | .855 | .810 | .892 | .000 |
| A10 | <--- | 职业召唤 | .760 | .684 | .823 | .000 |
| A11 | <--- | 职业召唤 | .753 | .690 | .807 | .000 |
| A12 | <--- | 职业召唤 | .816 | .754 | .865 | .000 |
| B15 | <--- | 性格优势 | .709 | .637 | .776 | .000 |
| B14 | <--- | 性格优势 | .866 | .827 | .901 | .000 |
| B13 | <--- | 性格优势 | .876 | .832 | .913 | .000 |
| B12 | <--- | 性格优势 | .708 | .632 | .776 | .000 |
| 医疗服务绩效 | <--- | 工作绩效 | .695 | .622 | .760 | .000 |
| 人际促进 | <--- | 工作绩效 | .713 | .639 | .779 | .000 |
| 工作贡献 | <--- | 工作绩效 | .713 | .639 | .777 | .000 |
| 个人成长 | <--- | 工作绩效 | .881 | .840 | .916 | .000 |
| 职业认同 | <--- | 工作绩效 | .849 | .801 | .890 | .000 |

## Covariances: (Group number 1 - Default model)

| Parameter | | | Estimate | Lower | Upper | P |
| --- | --- | --- | --- | --- | --- | --- |
| e1 | <--> | e2 | .158 | .079 | .262 | .000 |
| e17 | <--> | e18 | .075 | .054 | .096 | .000 |
| e3 | <--> | e4 | .118 | .055 | .193 | .000 |

## Correlations: (Group number 1 - Default model)

| Parameter | | | Estimate | Lower | Upper | P |
| --- | --- | --- | --- | --- | --- | --- |
| e1 | <--> | e2 | .517 | .326 | .670 | .000 |
| e17 | <--> | e18 | .534 | .428 | .634 | .000 |
| e3 | <--> | e4 | .394 | .213 | .557 | .000 |

## Variances: (Group number 1 - Default model)

| Parameter | | | Estimate | Lower | Upper | P |
| --- | --- | --- | --- | --- | --- | --- |
| 性格优势 |  |  | .282 | .208 | .365 | .000 |
| e22 |  |  | .235 | .158 | .326 | .000 |
| e23 |  |  | .046 | .031 | .063 | .000 |
| e1 |  |  | .300 | .208 | .414 | .000 |
| e2 |  |  | .313 | .212 | .441 | .000 |
| e3 |  |  | .334 | .230 | .462 | .000 |
| e4 |  |  | .269 | .200 | .343 | .000 |
| e5 |  |  | .242 | .188 | .296 | .000 |
| e6 |  |  | .128 | .102 | .153 | .000 |
| e7 |  |  | .161 | .120 | .210 | .000 |
| e8 |  |  | .139 | .108 | .174 | .000 |
| e9 |  |  | .154 | .120 | .189 | .000 |
| e10 |  |  | .234 | .176 | .298 | .000 |
| e11 |  |  | .392 | .306 | .486 | .000 |
| e12 |  |  | .284 | .208 | .382 | .000 |
| e13 |  |  | .278 | .215 | .344 | .000 |
| e14 |  |  | .097 | .072 | .122 | .000 |
| e15 |  |  | .082 | .059 | .106 | .000 |
| e16 |  |  | .246 | .188 | .311 | .000 |
| e17 |  |  | .149 | .125 | .175 | .000 |
| e18 |  |  | .131 | .107 | .155 | .000 |
| e19 |  |  | .228 | .181 | .277 | .000 |
| e20 |  |  | .085 | .062 | .110 | .000 |
| e21 |  |  | .141 | .102 | .183 | .000 |

## Squared Multiple Correlations: (Group number 1 - Default model)

| Parameter | | | Estimate | Lower | Upper | P |
| --- | --- | --- | --- | --- | --- | --- |
| 职业召唤 |  |  | .431 | .308 | .558 | .000 |
| 工作绩效 |  |  | .667 | .570 | .763 | .000 |
| 职业认同 |  |  | .720 | .641 | .792 | .000 |
| 个人成长 |  |  | .777 | .705 | .839 | .000 |
| 工作贡献 |  |  | .509 | .409 | .604 | .000 |
| 人际促进 |  |  | .509 | .408 | .606 | .000 |
| 医疗服务绩效 |  |  | .483 | .387 | .578 | .000 |
| B12 |  |  | .502 | .399 | .602 | .000 |
| B13 |  |  | .767 | .693 | .834 | .000 |
| B14 |  |  | .749 | .683 | .812 | .000 |
| B15 |  |  | .503 | .405 | .602 | .000 |
| A12 |  |  | .666 | .568 | .749 | .000 |
| A11 |  |  | .567 | .476 | .651 | .000 |
| A10 |  |  | .577 | .468 | .677 | .000 |
| A9 |  |  | .731 | .655 | .795 | .000 |
| A8 |  |  | .747 | .689 | .803 | .000 |
| A7 |  |  | .730 | .656 | .793 | .000 |
| A6 |  |  | .802 | .755 | .846 | .000 |
| A5 |  |  | .603 | .510 | .692 | .000 |
| A4 |  |  | .581 | .497 | .667 | .000 |
| A3 |  |  | .571 | .457 | .679 | .000 |
| A2 |  |  | .541 | .416 | .660 | .000 |
| A1 |  |  | .579 | .464 | .681 | .000 |

## Matrices (Group number 1 - Default model)

## Total Effects (Group number 1 - Default model)

## Total Effects - Lower Bounds (PC) (Group number 1 - Default model)

|  | 性格优势 | 职业召唤 | 工作绩效 |
| --- | --- | --- | --- |
| 职业召唤 | .656 | .000 | .000 |
| 工作绩效 | .394 | .200 | .000 |
| 职业认同 | .683 | .311 | 1.384 |
| 个人成长 | .612 | .285 | 1.273 |
| 工作贡献 | .523 | .257 | 1.140 |
| 人际促进 | .395 | .198 | .888 |
| 医疗服务绩效 | .394 | .200 | 1.000 |
| B12 | .812 | .000 | .000 |
| B13 | .842 | .000 | .000 |
| B14 | .888 | .000 | .000 |
| B15 | 1.000 | .000 | .000 |
| A12 | .787 | 1.033 | .000 |
| A11 | .738 | .960 | .000 |
| A10 | .561 | .762 | .000 |
| A9 | .666 | .891 | .000 |
| A8 | .665 | .902 | .000 |
| A7 | .683 | .907 | .000 |
| A6 | .750 | 1.008 | .000 |
| A5 | .608 | .807 | .000 |
| A4 | .610 | .852 | .000 |
| A3 | .687 | .931 | .000 |
| A2 | .621 | .855 | .000 |
| A1 | .656 | 1.000 | .000 |

## Total Effects - Upper Bounds (PC) (Group number 1 - Default model)

|  | 性格优势 | 职业召唤 | 工作绩效 |
| --- | --- | --- | --- |
| 职业召唤 | .944 | .000 | .000 |
| 工作绩效 | .647 | .381 | .000 |
| 职业认同 | .992 | .632 | 1.923 |
| 个人成长 | .903 | .560 | 1.699 |
| 工作贡献 | .825 | .502 | 1.501 |
| 人际促进 | .635 | .377 | 1.104 |
| 医疗服务绩效 | .647 | .381 | 1.000 |
| B12 | 1.071 | .000 | .000 |
| B13 | 1.122 | .000 | .000 |
| B14 | 1.162 | .000 | .000 |
| B15 | 1.000 | .000 | .000 |
| A12 | 1.077 | 1.332 | .000 |
| A11 | 1.041 | 1.295 | .000 |
| A10 | .838 | 1.010 | .000 |
| A9 | .946 | 1.137 | .000 |
| A8 | .932 | 1.115 | .000 |
| A7 | .960 | 1.175 | .000 |
| A6 | 1.035 | 1.259 | .000 |
| A5 | .898 | 1.094 | .000 |
| A4 | .912 | 1.062 | .000 |
| A3 | .971 | 1.157 | .000 |
| A2 | .887 | 1.045 | .000 |
| A1 | .944 | 1.000 | .000 |

## Total Effects - Two Tailed Significance (PC) (Group number 1 - Default model)

|  | 性格优势 | 职业召唤 | 工作绩效 |
| --- | --- | --- | --- |
| 职业召唤 | .000 | ... | ... |
| 工作绩效 | .000 | .000 | ... |
| 职业认同 | .000 | .000 | .000 |
| 个人成长 | .000 | .000 | .000 |
| 工作贡献 | .000 | .000 | .000 |
| 人际促进 | .000 | .000 | .000 |
| 医疗服务绩效 | .000 | .000 | ... |
| B12 | .000 | ... | ... |
| B13 | .000 | ... | ... |
| B14 | .000 | ... | ... |
| B15 | ... | ... | ... |
| A12 | .000 | .000 | ... |
| A11 | .000 | .000 | ... |
| A10 | .000 | .000 | ... |
| A9 | .000 | .000 | ... |
| A8 | .000 | .000 | ... |
| A7 | .000 | .000 | ... |
| A6 | .000 | .000 | ... |
| A5 | .000 | .000 | ... |
| A4 | .000 | .000 | ... |
| A3 | .000 | .000 | ... |
| A2 | .000 | .000 | ... |
| A1 | .000 | ... | ... |

## Standardized Total Effects (Group number 1 - Default model)

## Standardized Total Effects - Lower Bounds (PC) (Group number 1 - Default model)

|  | 性格优势 | 职业召唤 | 工作绩效 |
| --- | --- | --- | --- |
| 职业召唤 | .555 | .000 | .000 |
| 工作绩效 | .646 | .359 | .000 |
| 职业认同 | .549 | .297 | .801 |
| 个人成长 | .571 | .315 | .840 |
| 工作贡献 | .426 | .255 | .639 |
| 人际促进 | .432 | .257 | .639 |
| 医疗服务绩效 | .417 | .252 | .622 |
| B12 | .632 | .000 | .000 |
| B13 | .832 | .000 | .000 |
| B14 | .827 | .000 | .000 |
| B15 | .637 | .000 | .000 |
| A12 | .450 | .754 | .000 |
| A11 | .414 | .690 | .000 |
| A10 | .403 | .684 | .000 |
| A9 | .473 | .810 | .000 |
| A8 | .477 | .830 | .000 |
| A7 | .473 | .810 | .000 |
| A6 | .497 | .869 | .000 |
| A5 | .425 | .714 | .000 |
| A4 | .409 | .705 | .000 |
| A3 | .411 | .676 | .000 |
| A2 | .396 | .645 | .000 |
| A1 | .414 | .681 | .000 |

## Standardized Total Effects - Upper Bounds (PC) (Group number 1 - Default model)

|  | 性格优势 | 职业召唤 | 工作绩效 |
| --- | --- | --- | --- |
| 职业召唤 | .747 | .000 | .000 |
| 工作绩效 | .807 | .612 | .000 |
| 职业认同 | .686 | .531 | .890 |
| 个人成长 | .709 | .544 | .916 |
| 工作贡献 | .610 | .434 | .777 |
| 人际促进 | .609 | .436 | .779 |
| 医疗服务绩效 | .592 | .423 | .760 |
| B12 | .776 | .000 | .000 |
| B13 | .913 | .000 | .000 |
| B14 | .901 | .000 | .000 |
| B15 | .776 | .000 | .000 |
| A12 | .615 | .865 | .000 |
| A11 | .573 | .807 | .000 |
| A10 | .592 | .823 | .000 |
| A9 | .645 | .892 | .000 |
| A8 | .650 | .896 | .000 |
| A7 | .641 | .891 | .000 |
| A6 | .670 | .920 | .000 |
| A5 | .592 | .832 | .000 |
| A4 | .588 | .816 | .000 |
| A3 | .576 | .824 | .000 |
| A2 | .566 | .813 | .000 |
| A1 | .585 | .826 | .000 |

## Standardized Total Effects - Two Tailed Significance (PC) (Group number 1 - Default model)

|  | 性格优势 | 职业召唤 | 工作绩效 |
| --- | --- | --- | --- |
| 职业召唤 | .000 | ... | ... |
| 工作绩效 | .000 | .000 | ... |
| 职业认同 | .000 | .000 | .000 |
| 个人成长 | .000 | .000 | .000 |
| 工作贡献 | .000 | .000 | .000 |
| 人际促进 | .000 | .000 | .000 |
| 医疗服务绩效 | .000 | .000 | .000 |
| B12 | .000 | ... | ... |
| B13 | .000 | ... | ... |
| B14 | .000 | ... | ... |
| B15 | .000 | ... | ... |
| A12 | .000 | .000 | ... |
| A11 | .000 | .000 | ... |
| A10 | .000 | .000 | ... |
| A9 | .000 | .000 | ... |
| A8 | .000 | .000 | ... |
| A7 | .000 | .000 | ... |
| A6 | .000 | .000 | ... |
| A5 | .000 | .000 | ... |
| A4 | .000 | .000 | ... |
| A3 | .000 | .000 | ... |
| A2 | .000 | .000 | ... |
| A1 | .000 | .000 | ... |

## Direct Effects (Group number 1 - Default model)

## Direct Effects - Lower Bounds (PC) (Group number 1 - Default model)

|  | 性格优势 | 职业召唤 | 工作绩效 |
| --- | --- | --- | --- |
| 职业召唤 | .656 | .000 | .000 |
| 工作绩效 | .172 | .200 | .000 |
| 职业认同 | .000 | .000 | 1.384 |
| 个人成长 | .000 | .000 | 1.273 |
| 工作贡献 | .000 | .000 | 1.140 |
| 人际促进 | .000 | .000 | .888 |
| 医疗服务绩效 | .000 | .000 | 1.000 |
| B12 | .812 | .000 | .000 |
| B13 | .842 | .000 | .000 |
| B14 | .888 | .000 | .000 |
| B15 | 1.000 | .000 | .000 |
| A12 | .000 | 1.033 | .000 |
| A11 | .000 | .960 | .000 |
| A10 | .000 | .762 | .000 |
| A9 | .000 | .891 | .000 |
| A8 | .000 | .902 | .000 |
| A7 | .000 | .907 | .000 |
| A6 | .000 | 1.008 | .000 |
| A5 | .000 | .807 | .000 |
| A4 | .000 | .852 | .000 |
| A3 | .000 | .931 | .000 |
| A2 | .000 | .855 | .000 |
| A1 | .000 | 1.000 | .000 |

## Direct Effects - Upper Bounds (PC) (Group number 1 - Default model)

|  | 性格优势 | 职业召唤 | 工作绩效 |
| --- | --- | --- | --- |
| 职业召唤 | .944 | .000 | .000 |
| 工作绩效 | .421 | .381 | .000 |
| 职业认同 | .000 | .000 | 1.923 |
| 个人成长 | .000 | .000 | 1.699 |
| 工作贡献 | .000 | .000 | 1.501 |
| 人际促进 | .000 | .000 | 1.104 |
| 医疗服务绩效 | .000 | .000 | 1.000 |
| B12 | 1.071 | .000 | .000 |
| B13 | 1.122 | .000 | .000 |
| B14 | 1.162 | .000 | .000 |
| B15 | 1.000 | .000 | .000 |
| A12 | .000 | 1.332 | .000 |
| A11 | .000 | 1.295 | .000 |
| A10 | .000 | 1.010 | .000 |
| A9 | .000 | 1.137 | .000 |
| A8 | .000 | 1.115 | .000 |
| A7 | .000 | 1.175 | .000 |
| A6 | .000 | 1.259 | .000 |
| A5 | .000 | 1.094 | .000 |
| A4 | .000 | 1.062 | .000 |
| A3 | .000 | 1.157 | .000 |
| A2 | .000 | 1.045 | .000 |
| A1 | .000 | 1.000 | .000 |

## Direct Effects - Two Tailed Significance (PC) (Group number 1 - Default model)

|  | 性格优势 | 职业召唤 | 工作绩效 |
| --- | --- | --- | --- |
| 职业召唤 | .000 | ... | ... |
| 工作绩效 | .000 | .000 | ... |
| 职业认同 | ... | ... | .000 |
| 个人成长 | ... | ... | .000 |
| 工作贡献 | ... | ... | .000 |
| 人际促进 | ... | ... | .000 |
| 医疗服务绩效 | ... | ... | ... |
| B12 | .000 | ... | ... |
| B13 | .000 | ... | ... |
| B14 | .000 | ... | ... |
| B15 | ... | ... | ... |
| A12 | ... | .000 | ... |
| A11 | ... | .000 | ... |
| A10 | ... | .000 | ... |
| A9 | ... | .000 | ... |
| A8 | ... | .000 | ... |
| A7 | ... | .000 | ... |
| A6 | ... | .000 | ... |
| A5 | ... | .000 | ... |
| A4 | ... | .000 | ... |
| A3 | ... | .000 | ... |
| A2 | ... | .000 | ... |
| A1 | ... | ... | ... |

## Standardized Direct Effects (Group number 1 - Default model)

## Standardized Direct Effects - Lower Bounds (PC) (Group number 1 - Default model)

|  | 性格优势 | 职业召唤 | 工作绩效 |
| --- | --- | --- | --- |
| 职业召唤 | .555 | .000 | .000 |
| 工作绩效 | .269 | .359 | .000 |
| 职业认同 | .000 | .000 | .801 |
| 个人成长 | .000 | .000 | .840 |
| 工作贡献 | .000 | .000 | .639 |
| 人际促进 | .000 | .000 | .639 |
| 医疗服务绩效 | .000 | .000 | .622 |
| B12 | .632 | .000 | .000 |
| B13 | .832 | .000 | .000 |
| B14 | .827 | .000 | .000 |
| B15 | .637 | .000 | .000 |
| A12 | .000 | .754 | .000 |
| A11 | .000 | .690 | .000 |
| A10 | .000 | .684 | .000 |
| A9 | .000 | .810 | .000 |
| A8 | .000 | .830 | .000 |
| A7 | .000 | .810 | .000 |
| A6 | .000 | .869 | .000 |
| A5 | .000 | .714 | .000 |
| A4 | .000 | .705 | .000 |
| A3 | .000 | .676 | .000 |
| A2 | .000 | .645 | .000 |
| A1 | .000 | .681 | .000 |

## Standardized Direct Effects - Upper Bounds (PC) (Group number 1 - Default model)

|  | 性格优势 | 职业召唤 | 工作绩效 |
| --- | --- | --- | --- |
| 职业召唤 | .747 | .000 | .000 |
| 工作绩效 | .546 | .612 | .000 |
| 职业认同 | .000 | .000 | .890 |
| 个人成长 | .000 | .000 | .916 |
| 工作贡献 | .000 | .000 | .777 |
| 人际促进 | .000 | .000 | .779 |
| 医疗服务绩效 | .000 | .000 | .760 |
| B12 | .776 | .000 | .000 |
| B13 | .913 | .000 | .000 |
| B14 | .901 | .000 | .000 |
| B15 | .776 | .000 | .000 |
| A12 | .000 | .865 | .000 |
| A11 | .000 | .807 | .000 |
| A10 | .000 | .823 | .000 |
| A9 | .000 | .892 | .000 |
| A8 | .000 | .896 | .000 |
| A7 | .000 | .891 | .000 |
| A6 | .000 | .920 | .000 |
| A5 | .000 | .832 | .000 |
| A4 | .000 | .816 | .000 |
| A3 | .000 | .824 | .000 |
| A2 | .000 | .813 | .000 |
| A1 | .000 | .826 | .000 |

## Standardized Direct Effects - Two Tailed Significance (PC) (Group number 1 - Default model)

|  | 性格优势 | 职业召唤 | 工作绩效 |
| --- | --- | --- | --- |
| 职业召唤 | .000 | ... | ... |
| 工作绩效 | .000 | .000 | ... |
| 职业认同 | ... | ... | .000 |
| 个人成长 | ... | ... | .000 |
| 工作贡献 | ... | ... | .000 |
| 人际促进 | ... | ... | .000 |
| 医疗服务绩效 | ... | ... | .000 |
| B12 | .000 | ... | ... |
| B13 | .000 | ... | ... |
| B14 | .000 | ... | ... |
| B15 | .000 | ... | ... |
| A12 | ... | .000 | ... |
| A11 | ... | .000 | ... |
| A10 | ... | .000 | ... |
| A9 | ... | .000 | ... |
| A8 | ... | .000 | ... |
| A7 | ... | .000 | ... |
| A6 | ... | .000 | ... |
| A5 | ... | .000 | ... |
| A4 | ... | .000 | ... |
| A3 | ... | .000 | ... |
| A2 | ... | .000 | ... |
| A1 | ... | .000 | ... |

## Indirect Effects (Group number 1 - Default model)

## Indirect Effects - Lower Bounds (PC) (Group number 1 - Default model)

|  | 性格优势 | 职业召唤 | 工作绩效 |
| --- | --- | --- | --- |
| 职业召唤 | .000 | .000 | .000 |
| 工作绩效 | .157 | .000 | .000 |
| 职业认同 | .683 | .311 | .000 |
| 个人成长 | .612 | .285 | .000 |
| 工作贡献 | .523 | .257 | .000 |
| 人际促进 | .395 | .198 | .000 |
| 医疗服务绩效 | .394 | .200 | .000 |
| B12 | .000 | .000 | .000 |
| B13 | .000 | .000 | .000 |
| B14 | .000 | .000 | .000 |
| B15 | .000 | .000 | .000 |
| A12 | .787 | .000 | .000 |
| A11 | .738 | .000 | .000 |
| A10 | .561 | .000 | .000 |
| A9 | .666 | .000 | .000 |
| A8 | .665 | .000 | .000 |
| A7 | .683 | .000 | .000 |
| A6 | .750 | .000 | .000 |
| A5 | .608 | .000 | .000 |
| A4 | .610 | .000 | .000 |
| A3 | .687 | .000 | .000 |
| A2 | .621 | .000 | .000 |
| A1 | .656 | .000 | .000 |

## Indirect Effects - Upper Bounds (PC) (Group number 1 - Default model)

|  | 性格优势 | 职业召唤 | 工作绩效 |
| --- | --- | --- | --- |
| 职业召唤 | .000 | .000 | .000 |
| 工作绩效 | .302 | .000 | .000 |
| 职业认同 | .992 | .632 | .000 |
| 个人成长 | .903 | .560 | .000 |
| 工作贡献 | .825 | .502 | .000 |
| 人际促进 | .635 | .377 | .000 |
| 医疗服务绩效 | .647 | .381 | .000 |
| B12 | .000 | .000 | .000 |
| B13 | .000 | .000 | .000 |
| B14 | .000 | .000 | .000 |
| B15 | .000 | .000 | .000 |
| A12 | 1.077 | .000 | .000 |
| A11 | 1.041 | .000 | .000 |
| A10 | .838 | .000 | .000 |
| A9 | .946 | .000 | .000 |
| A8 | .932 | .000 | .000 |
| A7 | .960 | .000 | .000 |
| A6 | 1.035 | .000 | .000 |
| A5 | .898 | .000 | .000 |
| A4 | .912 | .000 | .000 |
| A3 | .971 | .000 | .000 |
| A2 | .887 | .000 | .000 |
| A1 | .944 | .000 | .000 |

## Indirect Effects - Two Tailed Significance (PC) (Group number 1 - Default model)

|  | 性格优势 | 职业召唤 | 工作绩效 |
| --- | --- | --- | --- |
| 职业召唤 | ... | ... | ... |
| 工作绩效 | .000 | ... | ... |
| 职业认同 | .000 | .000 | ... |
| 个人成长 | .000 | .000 | ... |
| 工作贡献 | .000 | .000 | ... |
| 人际促进 | .000 | .000 | ... |
| 医疗服务绩效 | .000 | .000 | ... |
| B12 | ... | ... | ... |
| B13 | ... | ... | ... |
| B14 | ... | ... | ... |
| B15 | ... | ... | ... |
| A12 | .000 | ... | ... |
| A11 | .000 | ... | ... |
| A10 | .000 | ... | ... |
| A9 | .000 | ... | ... |
| A8 | .000 | ... | ... |
| A7 | .000 | ... | ... |
| A6 | .000 | ... | ... |
| A5 | .000 | ... | ... |
| A4 | .000 | ... | ... |
| A3 | .000 | ... | ... |
| A2 | .000 | ... | ... |
| A1 | .000 | ... | ... |

## Standardized Indirect Effects (Group number 1 - Default model)

## Standardized Indirect Effects - Lower Bounds (PC) (Group number 1 - Default model)

|  | 性格优势 | 职业召唤 | 工作绩效 |
| --- | --- | --- | --- |
| 职业召唤 | .000 | .000 | .000 |
| 工作绩效 | .230 | .000 | .000 |
| 职业认同 | .549 | .297 | .000 |
| 个人成长 | .571 | .315 | .000 |
| 工作贡献 | .426 | .255 | .000 |
| 人际促进 | .432 | .257 | .000 |
| 医疗服务绩效 | .417 | .252 | .000 |
| B12 | .000 | .000 | .000 |
| B13 | .000 | .000 | .000 |
| B14 | .000 | .000 | .000 |
| B15 | .000 | .000 | .000 |
| A12 | .450 | .000 | .000 |
| A11 | .414 | .000 | .000 |
| A10 | .403 | .000 | .000 |
| A9 | .473 | .000 | .000 |
| A8 | .477 | .000 | .000 |
| A7 | .473 | .000 | .000 |
| A6 | .497 | .000 | .000 |
| A5 | .425 | .000 | .000 |
| A4 | .409 | .000 | .000 |
| A3 | .411 | .000 | .000 |
| A2 | .396 | .000 | .000 |
| A1 | .414 | .000 | .000 |

## Standardized Indirect Effects - Upper Bounds (PC) (Group number 1 - Default model)

|  | 性格优势 | 职业召唤 | 工作绩效 |
| --- | --- | --- | --- |
| 职业召唤 | .000 | .000 | .000 |
| 工作绩效 | .419 | .000 | .000 |
| 职业认同 | .686 | .531 | .000 |
| 个人成长 | .709 | .544 | .000 |
| 工作贡献 | .610 | .434 | .000 |
| 人际促进 | .609 | .436 | .000 |
| 医疗服务绩效 | .592 | .423 | .000 |
| B12 | .000 | .000 | .000 |
| B13 | .000 | .000 | .000 |
| B14 | .000 | .000 | .000 |
| B15 | .000 | .000 | .000 |
| A12 | .615 | .000 | .000 |
| A11 | .573 | .000 | .000 |
| A10 | .592 | .000 | .000 |
| A9 | .645 | .000 | .000 |
| A8 | .650 | .000 | .000 |
| A7 | .641 | .000 | .000 |
| A6 | .670 | .000 | .000 |
| A5 | .592 | .000 | .000 |
| A4 | .588 | .000 | .000 |
| A3 | .576 | .000 | .000 |
| A2 | .566 | .000 | .000 |
| A1 | .585 | .000 | .000 |

## Standardized Indirect Effects - Two Tailed Significance (PC) (Group number 1 - Default model)

|  | 性格优势 | 职业召唤 | 工作绩效 |
| --- | --- | --- | --- |
| 职业召唤 | ... | ... | ... |
| 工作绩效 | .000 | ... | ... |
| 职业认同 | .000 | .000 | ... |
| 个人成长 | .000 | .000 | ... |
| 工作贡献 | .000 | .000 | ... |
| 人际促进 | .000 | .000 | ... |
| 医疗服务绩效 | .000 | .000 | ... |
| B12 | ... | ... | ... |
| B13 | ... | ... | ... |
| B14 | ... | ... | ... |
| B15 | ... | ... | ... |
| A12 | .000 | ... | ... |
| A11 | .000 | ... | ... |
| A10 | .000 | ... | ... |
| A9 | .000 | ... | ... |
| A8 | .000 | ... | ... |
| A7 | .000 | ... | ... |
| A6 | .000 | ... | ... |
| A5 | .000 | ... | ... |
| A4 | .000 | ... | ... |
| A3 | .000 | ... | ... |
| A2 | .000 | ... | ... |
| A1 | .000 | ... | ... |

## Bias-corrected percentile method (Group number 1 - Default model)

## 95% confidence intervals (bias-corrected percentile method)

## Scalar Estimates (Group number 1 - Default model)

## Regression Weights: (Group number 1 - Default model)

| Parameter | | | Estimate | Lower | Upper | P |
| --- | --- | --- | --- | --- | --- | --- |
| 职业召唤 | <--- | 性格优势 | .795 | .664 | .956 | .000 |
| 工作绩效 | <--- | 职业召唤 | .281 | .199 | .379 | .000 |
| 工作绩效 | <--- | 性格优势 | .290 | .177 | .428 | .000 |
| A1 | <--- | 职业召唤 | 1.000 | 1.000 | 1.000 | ... |
| A2 | <--- | 职业召唤 | .945 | .854 | 1.043 | .000 |
| A3 | <--- | 职业召唤 | 1.037 | .931 | 1.157 | .000 |
| A4 | <--- | 职业召唤 | .950 | .851 | 1.061 | .000 |
| A5 | <--- | 职业召唤 | .943 | .802 | 1.088 | .001 |
| A6 | <--- | 职业召唤 | 1.120 | 1.002 | 1.253 | .001 |
| A7 | <--- | 职业召唤 | 1.028 | .908 | 1.177 | .000 |
| A8 | <--- | 职业召唤 | .997 | .899 | 1.110 | .001 |
| A9 | <--- | 职业召唤 | 1.008 | .887 | 1.132 | .001 |
| A10 | <--- | 职业召唤 | .878 | .763 | 1.011 | .000 |
| A11 | <--- | 职业召唤 | 1.114 | .958 | 1.293 | .000 |
| A12 | <--- | 职业召唤 | 1.170 | 1.033 | 1.331 | .000 |
| B15 | <--- | 性格优势 | 1.000 | 1.000 | 1.000 | ... |
| B14 | <--- | 性格优势 | 1.011 | .893 | 1.167 | .000 |
| B13 | <--- | 性格优势 | .976 | .845 | 1.126 | .000 |
| B12 | <--- | 性格优势 | .937 | .815 | 1.076 | .000 |
| 医疗服务绩效 | <--- | 工作绩效 | 1.000 | 1.000 | 1.000 | ... |
| 人际促进 | <--- | 工作绩效 | .988 | .889 | 1.105 | .000 |
| 工作贡献 | <--- | 工作绩效 | 1.303 | 1.143 | 1.505 | .000 |
| 个人成长 | <--- | 工作绩效 | 1.456 | 1.272 | 1.697 | .000 |
| 职业认同 | <--- | 工作绩效 | 1.616 | 1.393 | 1.942 | .000 |

## Standardized Regression Weights: (Group number 1 - Default model)

| Parameter | | | Estimate | Lower | Upper | P |
| --- | --- | --- | --- | --- | --- | --- |
| 职业召唤 | <--- | 性格优势 | .657 | .551 | .744 | .001 |
| 工作绩效 | <--- | 职业召唤 | .484 | .354 | .607 | .001 |
| 工作绩效 | <--- | 性格优势 | .412 | .271 | .547 | .000 |
| A1 | <--- | 职业召唤 | .761 | .677 | .822 | .001 |
| A2 | <--- | 职业召唤 | .736 | .643 | .811 | .000 |
| A3 | <--- | 职业召唤 | .755 | .673 | .823 | .000 |
| A4 | <--- | 职业召唤 | .762 | .704 | .816 | .000 |
| A5 | <--- | 职业召唤 | .777 | .710 | .829 | .001 |
| A6 | <--- | 职业召唤 | .896 | .868 | .919 | .001 |
| A7 | <--- | 职业召唤 | .855 | .808 | .889 | .001 |
| A8 | <--- | 职业召唤 | .864 | .829 | .895 | .001 |
| A9 | <--- | 职业召唤 | .855 | .808 | .891 | .000 |
| A10 | <--- | 职业召唤 | .760 | .685 | .823 | .000 |
| A11 | <--- | 职业召唤 | .753 | .688 | .805 | .001 |
| A12 | <--- | 职业召唤 | .816 | .748 | .863 | .001 |
| B15 | <--- | 性格优势 | .709 | .630 | .772 | .001 |
| B14 | <--- | 性格优势 | .866 | .825 | .900 | .001 |
| B13 | <--- | 性格优势 | .876 | .828 | .911 | .001 |
| B12 | <--- | 性格优势 | .708 | .630 | .775 | .000 |
| 医疗服务绩效 | <--- | 工作绩效 | .695 | .620 | .759 | .000 |
| 人际促进 | <--- | 工作绩效 | .713 | .635 | .777 | .001 |
| 工作贡献 | <--- | 工作绩效 | .713 | .634 | .773 | .001 |
| 个人成长 | <--- | 工作绩效 | .881 | .839 | .915 | .000 |
| 职业认同 | <--- | 工作绩效 | .849 | .798 | .888 | .001 |

## Covariances: (Group number 1 - Default model)

| Parameter | | | Estimate | Lower | Upper | P |
| --- | --- | --- | --- | --- | --- | --- |
| e1 | <--> | e2 | .158 | .084 | .274 | .000 |
| e17 | <--> | e18 | .075 | .055 | .097 | .000 |
| e3 | <--> | e4 | .118 | .058 | .198 | .000 |

## Correlations: (Group number 1 - Default model)

| Parameter | | | Estimate | Lower | Upper | P |
| --- | --- | --- | --- | --- | --- | --- |
| e1 | <--> | e2 | .517 | .332 | .675 | .000 |
| e17 | <--> | e18 | .534 | .427 | .633 | .000 |
| e3 | <--> | e4 | .394 | .213 | .556 | .000 |

## Variances: (Group number 1 - Default model)

| Parameter | | | Estimate | Lower | Upper | P |
| --- | --- | --- | --- | --- | --- | --- |
| 性格优势 |  |  | .282 | .208 | .364 | .000 |
| e22 |  |  | .235 | .164 | .337 | .000 |
| e23 |  |  | .046 | .033 | .066 | .000 |
| e1 |  |  | .300 | .218 | .430 | .000 |
| e2 |  |  | .313 | .218 | .454 | .000 |
| e3 |  |  | .334 | .237 | .472 | .000 |
| e4 |  |  | .269 | .204 | .350 | .000 |
| e5 |  |  | .242 | .195 | .308 | .000 |
| e6 |  |  | .128 | .105 | .157 | .000 |
| e7 |  |  | .161 | .122 | .215 | .000 |
| e8 |  |  | .139 | .111 | .180 | .000 |
| e9 |  |  | .154 | .122 | .192 | .000 |
| e10 |  |  | .234 | .179 | .301 | .000 |
| e11 |  |  | .392 | .310 | .491 | .000 |
| e12 |  |  | .284 | .212 | .389 | .000 |
| e13 |  |  | .278 | .221 | .352 | .000 |
| e14 |  |  | .097 | .073 | .125 | .000 |
| e15 |  |  | .082 | .061 | .108 | .000 |
| e16 |  |  | .246 | .191 | .317 | .000 |
| e17 |  |  | .149 | .126 | .177 | .000 |
| e18 |  |  | .131 | .109 | .157 | .000 |
| e19 |  |  | .228 | .186 | .283 | .000 |
| e20 |  |  | .085 | .064 | .111 | .000 |
| e21 |  |  | .141 | .104 | .186 | .000 |

## Squared Multiple Correlations: (Group number 1 - Default model)

| Parameter | | | Estimate | Lower | Upper | P |
| --- | --- | --- | --- | --- | --- | --- |
| 职业召唤 |  |  | .431 | .304 | .554 | .001 |
| 工作绩效 |  |  | .667 | .558 | .754 | .001 |
| 职业认同 |  |  | .720 | .637 | .788 | .001 |
| 个人成长 |  |  | .777 | .704 | .837 | .000 |
| 工作贡献 |  |  | .509 | .402 | .598 | .001 |
| 人际促进 |  |  | .509 | .403 | .603 | .001 |
| 医疗服务绩效 |  |  | .483 | .385 | .576 | .000 |
| B12 |  |  | .502 | .396 | .601 | .000 |
| B13 |  |  | .767 | .686 | .829 | .001 |
| B14 |  |  | .749 | .681 | .809 | .001 |
| B15 |  |  | .503 | .396 | .597 | .001 |
| A12 |  |  | .666 | .559 | .744 | .001 |
| A11 |  |  | .567 | .473 | .649 | .001 |
| A10 |  |  | .577 | .469 | .678 | .000 |
| A9 |  |  | .731 | .653 | .793 | .000 |
| A8 |  |  | .747 | .687 | .801 | .001 |
| A7 |  |  | .730 | .652 | .791 | .001 |
| A6 |  |  | .802 | .753 | .844 | .001 |
| A5 |  |  | .603 | .504 | .687 | .001 |
| A4 |  |  | .581 | .496 | .666 | .000 |
| A3 |  |  | .571 | .453 | .677 | .000 |
| A2 |  |  | .541 | .413 | .658 | .000 |
| A1 |  |  | .579 | .458 | .676 | .001 |

## Matrices (Group number 1 - Default model)

## Total Effects (Group number 1 - Default model)

## Total Effects - Lower Bounds (BC) (Group number 1 - Default model)

|  | 性格优势 | 职业召唤 | 工作绩效 |
| --- | --- | --- | --- |
| 职业召唤 | .664 | .000 | .000 |
| 工作绩效 | .395 | .199 | .000 |
| 职业认同 | .686 | .308 | 1.393 |
| 个人成长 | .617 | .285 | 1.272 |
| 工作贡献 | .527 | .257 | 1.143 |
| 人际促进 | .394 | .197 | .889 |
| 医疗服务绩效 | .395 | .199 | 1.000 |
| B12 | .815 | .000 | .000 |
| B13 | .845 | .000 | .000 |
| B14 | .893 | .000 | .000 |
| B15 | 1.000 | .000 | .000 |
| A12 | .793 | 1.033 | .000 |
| A11 | .743 | .958 | .000 |
| A10 | .562 | .763 | .000 |
| A9 | .671 | .887 | .000 |
| A8 | .668 | .899 | .000 |
| A7 | .687 | .908 | .000 |
| A6 | .754 | 1.002 | .000 |
| A5 | .612 | .802 | .000 |
| A4 | .614 | .851 | .000 |
| A3 | .693 | .931 | .000 |
| A2 | .628 | .854 | .000 |
| A1 | .664 | 1.000 | .000 |

## Total Effects - Upper Bounds (BC) (Group number 1 - Default model)

|  | 性格优势 | 职业召唤 | 工作绩效 |
| --- | --- | --- | --- |
| 职业召唤 | .956 | .000 | .000 |
| 工作绩效 | .649 | .379 | .000 |
| 职业认同 | .995 | .628 | 1.942 |
| 个人成长 | .908 | .558 | 1.697 |
| 工作贡献 | .830 | .500 | 1.505 |
| 人际促进 | .635 | .373 | 1.105 |
| 医疗服务绩效 | .649 | .379 | 1.000 |
| B12 | 1.076 | .000 | .000 |
| B13 | 1.126 | .000 | .000 |
| B14 | 1.167 | .000 | .000 |
| B15 | 1.000 | .000 | .000 |
| A12 | 1.086 | 1.331 | .000 |
| A11 | 1.046 | 1.293 | .000 |
| A10 | .839 | 1.011 | .000 |
| A9 | .954 | 1.132 | .000 |
| A8 | .940 | 1.110 | .000 |
| A7 | .966 | 1.177 | .000 |
| A6 | 1.038 | 1.253 | .000 |
| A5 | .901 | 1.088 | .000 |
| A4 | .917 | 1.061 | .000 |
| A3 | .981 | 1.157 | .000 |
| A2 | .894 | 1.043 | .000 |
| A1 | .956 | 1.000 | .000 |

## Total Effects - Two Tailed Significance (BC) (Group number 1 - Default model)

|  | 性格优势 | 职业召唤 | 工作绩效 |
| --- | --- | --- | --- |
| 职业召唤 | .000 | ... | ... |
| 工作绩效 | .000 | .000 | ... |
| 职业认同 | .000 | .000 | .000 |
| 个人成长 | .000 | .000 | .000 |
| 工作贡献 | .000 | .000 | .000 |
| 人际促进 | .000 | .000 | .000 |
| 医疗服务绩效 | .000 | .000 | ... |
| B12 | .000 | ... | ... |
| B13 | .000 | ... | ... |
| B14 | .000 | ... | ... |
| B15 | ... | ... | ... |
| A12 | .000 | .000 | ... |
| A11 | .000 | .000 | ... |
| A10 | .000 | .000 | ... |
| A9 | .000 | .001 | ... |
| A8 | .000 | .001 | ... |
| A7 | .000 | .000 | ... |
| A6 | .000 | .001 | ... |
| A5 | .000 | .001 | ... |
| A4 | .000 | .000 | ... |
| A3 | .000 | .000 | ... |
| A2 | .000 | .000 | ... |
| A1 | .000 | ... | ... |

## Standardized Total Effects (Group number 1 - Default model)

## Standardized Total Effects - Lower Bounds (BC) (Group number 1 - Default model)

|  | 性格优势 | 职业召唤 | 工作绩效 |
| --- | --- | --- | --- |
| 职业召唤 | .551 | .000 | .000 |
| 工作绩效 | .643 | .354 | .000 |
| 职业认同 | .547 | .296 | .798 |
| 个人成长 | .570 | .313 | .839 |
| 工作贡献 | .424 | .251 | .634 |
| 人际促进 | .431 | .256 | .635 |
| 医疗服务绩效 | .414 | .251 | .620 |
| B12 | .630 | .000 | .000 |
| B13 | .828 | .000 | .000 |
| B14 | .825 | .000 | .000 |
| B15 | .630 | .000 | .000 |
| A12 | .450 | .748 | .000 |
| A11 | .412 | .688 | .000 |
| A10 | .402 | .685 | .000 |
| A9 | .472 | .808 | .000 |
| A8 | .474 | .829 | .000 |
| A7 | .471 | .808 | .000 |
| A6 | .492 | .868 | .000 |
| A5 | .423 | .710 | .000 |
| A4 | .407 | .704 | .000 |
| A3 | .413 | .673 | .000 |
| A2 | .398 | .643 | .000 |
| A1 | .414 | .677 | .000 |

## Standardized Total Effects - Upper Bounds (BC) (Group number 1 - Default model)

|  | 性格优势 | 职业召唤 | 工作绩效 |
| --- | --- | --- | --- |
| 职业召唤 | .744 | .000 | .000 |
| 工作绩效 | .805 | .607 | .000 |
| 职业认同 | .684 | .529 | .888 |
| 个人成长 | .709 | .542 | .915 |
| 工作贡献 | .609 | .431 | .773 |
| 人际促进 | .608 | .434 | .777 |
| 医疗服务绩效 | .589 | .422 | .759 |
| B12 | .775 | .000 | .000 |
| B13 | .911 | .000 | .000 |
| B14 | .900 | .000 | .000 |
| B15 | .772 | .000 | .000 |
| A12 | .614 | .863 | .000 |
| A11 | .572 | .805 | .000 |
| A10 | .590 | .823 | .000 |
| A9 | .644 | .891 | .000 |
| A8 | .648 | .895 | .000 |
| A7 | .640 | .889 | .000 |
| A6 | .666 | .919 | .000 |
| A5 | .591 | .829 | .000 |
| A4 | .587 | .816 | .000 |
| A3 | .577 | .823 | .000 |
| A2 | .568 | .811 | .000 |
| A1 | .585 | .822 | .000 |

## Standardized Total Effects - Two Tailed Significance (BC) (Group number 1 - Default model)

|  | 性格优势 | 职业召唤 | 工作绩效 |
| --- | --- | --- | --- |
| 职业召唤 | .001 | ... | ... |
| 工作绩效 | .000 | .001 | ... |
| 职业认同 | .000 | .000 | .001 |
| 个人成长 | .000 | .000 | .000 |
| 工作贡献 | .000 | .000 | .001 |
| 人际促进 | .000 | .000 | .001 |
| 医疗服务绩效 | .001 | .000 | .000 |
| B12 | .000 | ... | ... |
| B13 | .001 | ... | ... |
| B14 | .001 | ... | ... |
| B15 | .001 | ... | ... |
| A12 | .000 | .001 | ... |
| A11 | .000 | .001 | ... |
| A10 | .000 | .000 | ... |
| A9 | .000 | .000 | ... |
| A8 | .001 | .001 | ... |
| A7 | .000 | .001 | ... |
| A6 | .001 | .001 | ... |
| A5 | .000 | .001 | ... |
| A4 | .000 | .000 | ... |
| A3 | .000 | .000 | ... |
| A2 | .000 | .000 | ... |
| A1 | .000 | .001 | ... |

## Direct Effects (Group number 1 - Default model)

## Direct Effects - Lower Bounds (BC) (Group number 1 - Default model)

|  | 性格优势 | 职业召唤 | 工作绩效 |
| --- | --- | --- | --- |
| 职业召唤 | .664 | .000 | .000 |
| 工作绩效 | .177 | .199 | .000 |
| 职业认同 | .000 | .000 | 1.393 |
| 个人成长 | .000 | .000 | 1.272 |
| 工作贡献 | .000 | .000 | 1.143 |
| 人际促进 | .000 | .000 | .889 |
| 医疗服务绩效 | .000 | .000 | 1.000 |
| B12 | .815 | .000 | .000 |
| B13 | .845 | .000 | .000 |
| B14 | .893 | .000 | .000 |
| B15 | 1.000 | .000 | .000 |
| A12 | .000 | 1.033 | .000 |
| A11 | .000 | .958 | .000 |
| A10 | .000 | .763 | .000 |
| A9 | .000 | .887 | .000 |
| A8 | .000 | .899 | .000 |
| A7 | .000 | .908 | .000 |
| A6 | .000 | 1.002 | .000 |
| A5 | .000 | .802 | .000 |
| A4 | .000 | .851 | .000 |
| A3 | .000 | .931 | .000 |
| A2 | .000 | .854 | .000 |
| A1 | .000 | 1.000 | .000 |

## Direct Effects - Upper Bounds (BC) (Group number 1 - Default model)

|  | 性格优势 | 职业召唤 | 工作绩效 |
| --- | --- | --- | --- |
| 职业召唤 | .956 | .000 | .000 |
| 工作绩效 | .428 | .379 | .000 |
| 职业认同 | .000 | .000 | 1.942 |
| 个人成长 | .000 | .000 | 1.697 |
| 工作贡献 | .000 | .000 | 1.505 |
| 人际促进 | .000 | .000 | 1.105 |
| 医疗服务绩效 | .000 | .000 | 1.000 |
| B12 | 1.076 | .000 | .000 |
| B13 | 1.126 | .000 | .000 |
| B14 | 1.167 | .000 | .000 |
| B15 | 1.000 | .000 | .000 |
| A12 | .000 | 1.331 | .000 |
| A11 | .000 | 1.293 | .000 |
| A10 | .000 | 1.011 | .000 |
| A9 | .000 | 1.132 | .000 |
| A8 | .000 | 1.110 | .000 |
| A7 | .000 | 1.177 | .000 |
| A6 | .000 | 1.253 | .000 |
| A5 | .000 | 1.088 | .000 |
| A4 | .000 | 1.061 | .000 |
| A3 | .000 | 1.157 | .000 |
| A2 | .000 | 1.043 | .000 |
| A1 | .000 | 1.000 | .000 |

## Direct Effects - Two Tailed Significance (BC) (Group number 1 - Default model)

|  | 性格优势 | 职业召唤 | 工作绩效 |
| --- | --- | --- | --- |
| 职业召唤 | .000 | ... | ... |
| 工作绩效 | .000 | .000 | ... |
| 职业认同 | ... | ... | .000 |
| 个人成长 | ... | ... | .000 |
| 工作贡献 | ... | ... | .000 |
| 人际促进 | ... | ... | .000 |
| 医疗服务绩效 | ... | ... | ... |
| B12 | .000 | ... | ... |
| B13 | .000 | ... | ... |
| B14 | .000 | ... | ... |
| B15 | ... | ... | ... |
| A12 | ... | .000 | ... |
| A11 | ... | .000 | ... |
| A10 | ... | .000 | ... |
| A9 | ... | .001 | ... |
| A8 | ... | .001 | ... |
| A7 | ... | .000 | ... |
| A6 | ... | .001 | ... |
| A5 | ... | .001 | ... |
| A4 | ... | .000 | ... |
| A3 | ... | .000 | ... |
| A2 | ... | .000 | ... |
| A1 | ... | ... | ... |

## Standardized Direct Effects (Group number 1 - Default model)

## Standardized Direct Effects - Lower Bounds (BC) (Group number 1 - Default model)

|  | 性格优势 | 职业召唤 | 工作绩效 |
| --- | --- | --- | --- |
| 职业召唤 | .551 | .000 | .000 |
| 工作绩效 | .271 | .354 | .000 |
| 职业认同 | .000 | .000 | .798 |
| 个人成长 | .000 | .000 | .839 |
| 工作贡献 | .000 | .000 | .634 |
| 人际促进 | .000 | .000 | .635 |
| 医疗服务绩效 | .000 | .000 | .620 |
| B12 | .630 | .000 | .000 |
| B13 | .828 | .000 | .000 |
| B14 | .825 | .000 | .000 |
| B15 | .630 | .000 | .000 |
| A12 | .000 | .748 | .000 |
| A11 | .000 | .688 | .000 |
| A10 | .000 | .685 | .000 |
| A9 | .000 | .808 | .000 |
| A8 | .000 | .829 | .000 |
| A7 | .000 | .808 | .000 |
| A6 | .000 | .868 | .000 |
| A5 | .000 | .710 | .000 |
| A4 | .000 | .704 | .000 |
| A3 | .000 | .673 | .000 |
| A2 | .000 | .643 | .000 |
| A1 | .000 | .677 | .000 |

## Standardized Direct Effects - Upper Bounds (BC) (Group number 1 - Default model)

|  | 性格优势 | 职业召唤 | 工作绩效 |
| --- | --- | --- | --- |
| 职业召唤 | .744 | .000 | .000 |
| 工作绩效 | .547 | .607 | .000 |
| 职业认同 | .000 | .000 | .888 |
| 个人成长 | .000 | .000 | .915 |
| 工作贡献 | .000 | .000 | .773 |
| 人际促进 | .000 | .000 | .777 |
| 医疗服务绩效 | .000 | .000 | .759 |
| B12 | .775 | .000 | .000 |
| B13 | .911 | .000 | .000 |
| B14 | .900 | .000 | .000 |
| B15 | .772 | .000 | .000 |
| A12 | .000 | .863 | .000 |
| A11 | .000 | .805 | .000 |
| A10 | .000 | .823 | .000 |
| A9 | .000 | .891 | .000 |
| A8 | .000 | .895 | .000 |
| A7 | .000 | .889 | .000 |
| A6 | .000 | .919 | .000 |
| A5 | .000 | .829 | .000 |
| A4 | .000 | .816 | .000 |
| A3 | .000 | .823 | .000 |
| A2 | .000 | .811 | .000 |
| A1 | .000 | .822 | .000 |

## Standardized Direct Effects - Two Tailed Significance (BC) (Group number 1 - Default model)

|  | 性格优势 | 职业召唤 | 工作绩效 |
| --- | --- | --- | --- |
| 职业召唤 | .001 | ... | ... |
| 工作绩效 | .000 | .001 | ... |
| 职业认同 | ... | ... | .001 |
| 个人成长 | ... | ... | .000 |
| 工作贡献 | ... | ... | .001 |
| 人际促进 | ... | ... | .001 |
| 医疗服务绩效 | ... | ... | .000 |
| B12 | .000 | ... | ... |
| B13 | .001 | ... | ... |
| B14 | .001 | ... | ... |
| B15 | .001 | ... | ... |
| A12 | ... | .001 | ... |
| A11 | ... | .001 | ... |
| A10 | ... | .000 | ... |
| A9 | ... | .000 | ... |
| A8 | ... | .001 | ... |
| A7 | ... | .001 | ... |
| A6 | ... | .001 | ... |
| A5 | ... | .001 | ... |
| A4 | ... | .000 | ... |
| A3 | ... | .000 | ... |
| A2 | ... | .000 | ... |
| A1 | ... | .001 | ... |

## Indirect Effects (Group number 1 - Default model)

## Indirect Effects - Lower Bounds (BC) (Group number 1 - Default model)

|  | 性格优势 | 职业召唤 | 工作绩效 |
| --- | --- | --- | --- |
| 职业召唤 | .000 | .000 | .000 |
| 工作绩效 | .160 | .000 | .000 |
| 职业认同 | .686 | .308 | .000 |
| 个人成长 | .617 | .285 | .000 |
| 工作贡献 | .527 | .257 | .000 |
| 人际促进 | .394 | .197 | .000 |
| 医疗服务绩效 | .395 | .199 | .000 |
| B12 | .000 | .000 | .000 |
| B13 | .000 | .000 | .000 |
| B14 | .000 | .000 | .000 |
| B15 | .000 | .000 | .000 |
| A12 | .793 | .000 | .000 |
| A11 | .743 | .000 | .000 |
| A10 | .562 | .000 | .000 |
| A9 | .671 | .000 | .000 |
| A8 | .668 | .000 | .000 |
| A7 | .687 | .000 | .000 |
| A6 | .754 | .000 | .000 |
| A5 | .612 | .000 | .000 |
| A4 | .614 | .000 | .000 |
| A3 | .693 | .000 | .000 |
| A2 | .628 | .000 | .000 |
| A1 | .664 | .000 | .000 |

## Indirect Effects - Upper Bounds (BC) (Group number 1 - Default model)

|  | 性格优势 | 职业召唤 | 工作绩效 |
| --- | --- | --- | --- |
| 职业召唤 | .000 | .000 | .000 |
| 工作绩效 | .306 | .000 | .000 |
| 职业认同 | .995 | .628 | .000 |
| 个人成长 | .908 | .558 | .000 |
| 工作贡献 | .830 | .500 | .000 |
| 人际促进 | .635 | .373 | .000 |
| 医疗服务绩效 | .649 | .379 | .000 |
| B12 | .000 | .000 | .000 |
| B13 | .000 | .000 | .000 |
| B14 | .000 | .000 | .000 |
| B15 | .000 | .000 | .000 |
| A12 | 1.086 | .000 | .000 |
| A11 | 1.046 | .000 | .000 |
| A10 | .839 | .000 | .000 |
| A9 | .954 | .000 | .000 |
| A8 | .940 | .000 | .000 |
| A7 | .966 | .000 | .000 |
| A6 | 1.038 | .000 | .000 |
| A5 | .901 | .000 | .000 |
| A4 | .917 | .000 | .000 |
| A3 | .981 | .000 | .000 |
| A2 | .894 | .000 | .000 |
| A1 | .956 | .000 | .000 |

## Indirect Effects - Two Tailed Significance (BC) (Group number 1 - Default model)

|  | 性格优势 | 职业召唤 | 工作绩效 |
| --- | --- | --- | --- |
| 职业召唤 | ... | ... | ... |
| 工作绩效 | .000 | ... | ... |
| 职业认同 | .000 | .000 | ... |
| 个人成长 | .000 | .000 | ... |
| 工作贡献 | .000 | .000 | ... |
| 人际促进 | .000 | .000 | ... |
| 医疗服务绩效 | .000 | .000 | ... |
| B12 | ... | ... | ... |
| B13 | ... | ... | ... |
| B14 | ... | ... | ... |
| B15 | ... | ... | ... |
| A12 | .000 | ... | ... |
| A11 | .000 | ... | ... |
| A10 | .000 | ... | ... |
| A9 | .000 | ... | ... |
| A8 | .000 | ... | ... |
| A7 | .000 | ... | ... |
| A6 | .000 | ... | ... |
| A5 | .000 | ... | ... |
| A4 | .000 | ... | ... |
| A3 | .000 | ... | ... |
| A2 | .000 | ... | ... |
| A1 | .000 | ... | ... |

## Standardized Indirect Effects (Group number 1 - Default model)

## Standardized Indirect Effects - Lower Bounds (BC) (Group number 1 - Default model)

|  | 性格优势 | 职业召唤 | 工作绩效 |
| --- | --- | --- | --- |
| 职业召唤 | .000 | .000 | .000 |
| 工作绩效 | .230 | .000 | .000 |
| 职业认同 | .547 | .296 | .000 |
| 个人成长 | .570 | .313 | .000 |
| 工作贡献 | .424 | .251 | .000 |
| 人际促进 | .431 | .256 | .000 |
| 医疗服务绩效 | .414 | .251 | .000 |
| B12 | .000 | .000 | .000 |
| B13 | .000 | .000 | .000 |
| B14 | .000 | .000 | .000 |
| B15 | .000 | .000 | .000 |
| A12 | .450 | .000 | .000 |
| A11 | .412 | .000 | .000 |
| A10 | .402 | .000 | .000 |
| A9 | .472 | .000 | .000 |
| A8 | .474 | .000 | .000 |
| A7 | .471 | .000 | .000 |
| A6 | .492 | .000 | .000 |
| A5 | .423 | .000 | .000 |
| A4 | .407 | .000 | .000 |
| A3 | .413 | .000 | .000 |
| A2 | .398 | .000 | .000 |
| A1 | .414 | .000 | .000 |

## Standardized Indirect Effects - Upper Bounds (BC) (Group number 1 - Default model)

|  | 性格优势 | 职业召唤 | 工作绩效 |
| --- | --- | --- | --- |
| 职业召唤 | .000 | .000 | .000 |
| 工作绩效 | .419 | .000 | .000 |
| 职业认同 | .684 | .529 | .000 |
| 个人成长 | .709 | .542 | .000 |
| 工作贡献 | .609 | .431 | .000 |
| 人际促进 | .608 | .434 | .000 |
| 医疗服务绩效 | .589 | .422 | .000 |
| B12 | .000 | .000 | .000 |
| B13 | .000 | .000 | .000 |
| B14 | .000 | .000 | .000 |
| B15 | .000 | .000 | .000 |
| A12 | .614 | .000 | .000 |
| A11 | .572 | .000 | .000 |
| A10 | .590 | .000 | .000 |
| A9 | .644 | .000 | .000 |
| A8 | .648 | .000 | .000 |
| A7 | .640 | .000 | .000 |
| A6 | .666 | .000 | .000 |
| A5 | .591 | .000 | .000 |
| A4 | .587 | .000 | .000 |
| A3 | .577 | .000 | .000 |
| A2 | .568 | .000 | .000 |
| A1 | .585 | .000 | .000 |

## Standardized Indirect Effects - Two Tailed Significance (BC) (Group number 1 - Default model)

|  | 性格优势 | 职业召唤 | 工作绩效 |
| --- | --- | --- | --- |
| 职业召唤 | ... | ... | ... |
| 工作绩效 | .000 | ... | ... |
| 职业认同 | .000 | .000 | ... |
| 个人成长 | .000 | .000 | ... |
| 工作贡献 | .000 | .000 | ... |
| 人际促进 | .000 | .000 | ... |
| 医疗服务绩效 | .001 | .000 | ... |
| B12 | ... | ... | ... |
| B13 | ... | ... | ... |
| B14 | ... | ... | ... |
| B15 | ... | ... | ... |
| A12 | .000 | ... | ... |
| A11 | .000 | ... | ... |
| A10 | .000 | ... | ... |
| A9 | .000 | ... | ... |
| A8 | .001 | ... | ... |
| A7 | .000 | ... | ... |
| A6 | .001 | ... | ... |
| A5 | .000 | ... | ... |
| A4 | .000 | ... | ... |
| A3 | .000 | ... | ... |
| A2 | .000 | ... | ... |
| A1 | .000 | ... | ... |

## Minimization History (Default model)

| Iteration |  | Negative eigenvalues | Condition # | Smallest eigenvalue | Diameter | F | NTries | Ratio |
| --- | --- | --- | --- | --- | --- | --- | --- | --- |
| 0 | e | 10 |  | -2.066 | 9999.000 | 7235.423 | 0 | 9999.000 |
| 1 | e | 15 |  | -.386 | 2.296 | 4461.130 | 18 | .349 |
| 2 | e\* | 6 |  | -.366 | 1.556 | 2524.043 | 5 | .926 |
| 3 | e | 3 |  | -.056 | .835 | 1711.815 | 4 | .874 |
| 4 | e | 1 |  | -.007 | .645 | 1453.941 | 5 | .531 |
| 5 | e | 0 | 1249.838 |  | .622 | 1142.920 | 5 | .959 |
| 6 | e | 0 | 320.095 |  | .714 | 1068.227 | 3 | .000 |
| 7 | e | 0 | 207.386 |  | .626 | 938.641 | 1 | 1.130 |
| 8 | e | 0 | 185.828 |  | .257 | 918.343 | 1 | 1.110 |
| 9 | e | 0 | 182.257 |  | .070 | 917.424 | 1 | 1.050 |
| 10 | e | 0 | 173.228 |  | .006 | 917.418 | 1 | 1.006 |
| 11 | e | 0 | 174.092 |  | .000 | 917.418 | 1 | 1.000 |

## Bootstrap (Default model)

## Summary of Bootstrap Iterations (Default model)

## (Default model)

| Iterations | Method 0 | Method 1 | Method 2 |
| --- | --- | --- | --- |
| 1 | 0 | 0 | 0 |
| 2 | 0 | 0 | 0 |
| 3 | 0 | 0 | 0 |
| 4 | 0 | 0 | 0 |
| 5 | 0 | 0 | 0 |
| 6 | 0 | 0 | 0 |
| 7 | 0 | 19 | 0 |
| 8 | 0 | 451 | 0 |
| 9 | 0 | 1470 | 0 |
| 10 | 0 | 1532 | 0 |
| 11 | 0 | 948 | 0 |
| 12 | 0 | 386 | 0 |
| 13 | 0 | 144 | 0 |
| 14 | 0 | 37 | 0 |
| 15 | 0 | 12 | 0 |
| 16 | 0 | 1 | 0 |
| 17 | 0 | 0 | 0 |
| 18 | 0 | 0 | 0 |
| 19 | 0 | 0 | 0 |
| Total | 0 | 5000 | 0 |

0 bootstrap samples were unused because of a singular covariance matrix.

0 bootstrap samples were unused because a solution was not found.

5000 usable bootstrap samples were obtained.

## Bootstrap Distributions (Default model)

## ML discrepancy (implied vs sample) (Default model)

|  |  |  |
| --- | --- | --- |
|  |  | |-------------------- |
|  | 807.016 | |\* |
|  | 881.142 | |\* |
|  | 955.268 | |\*\*\* |
|  | 1029.394 | |\*\*\*\*\*\*\*\*\*\* |
|  | 1103.520 | |\*\*\*\*\*\*\*\*\*\*\*\*\*\*\*\*\* |
|  | 1177.645 | |\*\*\*\*\*\*\*\*\*\*\*\*\*\*\*\*\*\*\*\* |
|  | 1251.771 | |\*\*\*\*\*\*\*\*\*\*\*\*\*\*\*\*\* |
| N = 5000 | 1325.897 | |\*\*\*\*\*\*\*\*\*\*\*\* |
| Mean = 1207.011 | 1400.023 | |\*\*\*\*\*\*\* |
| S. e. = 1.943 | 1474.149 | |\*\*\* |
|  | 1548.274 | |\*\* |
|  | 1622.400 | |\* |
|  | 1696.526 | |\* |
|  | 1770.652 | |\* |
|  | 1844.777 | |\* |
|  |  | |-------------------- |

## ML discrepancy (implied vs pop) (Default model)

|  |  |  |
| --- | --- | --- |
|  |  | |-------------------- |
|  | 958.985 | |\* |
|  | 983.769 | |\*\*\*\*\* |
|  | 1008.553 | |\*\*\*\*\*\*\*\*\*\*\*\*\*\*\*\*\* |
|  | 1033.337 | |\*\*\*\*\*\*\*\*\*\*\*\*\*\*\*\*\*\*\*\* |
|  | 1058.121 | |\*\*\*\*\*\*\*\*\*\*\*\*\*\*\* |
|  | 1082.905 | |\*\*\*\*\*\*\*\*\* |
|  | 1107.689 | |\*\*\*\*\* |
| N = 5000 | 1132.472 | |\*\* |
| Mean = 1046.103 | 1157.256 | |\* |
| S. e. = .571 | 1182.040 | |\* |
|  | 1206.824 | |\* |
|  | 1231.608 | |\* |
|  | 1256.392 | |\* |
|  | 1281.176 | |\* |
|  | 1305.959 | |\* |
|  |  | |-------------------- |

## K-L overoptimism (unstabilized) (Default model)

|  |  |  |
| --- | --- | --- |
|  |  | |-------------------- |
|  | -1281.772 | |\* |
|  | -1037.211 | |\* |
|  | -792.650 | |\*\* |
|  | -548.089 | |\*\*\*\*\*\* |
|  | -303.528 | |\*\*\*\*\*\*\*\*\*\*\* |
|  | -58.967 | |\*\*\*\*\*\*\*\*\*\*\*\*\*\*\*\*\* |
|  | 185.594 | |\*\*\*\*\*\*\*\*\*\*\*\*\*\*\*\*\*\*\*\* |
| N = 5000 | 430.155 | |\*\*\*\*\*\*\*\*\*\*\*\*\*\*\*\*\*\* |
| Mean = 247.173 | 674.716 | |\*\*\*\*\*\*\*\*\*\*\*\*\* |
| S. e. = 6.477 | 919.277 | |\*\*\*\*\*\*\*\* |
|  | 1163.838 | |\*\*\* |
|  | 1408.399 | |\* |
|  | 1652.960 | |\* |
|  | 1897.521 | |\* |
|  | 2142.082 | |\* |
|  |  | |-------------------- |

## K-L overoptimism (stabilized) (Default model)

|  |  |  |
| --- | --- | --- |
|  |  | |-------------------- |
|  | -236.315 | |\* |
|  | -152.944 | |\* |
|  | -69.573 | |\*\* |
|  | 13.798 | |\*\*\*\*\*\* |
|  | 97.169 | |\*\*\*\*\*\*\*\*\*\*\*\*\* |
|  | 180.540 | |\*\*\*\*\*\*\*\*\*\*\*\*\*\*\*\*\*\*\*\* |
|  | 263.911 | |\*\*\*\*\*\*\*\*\*\*\*\*\*\*\*\*\*\*\*\* |
| N = 5000 | 347.282 | |\*\*\*\*\*\*\*\*\*\*\*\*\*\*\* |
| Mean = 252.870 | 430.653 | |\*\*\*\*\*\*\*\*\* |
| S. e. = 2.165 | 514.024 | |\*\*\*\*\* |
|  | 597.395 | |\*\* |
|  | 680.766 | |\* |
|  | 764.137 | |\* |
|  | 847.508 | |\* |
|  | 930.879 | |\* |
|  |  | |-------------------- |

## Model Fit Summary

## CMIN

| Model | NPAR | CMIN | DF | P | CMIN/DF |
| --- | --- | --- | --- | --- | --- |
| Default model | 48 | 917.418 | 183 | .000 | 5.013 |
| Saturated model | 231 | .000 | 0 |
| Independence model | 21 | 7645.318 | 210 | .000 | 36.406 |

## RMR, GFI

| Model | RMR | GFI | AGFI | PGFI |
| --- | --- | --- | --- | --- |
| Default model | .032 | .809 | .759 | .641 |
| Saturated model | .000 | 1.000 |  |  |
| Independence model | .299 | .146 | .061 | .133 |

## Baseline Comparisons

| Model | NFI Delta1 | RFI rho1 | IFI Delta2 | TLI rho2 | CFI |
| --- | --- | --- | --- | --- | --- |
| Default model | .880 | .862 | .902 | .887 | .901 |
| Saturated model | 1.000 |  | 1.000 |  | 1.000 |
| Independence model | .000 | .000 | .000 | .000 | .000 |

## Parsimony-Adjusted Measures

| Model | PRATIO | PNFI | PCFI |
| --- | --- | --- | --- |
| Default model | .871 | .767 | .785 |
| Saturated model | .000 | .000 | .000 |
| Independence model | 1.000 | .000 | .000 |

## NCP

| Model | NCP | LO 90 | HI 90 |
| --- | --- | --- | --- |
| Default model | 734.418 | 643.597 | 832.754 |
| Saturated model | .000 | .000 | .000 |
| Independence model | 7435.318 | 7152.835 | 7724.138 |

## FMIN

| Model | FMIN | F0 | LO 90 | HI 90 |
| --- | --- | --- | --- | --- |
| Default model | 2.221 | 1.778 | 1.558 | 2.016 |
| Saturated model | .000 | .000 | .000 | .000 |
| Independence model | 18.512 | 18.003 | 17.319 | 18.703 |

## RMSEA

| Model | RMSEA | LO 90 | HI 90 | PCLOSE |
| --- | --- | --- | --- | --- |
| Default model | .099 | .092 | .105 | .000 |
| Independence model | .293 | .287 | .298 | .000 |

## AIC

| Model | AIC | BCC | BIC | CAIC |
| --- | --- | --- | --- | --- |
| Default model | 1013.418 | 1018.820 | 1206.660 | 1254.660 |
| Saturated model | 462.000 | 487.995 | 1391.975 | 1622.975 |
| Independence model | 7687.318 | 7689.681 | 7771.861 | 7792.861 |

## ECVI

| Model | ECVI | LO 90 | HI 90 | MECVI |
| --- | --- | --- | --- | --- |
| Default model | 2.454 | 2.234 | 2.692 | 2.467 |
| Saturated model | 1.119 | 1.119 | 1.119 | 1.182 |
| Independence model | 18.613 | 17.929 | 19.313 | 18.619 |

## HOELTER

| Model | HOELTER .05 | HOELTER .01 |
| --- | --- | --- |
| Default model | 98 | 104 |
| Independence model | 14 | 15 |

## Execution time summary

|  |  |
| --- | --- |
| Minimization: | .015 |
| Miscellaneous: | .282 |
| Bootstrap: | 2.619 |
| Total: | 2.916 |
