# Supplementary material for: The impact of medical staff’s character strengths on job performance in Hangzhou hospitals
Source: Front Psychol. 2023 Nov 23;14:1291851. doi: 10.3389/fpsyg.2023.1291851 (PMC10701392; doi:10.3389/fpsyg.2023.1291851)
Supplement: Supplementary file 1 [file Data_Sheet_1.ZIP › statistical analysis process/AMOS analysis model outputresults/╨▐╒2║≤─ú╨═╡≈╣2╕±╩╜╣ñ╫≈╝¿╨o╬σ╕÷╬1⁄4╢╚╕─│╔╙ó╬─.AmosOutput]

修正后模型调过格式工作绩效五个维度改成英文.amw


# F:\U盘备份\黄老师\第一篇\2023.8.2再次核对修改\8.9发给黄老师审阅\统计分析过程\AMOS分析模型输出结果\修正后模型调过格式工作绩效五个维度改成英文.amw

## Analysis Summary

## Date and Time

Date: 2023年8月9日

Time: 15:34:41

## Title

修正后模型调过格式工作绩效五个维度改成英文: 2023年8月9日 15:34

## Groups

## Group number 1 (Group number 1)

## Notes for Group (Group number 1)

The model is recursive.

Sample size = 414

## Variable Summary (Group number 1)

## Your model contains the following variables (Group number 1)

Observed, endogenous variables

A1我热爱我现在的工作

A2与其它工作相比，我还是更喜欢现在的工作

A3我对我目前的职业感到非常满足

A4我愿意为现在的工作而作出一些牺牲

A5自我介绍时，我首先想起我的职业

A6即便遇到阻碍，我仍然会选择继续现在的工作

A7我觉得现在的工作是我生命中的一部分

A8我的职业让我充满使命感

A9我的职业一直存在于我的内心深处

A10我会经常思考工作相关的事

A11如果放弃现在的工作，我会觉得失去了意义

A12我目前的职业可以为我带来快乐

B15我总能乐观地发现它积极的一面

B14我公平地对待所有人，不管他们是什么身份

B13当他人遇到困难时，我愿意伸出援手

B12同事和病人都认为我性格沉稳

Medicalserviceperformance

Interpersonalpromotion

Workcontribution

Personalgrowth

Professionalidentity

Unobserved, endogenous variables

Career\_calling

Job\_performance

Unobserved, exogenous variables

e1

e2

e3

e4

e5

e6

e7

e8

e9

e10

e11

e12

Character\_strength

e13

e14

e15

e16

e17

e18

e19

e20

e21

e22

e23

## Variable counts (Group number 1)

|  |  |
| --- | --- |
| Number of variables in your model: | 47 |
| Number of observed variables: | 21 |
| Number of unobserved variables: | 26 |
| Number of exogenous variables: | 24 |
| Number of endogenous variables: | 23 |

## Parameter Summary (Group number 1)

|  | Weights | Covariances | Variances | Means | Intercepts | Total |
| --- | --- | --- | --- | --- | --- | --- |
| Fixed | 26 | 0 | 0 | 0 | 0 | 26 |
| Labeled | 0 | 0 | 0 | 0 | 0 | 0 |
| Unlabeled | 21 | 12 | 24 | 0 | 0 | 57 |
| Total | 47 | 12 | 24 | 0 | 0 | 83 |

## Models

## Default model (Default model)

## Notes for Model (Default model)

## Computation of degrees of freedom (Default model)

|  |  |
| --- | --- |
| Number of distinct sample moments: | 231 |
| Number of distinct parameters to be estimated: | 57 |
| Degrees of freedom (231 - 57): | 174 |

## Result (Default model)

Minimum was achieved

Chi-square = 627.675

Degrees of freedom = 174

Probability level = .000

## Group number 1 (Group number 1 - Default model)

## Estimates (Group number 1 - Default model)

## Scalar Estimates (Group number 1 - Default model)

## Maximum Likelihood Estimates

## Regression Weights: (Group number 1 - Default model)

|  |  |  | Estimate | S.E. | C.R. | P | Label |
| --- | --- | --- | --- | --- | --- | --- | --- |
| Career\_calling | <--- | Character\_strength | .801 | .075 | 10.687 | \*\*\* |  |
| Job\_performance | <--- | Career\_calling | .265 | .036 | 7.374 | \*\*\* |  |
| Job\_performance | <--- | Character\_strength | .382 | .048 | 7.934 | \*\*\* |  |
| A6即便遇到阻碍，我仍然会选择继续现在的工作 | <--- | Career\_calling | 1.151 | .057 | 20.024 | \*\*\* |  |
| A7我觉得现在的工作是我生命中的一部分 | <--- | Career\_calling | 1.047 | .056 | 18.745 | \*\*\* |  |
| A8我的职业让我充满使命感 | <--- | Career\_calling | 1.020 | .053 | 19.108 | \*\*\* |  |
| B15我总能乐观地发现它积极的一面 | <--- | Character\_strength | 1.000 |  |
| B14我公平地对待所有人，不管他们是什么身份 | <--- | Character\_strength | 1.044 | .066 | 15.727 | \*\*\* |  |
| B13当他人遇到困难时，我愿意伸出援手 | <--- | Character\_strength | 1.011 | .064 | 15.887 | \*\*\* |  |
| Interpersonalpromotion | <--- | Job\_performance | .991 | .048 | 20.440 | \*\*\* |  |
| Workcontribution | <--- | Job\_performance | 1.305 | .089 | 14.667 | \*\*\* |  |
| Personalgrowth | <--- | Job\_performance | 1.257 | .081 | 15.468 | \*\*\* |  |
| A11如果放弃现在的工作，我会觉得失去了意义 | <--- | Career\_calling | 1.107 | .071 | 15.688 | \*\*\* |  |
| A3我对我目前的职业感到非常满足 | <--- | Career\_calling | 1.016 | .051 | 19.751 | \*\*\* |  |
| A4我愿意为现在的工作而作出一些牺牲 | <--- | Career\_calling | .959 | .053 | 17.943 | \*\*\* |  |
| A2与其它工作相比，我还是更喜欢现在的工作 | <--- | Career\_calling | .957 | .043 | 22.067 | \*\*\* |  |
| A1我热爱我现在的工作 | <--- | Career\_calling | 1.000 |  |
| A9我的职业一直存在于我的内心深处 | <--- | Career\_calling | 1.037 | .055 | 19.004 | \*\*\* |  |
| A5自我介绍时，我首先想起我的职业 | <--- | Career\_calling | .963 | .057 | 16.785 | \*\*\* |  |
| A12我目前的职业可以为我带来快乐 | <--- | Career\_calling | 1.163 | .067 | 17.317 | \*\*\* |  |
| A10我会经常思考工作相关的事 | <--- | Career\_calling | .915 | .055 | 16.668 | \*\*\* |  |
| B12同事和病人都认为我性格沉稳 | <--- | Character\_strength | .938 | .064 | 14.545 | \*\*\* |  |
| Professionalidentity | <--- | Job\_performance | 1.354 | .094 | 14.401 | \*\*\* |  |
| Medicalserviceperformance | <--- | Job\_performance | 1.000 |  |

## Standardized Regression Weights: (Group number 1 - Default model)

|  |  |  | Estimate |
| --- | --- | --- | --- |
| Career\_calling | <--- | Character\_strength | .656 |
| Job\_performance | <--- | Career\_calling | .422 |
| Job\_performance | <--- | Character\_strength | .499 |
| A6即便遇到阻碍，我仍然会选择继续现在的工作 | <--- | Career\_calling | .904 |
| A7我觉得现在的工作是我生命中的一部分 | <--- | Career\_calling | .855 |
| A8我的职业让我充满使命感 | <--- | Career\_calling | .869 |
| B15我总能乐观地发现它积极的一面 | <--- | Character\_strength | .691 |
| B14我公平地对待所有人，不管他们是什么身份 | <--- | Character\_strength | .870 |
| B13当他人遇到困难时，我愿意伸出援手 | <--- | Character\_strength | .884 |
| Interpersonalpromotion | <--- | Job\_performance | .761 |
| Workcontribution | <--- | Job\_performance | .760 |
| Personalgrowth | <--- | Job\_performance | .809 |
| A11如果放弃现在的工作，我会觉得失去了意义 | <--- | Career\_calling | .735 |
| A3我对我目前的职业感到非常满足 | <--- | Career\_calling | .745 |
| A4我愿意为现在的工作而作出一些牺牲 | <--- | Career\_calling | .756 |
| A2与其它工作相比，我还是更喜欢现在的工作 | <--- | Career\_calling | .730 |
| A1我热爱我现在的工作 | <--- | Career\_calling | .755 |
| A9我的职业一直存在于我的内心深处 | <--- | Career\_calling | .865 |
| A5自我介绍时，我首先想起我的职业 | <--- | Career\_calling | .779 |
| A12我目前的职业可以为我带来快乐 | <--- | Career\_calling | .800 |
| A10我会经常思考工作相关的事 | <--- | Career\_calling | .778 |
| B12同事和病人都认为我性格沉稳 | <--- | Character\_strength | .691 |
| Professionalidentity | <--- | Job\_performance | .756 |
| Medicalserviceperformance | <--- | Job\_performance | .739 |

## Covariances: (Group number 1 - Default model)

|  |  |  | Estimate | S.E. | C.R. | P | Label |
| --- | --- | --- | --- | --- | --- | --- | --- |
| e1 | <--> | e2 | .157 | .018 | 8.750 | \*\*\* |  |
| e17 | <--> | e18 | .056 | .009 | 6.582 | \*\*\* |  |
| e3 | <--> | e4 | .103 | .015 | 7.040 | \*\*\* |  |
| e11 | <--> | e12 | .123 | .020 | 6.288 | \*\*\* |  |
| e2 | <--> | e3 | .139 | .017 | 8.144 | \*\*\* |  |
| e1 | <--> | e3 | .111 | .017 | 6.703 | \*\*\* |  |
| e20 | <--> | e21 | .080 | .013 | 6.141 | \*\*\* |  |
| e6 | <--> | e10 | -.044 | .009 | -4.703 | \*\*\* |  |
| e2 | <--> | e8 | -.036 | .009 | -4.064 | \*\*\* |  |
| e3 | <--> | e12 | .051 | .013 | 3.976 | \*\*\* |  |
| e1 | <--> | e4 | .053 | .013 | 4.120 | \*\*\* |  |
| e13 | <--> | e16 | .058 | .016 | 3.641 | \*\*\* |  |

## Correlations: (Group number 1 - Default model)

|  |  |  | Estimate |
| --- | --- | --- | --- |
| e1 | <--> | e2 | .506 |
| e17 | <--> | e18 | .464 |
| e3 | <--> | e4 | .341 |
| e11 | <--> | e12 | .348 |
| e2 | <--> | e3 | .426 |
| e1 | <--> | e3 | .351 |
| e20 | <--> | e21 | .471 |
| e6 | <--> | e10 | -.274 |
| e2 | <--> | e8 | -.176 |
| e3 | <--> | e12 | .160 |
| e1 | <--> | e4 | .185 |
| e13 | <--> | e16 | .210 |

## Variances: (Group number 1 - Default model)

|  |  |  | Estimate | S.E. | C.R. | P | Label |
| --- | --- | --- | --- | --- | --- | --- | --- |
| Character\_strength |  |  | .268 | .035 | 7.687 | \*\*\* |  |
| e22 |  |  | .228 | .027 | 8.504 | \*\*\* |  |
| e23 |  |  | .047 | .007 | 6.509 | \*\*\* |  |
| e1 |  |  | .301 | .022 | 13.759 | \*\*\* |  |
| e2 |  |  | .320 | .024 | 13.610 | \*\*\* |  |
| e3 |  |  | .331 | .023 | 14.188 | \*\*\* |  |
| e4 |  |  | .276 | .020 | 13.599 | \*\*\* |  |
| e5 |  |  | .239 | .018 | 13.476 | \*\*\* |  |
| e6 |  |  | .118 | .010 | 11.312 | \*\*\* |  |
| e7 |  |  | .161 | .013 | 12.782 | \*\*\* |  |
| e8 |  |  | .134 | .011 | 12.483 | \*\*\* |  |
| e9 |  |  | .145 | .011 | 12.635 | \*\*\* |  |
| e10 |  |  | .218 | .017 | 13.173 | \*\*\* |  |
| e11 |  |  | .416 | .030 | 13.660 | \*\*\* |  |
| e12 |  |  | .303 | .023 | 13.391 | \*\*\* |  |
| e13 |  |  | .293 | .023 | 12.837 | \*\*\* |  |
| e14 |  |  | .094 | .010 | 9.167 | \*\*\* |  |
| e15 |  |  | .077 | .009 | 8.467 | \*\*\* |  |
| e16 |  |  | .258 | .020 | 12.839 | \*\*\* |  |
| e17 |  |  | .131 | .011 | 11.731 | \*\*\* |  |
| e18 |  |  | .113 | .010 | 11.416 | \*\*\* |  |
| e19 |  |  | .197 | .017 | 11.604 | \*\*\* |  |
| e20 |  |  | .132 | .013 | 10.313 | \*\*\* |  |
| e21 |  |  | .217 | .019 | 11.289 | \*\*\* |  |

## Matrices (Group number 1 - Default model)

## Total Effects (Group number 1 - Default model)

|  | Character\_strength | Career\_calling | Job\_performance |
| --- | --- | --- | --- |
| Career\_calling | .801 | .000 | .000 |
| Job\_performance | .595 | .265 | .000 |
| Professionalidentity | .805 | .358 | 1.354 |
| Personalgrowth | .747 | .333 | 1.257 |
| Workcontribution | .776 | .346 | 1.305 |
| Interpersonalpromotion | .589 | .262 | .991 |
| Medicalserviceperformance | .595 | .265 | 1.000 |
| B12同事和病人都认为我性格沉稳 | .938 | .000 | .000 |
| B13当他人遇到困难时，我愿意伸出援手 | 1.011 | .000 | .000 |
| B14我公平地对待所有人，不管他们是什么身份 | 1.044 | .000 | .000 |
| B15我总能乐观地发现它积极的一面 | 1.000 | .000 | .000 |
| A12我目前的职业可以为我带来快乐 | .931 | 1.163 | .000 |
| A11如果放弃现在的工作，我会觉得失去了意义 | .887 | 1.107 | .000 |
| A10我会经常思考工作相关的事 | .732 | .915 | .000 |
| A9我的职业一直存在于我的内心深处 | .831 | 1.037 | .000 |
| A8我的职业让我充满使命感 | .817 | 1.020 | .000 |
| A7我觉得现在的工作是我生命中的一部分 | .839 | 1.047 | .000 |
| A6即便遇到阻碍，我仍然会选择继续现在的工作 | .921 | 1.151 | .000 |
| A5自我介绍时，我首先想起我的职业 | .771 | .963 | .000 |
| A4我愿意为现在的工作而作出一些牺牲 | .768 | .959 | .000 |
| A3我对我目前的职业感到非常满足 | .814 | 1.016 | .000 |
| A2与其它工作相比，我还是更喜欢现在的工作 | .766 | .957 | .000 |
| A1我热爱我现在的工作 | .801 | 1.000 | .000 |

## Standardized Total Effects (Group number 1 - Default model)

|  | Character\_strength | Career\_calling | Job\_performance |
| --- | --- | --- | --- |
| Career\_calling | .656 | .000 | .000 |
| Job\_performance | .775 | .422 | .000 |
| Professionalidentity | .586 | .319 | .756 |
| Personalgrowth | .627 | .341 | .809 |
| Workcontribution | .589 | .320 | .760 |
| Interpersonalpromotion | .590 | .321 | .761 |
| Medicalserviceperformance | .572 | .311 | .739 |
| B12同事和病人都认为我性格沉稳 | .691 | .000 | .000 |
| B13当他人遇到困难时，我愿意伸出援手 | .884 | .000 | .000 |
| B14我公平地对待所有人，不管他们是什么身份 | .870 | .000 | .000 |
| B15我总能乐观地发现它积极的一面 | .691 | .000 | .000 |
| A12我目前的职业可以为我带来快乐 | .525 | .800 | .000 |
| A11如果放弃现在的工作，我会觉得失去了意义 | .482 | .735 | .000 |
| A10我会经常思考工作相关的事 | .510 | .778 | .000 |
| A9我的职业一直存在于我的内心深处 | .567 | .865 | .000 |
| A8我的职业让我充满使命感 | .570 | .869 | .000 |
| A7我觉得现在的工作是我生命中的一部分 | .561 | .855 | .000 |
| A6即便遇到阻碍，我仍然会选择继续现在的工作 | .593 | .904 | .000 |
| A5自我介绍时，我首先想起我的职业 | .511 | .779 | .000 |
| A4我愿意为现在的工作而作出一些牺牲 | .495 | .756 | .000 |
| A3我对我目前的职业感到非常满足 | .488 | .745 | .000 |
| A2与其它工作相比，我还是更喜欢现在的工作 | .479 | .730 | .000 |
| A1我热爱我现在的工作 | .495 | .755 | .000 |

## Direct Effects (Group number 1 - Default model)

|  | Character\_strength | Career\_calling | Job\_performance |
| --- | --- | --- | --- |
| Career\_calling | .801 | .000 | .000 |
| Job\_performance | .382 | .265 | .000 |
| Professionalidentity | .000 | .000 | 1.354 |
| Personalgrowth | .000 | .000 | 1.257 |
| Workcontribution | .000 | .000 | 1.305 |
| Interpersonalpromotion | .000 | .000 | .991 |
| Medicalserviceperformance | .000 | .000 | 1.000 |
| B12同事和病人都认为我性格沉稳 | .938 | .000 | .000 |
| B13当他人遇到困难时，我愿意伸出援手 | 1.011 | .000 | .000 |
| B14我公平地对待所有人，不管他们是什么身份 | 1.044 | .000 | .000 |
| B15我总能乐观地发现它积极的一面 | 1.000 | .000 | .000 |
| A12我目前的职业可以为我带来快乐 | .000 | 1.163 | .000 |
| A11如果放弃现在的工作，我会觉得失去了意义 | .000 | 1.107 | .000 |
| A10我会经常思考工作相关的事 | .000 | .915 | .000 |
| A9我的职业一直存在于我的内心深处 | .000 | 1.037 | .000 |
| A8我的职业让我充满使命感 | .000 | 1.020 | .000 |
| A7我觉得现在的工作是我生命中的一部分 | .000 | 1.047 | .000 |
| A6即便遇到阻碍，我仍然会选择继续现在的工作 | .000 | 1.151 | .000 |
| A5自我介绍时，我首先想起我的职业 | .000 | .963 | .000 |
| A4我愿意为现在的工作而作出一些牺牲 | .000 | .959 | .000 |
| A3我对我目前的职业感到非常满足 | .000 | 1.016 | .000 |
| A2与其它工作相比，我还是更喜欢现在的工作 | .000 | .957 | .000 |
| A1我热爱我现在的工作 | .000 | 1.000 | .000 |

## Standardized Direct Effects (Group number 1 - Default model)

|  | Character\_strength | Career\_calling | Job\_performance |
| --- | --- | --- | --- |
| Career\_calling | .656 | .000 | .000 |
| Job\_performance | .499 | .422 | .000 |
| Professionalidentity | .000 | .000 | .756 |
| Personalgrowth | .000 | .000 | .809 |
| Workcontribution | .000 | .000 | .760 |
| Interpersonalpromotion | .000 | .000 | .761 |
| Medicalserviceperformance | .000 | .000 | .739 |
| B12同事和病人都认为我性格沉稳 | .691 | .000 | .000 |
| B13当他人遇到困难时，我愿意伸出援手 | .884 | .000 | .000 |
| B14我公平地对待所有人，不管他们是什么身份 | .870 | .000 | .000 |
| B15我总能乐观地发现它积极的一面 | .691 | .000 | .000 |
| A12我目前的职业可以为我带来快乐 | .000 | .800 | .000 |
| A11如果放弃现在的工作，我会觉得失去了意义 | .000 | .735 | .000 |
| A10我会经常思考工作相关的事 | .000 | .778 | .000 |
| A9我的职业一直存在于我的内心深处 | .000 | .865 | .000 |
| A8我的职业让我充满使命感 | .000 | .869 | .000 |
| A7我觉得现在的工作是我生命中的一部分 | .000 | .855 | .000 |
| A6即便遇到阻碍，我仍然会选择继续现在的工作 | .000 | .904 | .000 |
| A5自我介绍时，我首先想起我的职业 | .000 | .779 | .000 |
| A4我愿意为现在的工作而作出一些牺牲 | .000 | .756 | .000 |
| A3我对我目前的职业感到非常满足 | .000 | .745 | .000 |
| A2与其它工作相比，我还是更喜欢现在的工作 | .000 | .730 | .000 |
| A1我热爱我现在的工作 | .000 | .755 | .000 |

## Indirect Effects (Group number 1 - Default model)

|  | Character\_strength | Career\_calling | Job\_performance |
| --- | --- | --- | --- |
| Career\_calling | .000 | .000 | .000 |
| Job\_performance | .212 | .000 | .000 |
| Professionalidentity | .805 | .358 | .000 |
| Personalgrowth | .747 | .333 | .000 |
| Workcontribution | .776 | .346 | .000 |
| Interpersonalpromotion | .589 | .262 | .000 |
| Medicalserviceperformance | .595 | .265 | .000 |
| B12同事和病人都认为我性格沉稳 | .000 | .000 | .000 |
| B13当他人遇到困难时，我愿意伸出援手 | .000 | .000 | .000 |
| B14我公平地对待所有人，不管他们是什么身份 | .000 | .000 | .000 |
| B15我总能乐观地发现它积极的一面 | .000 | .000 | .000 |
| A12我目前的职业可以为我带来快乐 | .931 | .000 | .000 |
| A11如果放弃现在的工作，我会觉得失去了意义 | .887 | .000 | .000 |
| A10我会经常思考工作相关的事 | .732 | .000 | .000 |
| A9我的职业一直存在于我的内心深处 | .831 | .000 | .000 |
| A8我的职业让我充满使命感 | .817 | .000 | .000 |
| A7我觉得现在的工作是我生命中的一部分 | .839 | .000 | .000 |
| A6即便遇到阻碍，我仍然会选择继续现在的工作 | .921 | .000 | .000 |
| A5自我介绍时，我首先想起我的职业 | .771 | .000 | .000 |
| A4我愿意为现在的工作而作出一些牺牲 | .768 | .000 | .000 |
| A3我对我目前的职业感到非常满足 | .814 | .000 | .000 |
| A2与其它工作相比，我还是更喜欢现在的工作 | .766 | .000 | .000 |
| A1我热爱我现在的工作 | .801 | .000 | .000 |

## Standardized Indirect Effects (Group number 1 - Default model)

|  | Character\_strength | Career\_calling | Job\_performance |
| --- | --- | --- | --- |
| Career\_calling | .000 | .000 | .000 |
| Job\_performance | .276 | .000 | .000 |
| Professionalidentity | .586 | .319 | .000 |
| Personalgrowth | .627 | .341 | .000 |
| Workcontribution | .589 | .320 | .000 |
| Interpersonalpromotion | .590 | .321 | .000 |
| Medicalserviceperformance | .572 | .311 | .000 |
| B12同事和病人都认为我性格沉稳 | .000 | .000 | .000 |
| B13当他人遇到困难时，我愿意伸出援手 | .000 | .000 | .000 |
| B14我公平地对待所有人，不管他们是什么身份 | .000 | .000 | .000 |
| B15我总能乐观地发现它积极的一面 | .000 | .000 | .000 |
| A12我目前的职业可以为我带来快乐 | .525 | .000 | .000 |
| A11如果放弃现在的工作，我会觉得失去了意义 | .482 | .000 | .000 |
| A10我会经常思考工作相关的事 | .510 | .000 | .000 |
| A9我的职业一直存在于我的内心深处 | .567 | .000 | .000 |
| A8我的职业让我充满使命感 | .570 | .000 | .000 |
| A7我觉得现在的工作是我生命中的一部分 | .561 | .000 | .000 |
| A6即便遇到阻碍，我仍然会选择继续现在的工作 | .593 | .000 | .000 |
| A5自我介绍时，我首先想起我的职业 | .511 | .000 | .000 |
| A4我愿意为现在的工作而作出一些牺牲 | .495 | .000 | .000 |
| A3我对我目前的职业感到非常满足 | .488 | .000 | .000 |
| A2与其它工作相比，我还是更喜欢现在的工作 | .479 | .000 | .000 |
| A1我热爱我现在的工作 | .495 | .000 | .000 |

## Modification Indices (Group number 1 - Default model)

## Covariances: (Group number 1 - Default model)

|  |  |  | M.I. | Par Change |
| --- | --- | --- | --- | --- |
| e21 | <--> | e22 | 40.426 | .067 |
| e21 | <--> | e23 | 5.297 | -.013 |
| e20 | <--> | e22 | 9.689 | -.027 |
| e19 | <--> | e20 | 6.885 | -.021 |
| e17 | <--> | e22 | 7.991 | -.023 |
| e17 | <--> | e23 | 4.301 | .009 |
| e17 | <--> | e19 | 5.482 | .018 |
| e16 | <--> | e17 | 7.420 | .023 |
| e15 | <--> | e21 | 17.789 | -.030 |
| e15 | <--> | e20 | 5.231 | .013 |
| e15 | <--> | e19 | 11.326 | -.027 |
| e15 | <--> | e18 | 12.773 | .018 |
| e14 | <--> | e20 | 5.842 | .015 |
| e13 | <--> | e22 | 10.613 | .045 |
| e13 | <--> | e21 | 11.954 | .039 |
| e13 | <--> | e20 | 18.101 | -.039 |
| e13 | <--> | e19 | 37.798 | .080 |
| e13 | <--> | e17 | 6.224 | -.022 |
| e12 | <--> | e18 | 6.935 | -.021 |
| e12 | <--> | e16 | 6.491 | .033 |
| e11 | <--> | e23 | 5.535 | .020 |
| e11 | <--> | e21 | 16.019 | .050 |
| e11 | <--> | e20 | 4.673 | -.022 |
| e11 | <--> | e19 | 8.064 | .041 |
| e11 | <--> | e15 | 10.050 | -.032 |
| e11 | <--> | e13 | 8.576 | .048 |
| e10 | <--> | Character\_strength | 4.253 | .027 |
| e10 | <--> | e19 | 5.592 | .027 |
| e10 | <--> | e13 | 12.030 | .045 |
| e10 | <--> | e11 | 7.176 | .039 |
| e8 | <--> | e23 | 5.912 | -.013 |
| e8 | <--> | e19 | 9.505 | -.028 |
| e8 | <--> | e16 | 9.963 | -.031 |
| e8 | <--> | e11 | 8.365 | -.033 |
| e8 | <--> | e9 | 6.070 | .019 |
| e7 | <--> | e20 | 4.388 | -.015 |
| e7 | <--> | e13 | 4.012 | .023 |
| e7 | <--> | e11 | 8.578 | .037 |
| e6 | <--> | e17 | 6.365 | -.016 |
| e6 | <--> | e13 | 7.906 | .029 |
| e5 | <--> | e16 | 13.878 | -.047 |
| e5 | <--> | e10 | 8.312 | -.034 |
| e4 | <--> | e13 | 8.001 | -.037 |
| e4 | <--> | e9 | 11.955 | -.033 |
| e3 | <--> | e23 | 8.991 | .020 |
| e3 | <--> | e21 | 8.077 | .027 |
| e3 | <--> | e14 | 5.905 | -.020 |
| e3 | <--> | e11 | 9.355 | .043 |
| e3 | <--> | e9 | 5.699 | -.022 |
| e2 | <--> | e23 | 5.092 | -.014 |
| e2 | <--> | e16 | 14.106 | .042 |
| e2 | <--> | e13 | 4.765 | -.026 |
| e2 | <--> | e12 | 7.660 | .032 |
| e2 | <--> | e11 | 9.534 | -.041 |
| e2 | <--> | e4 | 12.171 | .037 |
| e1 | <--> | e20 | 4.031 | .015 |
| e1 | <--> | e13 | 4.936 | -.027 |
| e1 | <--> | e11 | 7.347 | -.037 |

## Variances: (Group number 1 - Default model)

|  |  |  | M.I. | Par Change |
| --- | --- | --- | --- | --- |

## Regression Weights: (Group number 1 - Default model)

|  |  |  | M.I. | Par Change |
| --- | --- | --- | --- | --- |
| Professionalidentity | <--- | Career\_calling | 14.154 | .125 |
| Professionalidentity | <--- | B13当他人遇到困难时，我愿意伸出援手 | 7.042 | -.092 |
| Professionalidentity | <--- | A12我目前的职业可以为我带来快乐 | 29.624 | .122 |
| Professionalidentity | <--- | A11如果放弃现在的工作，我会觉得失去了意义 | 37.078 | .132 |
| Professionalidentity | <--- | A9我的职业一直存在于我的内心深处 | 9.737 | .085 |
| Professionalidentity | <--- | A8我的职业让我充满使命感 | 7.113 | .074 |
| Professionalidentity | <--- | A7我觉得现在的工作是我生命中的一部分 | 14.106 | .100 |
| Professionalidentity | <--- | A6即便遇到阻碍，我仍然会选择继续现在的工作 | 16.775 | .105 |
| Professionalidentity | <--- | A5自我介绍时，我首先想起我的职业 | 7.315 | .071 |
| Professionalidentity | <--- | A4我愿意为现在的工作而作出一些牺牲 | 4.999 | .058 |
| Professionalidentity | <--- | A3我对我目前的职业感到非常满足 | 22.849 | .114 |
| Professionalidentity | <--- | A2与其它工作相比，我还是更喜欢现在的工作 | 14.678 | .095 |
| Professionalidentity | <--- | A1我热爱我现在的工作 | 7.426 | .067 |
| Personalgrowth | <--- | B15我总能乐观地发现它积极的一面 | 6.423 | -.056 |
| Personalgrowth | <--- | A11如果放弃现在的工作，我会觉得失去了意义 | 7.424 | -.048 |
| Personalgrowth | <--- | A9我的职业一直存在于我的内心深处 | 5.386 | -.051 |
| Personalgrowth | <--- | A7我觉得现在的工作是我生命中的一部分 | 5.991 | -.053 |
| Workcontribution | <--- | B12同事和病人都认为我性格沉稳 | 5.639 | .081 |
| Workcontribution | <--- | B15我总能乐观地发现它积极的一面 | 22.489 | .151 |
| Workcontribution | <--- | A11如果放弃现在的工作，我会觉得失去了意义 | 4.433 | .053 |
| Medicalserviceperformance | <--- | Career\_calling | 4.215 | -.054 |
| Medicalserviceperformance | <--- | A11如果放弃现在的工作，我会觉得失去了意义 | 5.121 | -.038 |
| Medicalserviceperformance | <--- | A6即便遇到阻碍，我仍然会选择继续现在的工作 | 7.251 | -.054 |
| B12同事和病人都认为我性格沉稳 | <--- | Medicalserviceperformance | 4.494 | .099 |
| B12同事和病人都认为我性格沉稳 | <--- | A12我目前的职业可以为我带来快乐 | 4.286 | .057 |
| B12同事和病人都认为我性格沉稳 | <--- | A3我对我目前的职业感到非常满足 | 5.163 | .066 |
| B12同事和病人都认为我性格沉稳 | <--- | A2与其它工作相比，我还是更喜欢现在的工作 | 12.571 | .108 |
| B13当他人遇到困难时，我愿意伸出援手 | <--- | Professionalidentity | 8.408 | -.069 |
| B13当他人遇到困难时，我愿意伸出援手 | <--- | Workcontribution | 6.731 | -.064 |
| B13当他人遇到困难时，我愿意伸出援手 | <--- | A11如果放弃现在的工作，我会觉得失去了意义 | 8.979 | -.053 |
| B14我公平地对待所有人，不管他们是什么身份 | <--- | A3我对我目前的职业感到非常满足 | 8.302 | -.060 |
| B14我公平地对待所有人，不管他们是什么身份 | <--- | A2与其它工作相比，我还是更喜欢现在的工作 | 6.318 | -.055 |
| B15我总能乐观地发现它积极的一面 | <--- | Career\_calling | 5.479 | .101 |
| B15我总能乐观地发现它积极的一面 | <--- | Workcontribution | 19.423 | .173 |
| B15我总能乐观地发现它积极的一面 | <--- | A12我目前的职业可以为我带来快乐 | 4.896 | .065 |
| B15我总能乐观地发现它积极的一面 | <--- | A11如果放弃现在的工作，我会觉得失去了意义 | 13.277 | .103 |
| B15我总能乐观地发现它积极的一面 | <--- | A10我会经常思考工作相关的事 | 11.939 | .125 |
| B15我总能乐观地发现它积极的一面 | <--- | A7我觉得现在的工作是我生命中的一部分 | 8.676 | .102 |
| B15我总能乐观地发现它积极的一面 | <--- | A6即便遇到阻碍，我仍然会选择继续现在的工作 | 7.639 | .092 |
| A12我目前的职业可以为我带来快乐 | <--- | A2与其它工作相比，我还是更喜欢现在的工作 | 7.076 | .082 |
| A11如果放弃现在的工作，我会觉得失去了意义 | <--- | Professionalidentity | 11.166 | .141 |
| A11如果放弃现在的工作，我会觉得失去了意义 | <--- | Workcontribution | 8.060 | .125 |
| A11如果放弃现在的工作，我会觉得失去了意义 | <--- | B15我总能乐观地发现它积极的一面 | 8.547 | .117 |
| A11如果放弃现在的工作，我会觉得失去了意义 | <--- | A2与其它工作相比，我还是更喜欢现在的工作 | 5.550 | -.085 |
| A11如果放弃现在的工作，我会觉得失去了意义 | <--- | A1我热爱我现在的工作 | 6.978 | -.094 |
| A10我会经常思考工作相关的事 | <--- | Character\_strength | 4.253 | .100 |
| A10我会经常思考工作相关的事 | <--- | Workcontribution | 5.594 | .083 |
| A10我会经常思考工作相关的事 | <--- | B15我总能乐观地发现它积极的一面 | 14.328 | .120 |
| A10我会经常思考工作相关的事 | <--- | A11如果放弃现在的工作，我会觉得失去了意义 | 4.049 | .050 |
| A9我的职业一直存在于我的内心深处 | <--- | A4我愿意为现在的工作而作出一些牺牲 | 8.899 | -.074 |
| A9我的职业一直存在于我的内心深处 | <--- | A3我对我目前的职业感到非常满足 | 6.978 | -.061 |
| A8我的职业让我充满使命感 | <--- | Workcontribution | 8.799 | -.083 |
| A8我的职业让我充满使命感 | <--- | B12同事和病人都认为我性格沉稳 | 9.537 | -.084 |
| A7我觉得现在的工作是我生命中的一部分 | <--- | A11如果放弃现在的工作，我会觉得失去了意义 | 4.071 | .044 |
| A5自我介绍时，我首先想起我的职业 | <--- | B12同事和病人都认为我性格沉稳 | 8.161 | -.101 |
| A4我愿意为现在的工作而作出一些牺牲 | <--- | A2与其它工作相比，我还是更喜欢现在的工作 | 8.938 | .087 |
| A3我对我目前的职业感到非常满足 | <--- | Professionalidentity | 9.247 | .099 |
| A3我对我目前的职业感到非常满足 | <--- | Personalgrowth | 4.303 | .077 |
| A3我对我目前的职业感到非常满足 | <--- | A11如果放弃现在的工作，我会觉得失去了意义 | 4.861 | .053 |
| A2与其它工作相比，我还是更喜欢现在的工作 | <--- | Personalgrowth | 4.238 | -.074 |
| A2与其它工作相比，我还是更喜欢现在的工作 | <--- | A4我愿意为现在的工作而作出一些牺牲 | 6.292 | .069 |

## Bootstrap (Group number 1 - Default model)

## Bootstrap standard errors (Group number 1 - Default model)

## Scalar Estimates (Group number 1 - Default model)

## Regression Weights: (Group number 1 - Default model)

| Parameter | | | SE | SE-SE | Mean | Bias | SE-Bias |
| --- | --- | --- | --- | --- | --- | --- | --- |
| Career\_calling | <--- | Character\_strength | .075 | .001 | .799 | -.001 | .001 |
| Job\_performance | <--- | Career\_calling | .050 | .000 | .268 | .003 | .001 |
| Job\_performance | <--- | Character\_strength | .071 | .001 | .384 | .001 | .001 |
| A6即便遇到阻碍，我仍然会选择继续现在的工作 | <--- | Career\_calling | .067 | .001 | 1.155 | .004 | .001 |
| A7我觉得现在的工作是我生命中的一部分 | <--- | Career\_calling | .070 | .001 | 1.051 | .004 | .001 |
| A8我的职业让我充满使命感 | <--- | Career\_calling | .056 | .001 | 1.024 | .004 | .001 |
| B15我总能乐观地发现它积极的一面 | <--- | Character\_strength | .000 | .000 | 1.000 | .000 | .000 |
| B14我公平地对待所有人，不管他们是什么身份 | <--- | Character\_strength | .074 | .001 | 1.046 | .002 | .001 |
| B13当他人遇到困难时，我愿意伸出援手 | <--- | Character\_strength | .076 | .001 | 1.013 | .002 | .001 |
| Interpersonalpromotion | <--- | Job\_performance | .051 | .001 | .993 | .002 | .001 |
| Workcontribution | <--- | Job\_performance | .092 | .001 | 1.308 | .003 | .001 |
| Personalgrowth | <--- | Job\_performance | .076 | .001 | 1.261 | .004 | .001 |
| A11如果放弃现在的工作，我会觉得失去了意义 | <--- | Career\_calling | .087 | .001 | 1.112 | .005 | .001 |
| A3我对我目前的职业感到非常满足 | <--- | Career\_calling | .057 | .001 | 1.019 | .002 | .001 |
| A4我愿意为现在的工作而作出一些牺牲 | <--- | Career\_calling | .055 | .001 | .960 | .002 | .001 |
| A2与其它工作相比，我还是更喜欢现在的工作 | <--- | Career\_calling | .049 | .000 | .958 | .001 | .001 |
| A1我热爱我现在的工作 | <--- | Career\_calling | .000 | .000 | 1.000 | .000 | .000 |
| A9我的职业一直存在于我的内心深处 | <--- | Career\_calling | .064 | .001 | 1.040 | .003 | .001 |
| A5自我介绍时，我首先想起我的职业 | <--- | Career\_calling | .073 | .001 | .966 | .004 | .001 |
| A12我目前的职业可以为我带来快乐 | <--- | Career\_calling | .076 | .001 | 1.167 | .004 | .001 |
| A10我会经常思考工作相关的事 | <--- | Career\_calling | .065 | .001 | .916 | .002 | .001 |
| B12同事和病人都认为我性格沉稳 | <--- | Character\_strength | .070 | .001 | .939 | .001 | .001 |
| Professionalidentity | <--- | Job\_performance | .102 | .001 | 1.359 | .005 | .001 |
| Medicalserviceperformance | <--- | Job\_performance | .000 | .000 | 1.000 | .000 | .000 |

## Standardized Regression Weights: (Group number 1 - Default model)

| Parameter | | | SE | SE-SE | Mean | Bias | SE-Bias |
| --- | --- | --- | --- | --- | --- | --- | --- |
| Career\_calling | <--- | Character\_strength | .048 | .000 | .655 | .000 | .001 |
| Job\_performance | <--- | Career\_calling | .066 | .001 | .424 | .002 | .001 |
| Job\_performance | <--- | Character\_strength | .070 | .001 | .497 | -.001 | .001 |
| A6即便遇到阻碍，我仍然会选择继续现在的工作 | <--- | Career\_calling | .013 | .000 | .904 | .000 | .000 |
| A7我觉得现在的工作是我生命中的一部分 | <--- | Career\_calling | .020 | .000 | .855 | .000 | .000 |
| A8我的职业让我充满使命感 | <--- | Career\_calling | .017 | .000 | .870 | .001 | .000 |
| B15我总能乐观地发现它积极的一面 | <--- | Character\_strength | .038 | .000 | .692 | .001 | .001 |
| B14我公平地对待所有人，不管他们是什么身份 | <--- | Character\_strength | .018 | .000 | .870 | .000 | .000 |
| B13当他人遇到困难时，我愿意伸出援手 | <--- | Character\_strength | .020 | .000 | .884 | .000 | .000 |
| Interpersonalpromotion | <--- | Job\_performance | .033 | .000 | .760 | .000 | .000 |
| Workcontribution | <--- | Job\_performance | .029 | .000 | .759 | .000 | .000 |
| Personalgrowth | <--- | Job\_performance | .023 | .000 | .808 | .000 | .000 |
| A11如果放弃现在的工作，我会觉得失去了意义 | <--- | Career\_calling | .031 | .000 | .735 | .000 | .000 |
| A3我对我目前的职业感到非常满足 | <--- | Career\_calling | .034 | .000 | .744 | .000 | .000 |
| A4我愿意为现在的工作而作出一些牺牲 | <--- | Career\_calling | .029 | .000 | .756 | .000 | .000 |
| A2与其它工作相比，我还是更喜欢现在的工作 | <--- | Career\_calling | .044 | .000 | .731 | .001 | .001 |
| A1我热爱我现在的工作 | <--- | Career\_calling | .037 | .000 | .755 | .000 | .001 |
| A9我的职业一直存在于我的内心深处 | <--- | Career\_calling | .019 | .000 | .865 | .000 | .000 |
| A5自我介绍时，我首先想起我的职业 | <--- | Career\_calling | .029 | .000 | .780 | .000 | .000 |
| A12我目前的职业可以为我带来快乐 | <--- | Career\_calling | .029 | .000 | .800 | .000 | .000 |
| A10我会经常思考工作相关的事 | <--- | Career\_calling | .033 | .000 | .775 | -.002 | .000 |
| B12同事和病人都认为我性格沉稳 | <--- | Character\_strength | .037 | .000 | .690 | .000 | .001 |
| Professionalidentity | <--- | Job\_performance | .031 | .000 | .756 | .001 | .000 |
| Medicalserviceperformance | <--- | Job\_performance | .032 | .000 | .737 | -.001 | .000 |

## Covariances: (Group number 1 - Default model)

| Parameter | | | SE | SE-SE | Mean | Bias | SE-Bias |
| --- | --- | --- | --- | --- | --- | --- | --- |
| e1 | <--> | e2 | .048 | .000 | .155 | -.002 | .001 |
| e17 | <--> | e18 | .010 | .000 | .056 | .000 | .000 |
| e3 | <--> | e4 | .027 | .000 | .103 | .000 | .000 |
| e11 | <--> | e12 | .033 | .000 | .123 | .000 | .000 |
| e2 | <--> | e3 | .048 | .000 | .137 | -.002 | .001 |
| e1 | <--> | e3 | .042 | .000 | .110 | -.001 | .001 |
| e20 | <--> | e21 | .017 | .000 | .079 | -.001 | .000 |
| e6 | <--> | e10 | .013 | .000 | -.043 | .001 | .000 |
| e2 | <--> | e8 | .013 | .000 | -.036 | .001 | .000 |
| e3 | <--> | e12 | .017 | .000 | .051 | .000 | .000 |
| e1 | <--> | e4 | .018 | .000 | .054 | .000 | .000 |
| e13 | <--> | e16 | .021 | .000 | .057 | -.001 | .000 |

## Correlations: (Group number 1 - Default model)

| Parameter | | | SE | SE-SE | Mean | Bias | SE-Bias |
| --- | --- | --- | --- | --- | --- | --- | --- |
| e1 | <--> | e2 | .090 | .001 | .497 | -.009 | .001 |
| e17 | <--> | e18 | .061 | .001 | .463 | -.001 | .001 |
| e3 | <--> | e4 | .075 | .001 | .342 | .001 | .001 |
| e11 | <--> | e12 | .066 | .001 | .346 | -.002 | .001 |
| e2 | <--> | e3 | .094 | .001 | .414 | -.012 | .001 |
| e1 | <--> | e3 | .101 | .001 | .345 | -.006 | .001 |
| e20 | <--> | e21 | .067 | .001 | .467 | -.004 | .001 |
| e6 | <--> | e10 | .074 | .001 | -.269 | .005 | .001 |
| e2 | <--> | e8 | .062 | .001 | -.175 | .001 | .001 |
| e3 | <--> | e12 | .052 | .001 | .162 | .003 | .001 |
| e1 | <--> | e4 | .062 | .001 | .189 | .004 | .001 |
| e13 | <--> | e16 | .071 | .001 | .208 | -.002 | .001 |

## Variances: (Group number 1 - Default model)

| Parameter | | | SE | SE-SE | Mean | Bias | SE-Bias |
| --- | --- | --- | --- | --- | --- | --- | --- |
| Character\_strength |  |  | .039 | .000 | .270 | .002 | .001 |
| e22 |  |  | .042 | .000 | .227 | -.001 | .001 |
| e23 |  |  | .010 | .000 | .046 | -.001 | .000 |
| e1 |  |  | .052 | .001 | .299 | -.002 | .001 |
| e2 |  |  | .060 | .001 | .318 | -.002 | .001 |
| e3 |  |  | .048 | .000 | .330 | -.001 | .001 |
| e4 |  |  | .038 | .000 | .274 | -.001 | .001 |
| e5 |  |  | .028 | .000 | .237 | -.003 | .000 |
| e6 |  |  | .013 | .000 | .117 | -.001 | .000 |
| e7 |  |  | .024 | .000 | .160 | .000 | .000 |
| e8 |  |  | .017 | .000 | .133 | -.001 | .000 |
| e9 |  |  | .016 | .000 | .144 | -.001 | .000 |
| e10 |  |  | .029 | .000 | .219 | .001 | .000 |
| e11 |  |  | .047 | .000 | .414 | -.002 | .001 |
| e12 |  |  | .046 | .000 | .302 | -.001 | .001 |
| e13 |  |  | .034 | .000 | .291 | -.002 | .000 |
| e14 |  |  | .013 | .000 | .093 | .000 | .000 |
| e15 |  |  | .012 | .000 | .076 | .000 | .000 |
| e16 |  |  | .032 | .000 | .257 | -.001 | .000 |
| e17 |  |  | .012 | .000 | .131 | .000 | .000 |
| e18 |  |  | .012 | .000 | .112 | -.001 | .000 |
| e19 |  |  | .022 | .000 | .195 | -.001 | .000 |
| e20 |  |  | .014 | .000 | .131 | -.001 | .000 |
| e21 |  |  | .029 | .000 | .215 | -.001 | .000 |

## Matrices (Group number 1 - Default model)

## Total Effects - Standard Errors (Group number 1 - Default model)

|  | Character\_strength | Career\_calling | Job\_performance |
| --- | --- | --- | --- |
| Career\_calling | .075 | .000 | .000 |
| Job\_performance | .067 | .050 | .000 |
| Professionalidentity | .083 | .076 | .102 |
| Personalgrowth | .078 | .065 | .076 |
| Workcontribution | .075 | .068 | .092 |
| Interpersonalpromotion | .064 | .048 | .051 |
| Medicalserviceperformance | .067 | .050 | .000 |
| B12同事和病人都认为我性格沉稳 | .070 | .000 | .000 |
| B13当他人遇到困难时，我愿意伸出援手 | .076 | .000 | .000 |
| B14我公平地对待所有人，不管他们是什么身份 | .074 | .000 | .000 |
| B15我总能乐观地发现它积极的一面 | .000 | .000 | .000 |
| A12我目前的职业可以为我带来快乐 | .074 | .076 | .000 |
| A11如果放弃现在的工作，我会觉得失去了意义 | .077 | .087 | .000 |
| A10我会经常思考工作相关的事 | .071 | .065 | .000 |
| A9我的职业一直存在于我的内心深处 | .074 | .064 | .000 |
| A8我的职业让我充满使命感 | .070 | .056 | .000 |
| A7我觉得现在的工作是我生命中的一部分 | .070 | .070 | .000 |
| A6即便遇到阻碍，我仍然会选择继续现在的工作 | .075 | .067 | .000 |
| A5自我介绍时，我首先想起我的职业 | .075 | .073 | .000 |
| A4我愿意为现在的工作而作出一些牺牲 | .077 | .055 | .000 |
| A3我对我目前的职业感到非常满足 | .072 | .057 | .000 |
| A2与其它工作相比，我还是更喜欢现在的工作 | .070 | .049 | .000 |
| A1我热爱我现在的工作 | .075 | .000 | .000 |

## Standardized Total Effects - Standard Errors (Group number 1 - Default model)

|  | Character\_strength | Career\_calling | Job\_performance |
| --- | --- | --- | --- |
| Career\_calling | .048 | .000 | .000 |
| Job\_performance | .038 | .066 | .000 |
| Professionalidentity | .038 | .057 | .031 |
| Personalgrowth | .036 | .055 | .023 |
| Workcontribution | .039 | .051 | .029 |
| Interpersonalpromotion | .039 | .049 | .033 |
| Medicalserviceperformance | .038 | .048 | .032 |
| B12同事和病人都认为我性格沉稳 | .037 | .000 | .000 |
| B13当他人遇到困难时，我愿意伸出援手 | .020 | .000 | .000 |
| B14我公平地对待所有人，不管他们是什么身份 | .018 | .000 | .000 |
| B15我总能乐观地发现它积极的一面 | .038 | .000 | .000 |
| A12我目前的职业可以为我带来快乐 | .041 | .029 | .000 |
| A11如果放弃现在的工作，我会觉得失去了意义 | .040 | .031 | .000 |
| A10我会经常思考工作相关的事 | .047 | .033 | .000 |
| A9我的职业一直存在于我的内心深处 | .043 | .019 | .000 |
| A8我的职业让我充满使命感 | .043 | .017 | .000 |
| A7我觉得现在的工作是我生命中的一部分 | .043 | .020 | .000 |
| A6即便遇到阻碍，我仍然会选择继续现在的工作 | .045 | .013 | .000 |
| A5自我介绍时，我首先想起我的职业 | .042 | .029 | .000 |
| A4我愿意为现在的工作而作出一些牺牲 | .045 | .029 | .000 |
| A3我对我目前的职业感到非常满足 | .040 | .034 | .000 |
| A2与其它工作相比，我还是更喜欢现在的工作 | .043 | .044 | .000 |
| A1我热爱我现在的工作 | .043 | .037 | .000 |

## Direct Effects - Standard Errors (Group number 1 - Default model)

|  | Character\_strength | Career\_calling | Job\_performance |
| --- | --- | --- | --- |
| Career\_calling | .075 | .000 | .000 |
| Job\_performance | .071 | .050 | .000 |
| Professionalidentity | .000 | .000 | .102 |
| Personalgrowth | .000 | .000 | .076 |
| Workcontribution | .000 | .000 | .092 |
| Interpersonalpromotion | .000 | .000 | .051 |
| Medicalserviceperformance | .000 | .000 | .000 |
| B12同事和病人都认为我性格沉稳 | .070 | .000 | .000 |
| B13当他人遇到困难时，我愿意伸出援手 | .076 | .000 | .000 |
| B14我公平地对待所有人，不管他们是什么身份 | .074 | .000 | .000 |
| B15我总能乐观地发现它积极的一面 | .000 | .000 | .000 |
| A12我目前的职业可以为我带来快乐 | .000 | .076 | .000 |
| A11如果放弃现在的工作，我会觉得失去了意义 | .000 | .087 | .000 |
| A10我会经常思考工作相关的事 | .000 | .065 | .000 |
| A9我的职业一直存在于我的内心深处 | .000 | .064 | .000 |
| A8我的职业让我充满使命感 | .000 | .056 | .000 |
| A7我觉得现在的工作是我生命中的一部分 | .000 | .070 | .000 |
| A6即便遇到阻碍，我仍然会选择继续现在的工作 | .000 | .067 | .000 |
| A5自我介绍时，我首先想起我的职业 | .000 | .073 | .000 |
| A4我愿意为现在的工作而作出一些牺牲 | .000 | .055 | .000 |
| A3我对我目前的职业感到非常满足 | .000 | .057 | .000 |
| A2与其它工作相比，我还是更喜欢现在的工作 | .000 | .049 | .000 |
| A1我热爱我现在的工作 | .000 | .000 | .000 |

## Standardized Direct Effects - Standard Errors (Group number 1 - Default model)

|  | Character\_strength | Career\_calling | Job\_performance |
| --- | --- | --- | --- |
| Career\_calling | .048 | .000 | .000 |
| Job\_performance | .070 | .066 | .000 |
| Professionalidentity | .000 | .000 | .031 |
| Personalgrowth | .000 | .000 | .023 |
| Workcontribution | .000 | .000 | .029 |
| Interpersonalpromotion | .000 | .000 | .033 |
| Medicalserviceperformance | .000 | .000 | .032 |
| B12同事和病人都认为我性格沉稳 | .037 | .000 | .000 |
| B13当他人遇到困难时，我愿意伸出援手 | .020 | .000 | .000 |
| B14我公平地对待所有人，不管他们是什么身份 | .018 | .000 | .000 |
| B15我总能乐观地发现它积极的一面 | .038 | .000 | .000 |
| A12我目前的职业可以为我带来快乐 | .000 | .029 | .000 |
| A11如果放弃现在的工作，我会觉得失去了意义 | .000 | .031 | .000 |
| A10我会经常思考工作相关的事 | .000 | .033 | .000 |
| A9我的职业一直存在于我的内心深处 | .000 | .019 | .000 |
| A8我的职业让我充满使命感 | .000 | .017 | .000 |
| A7我觉得现在的工作是我生命中的一部分 | .000 | .020 | .000 |
| A6即便遇到阻碍，我仍然会选择继续现在的工作 | .000 | .013 | .000 |
| A5自我介绍时，我首先想起我的职业 | .000 | .029 | .000 |
| A4我愿意为现在的工作而作出一些牺牲 | .000 | .029 | .000 |
| A3我对我目前的职业感到非常满足 | .000 | .034 | .000 |
| A2与其它工作相比，我还是更喜欢现在的工作 | .000 | .044 | .000 |
| A1我热爱我现在的工作 | .000 | .037 | .000 |

## Indirect Effects - Standard Errors (Group number 1 - Default model)

|  | Character\_strength | Career\_calling | Job\_performance |
| --- | --- | --- | --- |
| Career\_calling | .000 | .000 | .000 |
| Job\_performance | .039 | .000 | .000 |
| Professionalidentity | .083 | .076 | .000 |
| Personalgrowth | .078 | .065 | .000 |
| Workcontribution | .075 | .068 | .000 |
| Interpersonalpromotion | .064 | .048 | .000 |
| Medicalserviceperformance | .067 | .050 | .000 |
| B12同事和病人都认为我性格沉稳 | .000 | .000 | .000 |
| B13当他人遇到困难时，我愿意伸出援手 | .000 | .000 | .000 |
| B14我公平地对待所有人，不管他们是什么身份 | .000 | .000 | .000 |
| B15我总能乐观地发现它积极的一面 | .000 | .000 | .000 |
| A12我目前的职业可以为我带来快乐 | .074 | .000 | .000 |
| A11如果放弃现在的工作，我会觉得失去了意义 | .077 | .000 | .000 |
| A10我会经常思考工作相关的事 | .071 | .000 | .000 |
| A9我的职业一直存在于我的内心深处 | .074 | .000 | .000 |
| A8我的职业让我充满使命感 | .070 | .000 | .000 |
| A7我觉得现在的工作是我生命中的一部分 | .070 | .000 | .000 |
| A6即便遇到阻碍，我仍然会选择继续现在的工作 | .075 | .000 | .000 |
| A5自我介绍时，我首先想起我的职业 | .075 | .000 | .000 |
| A4我愿意为现在的工作而作出一些牺牲 | .077 | .000 | .000 |
| A3我对我目前的职业感到非常满足 | .072 | .000 | .000 |
| A2与其它工作相比，我还是更喜欢现在的工作 | .070 | .000 | .000 |
| A1我热爱我现在的工作 | .075 | .000 | .000 |

## Standardized Indirect Effects - Standard Errors (Group number 1 - Default model)

|  | Character\_strength | Career\_calling | Job\_performance |
| --- | --- | --- | --- |
| Career\_calling | .000 | .000 | .000 |
| Job\_performance | .048 | .000 | .000 |
| Professionalidentity | .038 | .057 | .000 |
| Personalgrowth | .036 | .055 | .000 |
| Workcontribution | .039 | .051 | .000 |
| Interpersonalpromotion | .039 | .049 | .000 |
| Medicalserviceperformance | .038 | .048 | .000 |
| B12同事和病人都认为我性格沉稳 | .000 | .000 | .000 |
| B13当他人遇到困难时，我愿意伸出援手 | .000 | .000 | .000 |
| B14我公平地对待所有人，不管他们是什么身份 | .000 | .000 | .000 |
| B15我总能乐观地发现它积极的一面 | .000 | .000 | .000 |
| A12我目前的职业可以为我带来快乐 | .041 | .000 | .000 |
| A11如果放弃现在的工作，我会觉得失去了意义 | .040 | .000 | .000 |
| A10我会经常思考工作相关的事 | .047 | .000 | .000 |
| A9我的职业一直存在于我的内心深处 | .043 | .000 | .000 |
| A8我的职业让我充满使命感 | .043 | .000 | .000 |
| A7我觉得现在的工作是我生命中的一部分 | .043 | .000 | .000 |
| A6即便遇到阻碍，我仍然会选择继续现在的工作 | .045 | .000 | .000 |
| A5自我介绍时，我首先想起我的职业 | .042 | .000 | .000 |
| A4我愿意为现在的工作而作出一些牺牲 | .045 | .000 | .000 |
| A3我对我目前的职业感到非常满足 | .040 | .000 | .000 |
| A2与其它工作相比，我还是更喜欢现在的工作 | .043 | .000 | .000 |
| A1我热爱我现在的工作 | .043 | .000 | .000 |

## Bootstrap Confidence (Group number 1 - Default model)

## Percentile method (Group number 1 - Default model)

## 95% confidence intervals (percentile method)

## Scalar Estimates (Group number 1 - Default model)

## Regression Weights: (Group number 1 - Default model)

| Parameter | | | Estimate | Lower | Upper | P |
| --- | --- | --- | --- | --- | --- | --- |
| Career\_calling | <--- | Character\_strength | .801 | .660 | .953 | .000 |
| Job\_performance | <--- | Career\_calling | .265 | .179 | .372 | .000 |
| Job\_performance | <--- | Character\_strength | .382 | .252 | .528 | .000 |
| A6即便遇到阻碍，我仍然会选择继续现在的工作 | <--- | Career\_calling | 1.151 | 1.033 | 1.295 | .000 |
| A7我觉得现在的工作是我生命中的一部分 | <--- | Career\_calling | 1.047 | .925 | 1.196 | .000 |
| A8我的职业让我充满使命感 | <--- | Career\_calling | 1.020 | .923 | 1.142 | .000 |
| B15我总能乐观地发现它积极的一面 | <--- | Character\_strength | 1.000 | 1.000 | 1.000 | ... |
| B14我公平地对待所有人，不管他们是什么身份 | <--- | Character\_strength | 1.044 | .911 | 1.203 | .000 |
| B13当他人遇到困难时，我愿意伸出援手 | <--- | Character\_strength | 1.011 | .871 | 1.170 | .000 |
| Interpersonalpromotion | <--- | Job\_performance | .991 | .897 | 1.100 | .000 |
| Workcontribution | <--- | Job\_performance | 1.305 | 1.140 | 1.505 | .000 |
| Personalgrowth | <--- | Job\_performance | 1.257 | 1.124 | 1.423 | .000 |
| A11如果放弃现在的工作，我会觉得失去了意义 | <--- | Career\_calling | 1.107 | .953 | 1.289 | .000 |
| A3我对我目前的职业感到非常满足 | <--- | Career\_calling | 1.016 | .913 | 1.137 | .000 |
| A4我愿意为现在的工作而作出一些牺牲 | <--- | Career\_calling | .959 | .858 | 1.074 | .000 |
| A2与其它工作相比，我还是更喜欢现在的工作 | <--- | Career\_calling | .957 | .866 | 1.059 | .000 |
| A1我热爱我现在的工作 | <--- | Career\_calling | 1.000 | 1.000 | 1.000 | ... |
| A9我的职业一直存在于我的内心深处 | <--- | Career\_calling | 1.037 | .917 | 1.173 | .000 |
| A5自我介绍时，我首先想起我的职业 | <--- | Career\_calling | .963 | .825 | 1.116 | .000 |
| A12我目前的职业可以为我带来快乐 | <--- | Career\_calling | 1.163 | 1.026 | 1.324 | .000 |
| A10我会经常思考工作相关的事 | <--- | Career\_calling | .915 | .797 | 1.049 | .000 |
| B12同事和病人都认为我性格沉稳 | <--- | Character\_strength | .938 | .807 | 1.083 | .000 |
| Professionalidentity | <--- | Job\_performance | 1.354 | 1.178 | 1.578 | .000 |
| Medicalserviceperformance | <--- | Job\_performance | 1.000 | 1.000 | 1.000 | ... |

## Standardized Regression Weights: (Group number 1 - Default model)

| Parameter | | | Estimate | Lower | Upper | P |
| --- | --- | --- | --- | --- | --- | --- |
| Career\_calling | <--- | Character\_strength | .656 | .554 | .745 | .000 |
| Job\_performance | <--- | Career\_calling | .422 | .294 | .552 | .000 |
| Job\_performance | <--- | Character\_strength | .499 | .357 | .629 | .000 |
| A6即便遇到阻碍，我仍然会选择继续现在的工作 | <--- | Career\_calling | .904 | .877 | .928 | .000 |
| A7我觉得现在的工作是我生命中的一部分 | <--- | Career\_calling | .855 | .811 | .891 | .000 |
| A8我的职业让我充满使命感 | <--- | Career\_calling | .869 | .836 | .901 | .000 |
| B15我总能乐观地发现它积极的一面 | <--- | Character\_strength | .691 | .614 | .763 | .000 |
| B14我公平地对待所有人，不管他们是什么身份 | <--- | Character\_strength | .870 | .832 | .904 | .000 |
| B13当他人遇到困难时，我愿意伸出援手 | <--- | Character\_strength | .884 | .839 | .920 | .000 |
| Interpersonalpromotion | <--- | Job\_performance | .761 | .693 | .820 | .000 |
| Workcontribution | <--- | Job\_performance | .760 | .701 | .813 | .000 |
| Personalgrowth | <--- | Job\_performance | .809 | .761 | .852 | .000 |
| A11如果放弃现在的工作，我会觉得失去了意义 | <--- | Career\_calling | .735 | .671 | .793 | .000 |
| A3我对我目前的职业感到非常满足 | <--- | Career\_calling | .745 | .671 | .808 | .000 |
| A4我愿意为现在的工作而作出一些牺牲 | <--- | Career\_calling | .756 | .697 | .811 | .000 |
| A2与其它工作相比，我还是更喜欢现在的工作 | <--- | Career\_calling | .730 | .638 | .809 | .000 |
| A1我热爱我现在的工作 | <--- | Career\_calling | .755 | .673 | .820 | .000 |
| A9我的职业一直存在于我的内心深处 | <--- | Career\_calling | .865 | .823 | .899 | .000 |
| A5自我介绍时，我首先想起我的职业 | <--- | Career\_calling | .779 | .718 | .834 | .000 |
| A12我目前的职业可以为我带来快乐 | <--- | Career\_calling | .800 | .735 | .852 | .000 |
| A10我会经常思考工作相关的事 | <--- | Career\_calling | .778 | .707 | .836 | .000 |
| B12同事和病人都认为我性格沉稳 | <--- | Character\_strength | .691 | .614 | .759 | .000 |
| Professionalidentity | <--- | Job\_performance | .756 | .691 | .813 | .000 |
| Medicalserviceperformance | <--- | Job\_performance | .739 | .673 | .795 | .000 |

## Covariances: (Group number 1 - Default model)

| Parameter | | | Estimate | Lower | Upper | P |
| --- | --- | --- | --- | --- | --- | --- |
| e1 | <--> | e2 | .157 | .078 | .260 | .000 |
| e17 | <--> | e18 | .056 | .037 | .076 | .000 |
| e3 | <--> | e4 | .103 | .056 | .163 | .000 |
| e11 | <--> | e12 | .123 | .068 | .196 | .000 |
| e2 | <--> | e3 | .139 | .061 | .243 | .000 |
| e1 | <--> | e3 | .111 | .044 | .204 | .000 |
| e20 | <--> | e21 | .080 | .048 | .114 | .000 |
| e6 | <--> | e10 | -.044 | -.068 | -.018 | .000 |
| e2 | <--> | e8 | -.036 | -.062 | -.010 | .005 |
| e3 | <--> | e12 | .051 | .019 | .085 | .001 |
| e1 | <--> | e4 | .053 | .019 | .091 | .001 |
| e13 | <--> | e16 | .058 | .017 | .098 | .005 |

## Correlations: (Group number 1 - Default model)

| Parameter | | | Estimate | Lower | Upper | P |
| --- | --- | --- | --- | --- | --- | --- |
| e1 | <--> | e2 | .506 | .315 | .662 | .000 |
| e17 | <--> | e18 | .464 | .338 | .578 | .000 |
| e3 | <--> | e4 | .341 | .202 | .494 | .000 |
| e11 | <--> | e12 | .348 | .220 | .475 | .000 |
| e2 | <--> | e3 | .426 | .230 | .593 | .000 |
| e1 | <--> | e3 | .351 | .153 | .536 | .000 |
| e20 | <--> | e21 | .471 | .329 | .589 | .000 |
| e6 | <--> | e10 | -.274 | -.412 | -.119 | .000 |
| e2 | <--> | e8 | -.176 | -.296 | -.052 | .005 |
| e3 | <--> | e12 | .160 | .062 | .267 | .001 |
| e1 | <--> | e4 | .185 | .068 | .312 | .001 |
| e13 | <--> | e16 | .210 | .067 | .343 | .005 |

## Variances: (Group number 1 - Default model)

| Parameter | | | Estimate | Lower | Upper | P |
| --- | --- | --- | --- | --- | --- | --- |
| Character\_strength |  |  | .268 | .195 | .350 | .000 |
| e22 |  |  | .228 | .153 | .317 | .000 |
| e23 |  |  | .047 | .028 | .067 | .000 |
| e1 |  |  | .301 | .213 | .411 | .000 |
| e2 |  |  | .320 | .214 | .448 | .000 |
| e3 |  |  | .331 | .244 | .433 | .000 |
| e4 |  |  | .276 | .206 | .351 | .000 |
| e5 |  |  | .239 | .184 | .295 | .000 |
| e6 |  |  | .118 | .092 | .143 | .000 |
| e7 |  |  | .161 | .118 | .211 | .000 |
| e8 |  |  | .134 | .103 | .170 | .000 |
| e9 |  |  | .145 | .113 | .177 | .000 |
| e10 |  |  | .218 | .165 | .280 | .000 |
| e11 |  |  | .416 | .324 | .512 | .000 |
| e12 |  |  | .303 | .222 | .401 | .000 |
| e13 |  |  | .293 | .227 | .361 | .000 |
| e14 |  |  | .094 | .069 | .120 | .000 |
| e15 |  |  | .077 | .054 | .102 | .000 |
| e16 |  |  | .258 | .199 | .324 | .000 |
| e17 |  |  | .131 | .108 | .155 | .000 |
| e18 |  |  | .113 | .088 | .136 | .000 |
| e19 |  |  | .197 | .156 | .240 | .000 |
| e20 |  |  | .132 | .104 | .161 | .000 |
| e21 |  |  | .217 | .164 | .275 | .000 |

## Matrices (Group number 1 - Default model)

## Total Effects (Group number 1 - Default model)

## Total Effects - Lower Bounds (PC) (Group number 1 - Default model)

|  | Character\_strength | Career\_calling | Job\_performance |
| --- | --- | --- | --- |
| Career\_calling | .660 | .000 | .000 |
| Job\_performance | .474 | .179 | .000 |
| Professionalidentity | .651 | .226 | 1.178 |
| Personalgrowth | .608 | .219 | 1.124 |
| Workcontribution | .635 | .229 | 1.140 |
| Interpersonalpromotion | .475 | .177 | .897 |
| Medicalserviceperformance | .474 | .179 | 1.000 |
| B12同事和病人都认为我性格沉稳 | .807 | .000 | .000 |
| B13当他人遇到困难时，我愿意伸出援手 | .871 | .000 | .000 |
| B14我公平地对待所有人，不管他们是什么身份 | .911 | .000 | .000 |
| B15我总能乐观地发现它积极的一面 | 1.000 | .000 | .000 |
| A12我目前的职业可以为我带来快乐 | .789 | 1.026 | .000 |
| A11如果放弃现在的工作，我会觉得失去了意义 | .738 | .953 | .000 |
| A10我会经常思考工作相关的事 | .595 | .797 | .000 |
| A9我的职业一直存在于我的内心深处 | .692 | .917 | .000 |
| A8我的职业让我充满使命感 | .686 | .923 | .000 |
| A7我觉得现在的工作是我生命中的一部分 | .705 | .925 | .000 |
| A6即便遇到阻碍，我仍然会选择继续现在的工作 | .779 | 1.033 | .000 |
| A5自我介绍时，我首先想起我的职业 | .628 | .825 | .000 |
| A4我愿意为现在的工作而作出一些牺牲 | .622 | .858 | .000 |
| A3我对我目前的职业感到非常满足 | .677 | .913 | .000 |
| A2与其它工作相比，我还是更喜欢现在的工作 | .634 | .866 | .000 |
| A1我热爱我现在的工作 | .660 | 1.000 | .000 |

## Total Effects - Upper Bounds (PC) (Group number 1 - Default model)

|  | Character\_strength | Career\_calling | Job\_performance |
| --- | --- | --- | --- |
| Career\_calling | .953 | .000 | .000 |
| Job\_performance | .735 | .372 | .000 |
| Professionalidentity | .979 | .523 | 1.578 |
| Personalgrowth | .914 | .476 | 1.423 |
| Workcontribution | .927 | .496 | 1.505 |
| Interpersonalpromotion | .726 | .366 | 1.100 |
| Medicalserviceperformance | .735 | .372 | 1.000 |
| B12同事和病人都认为我性格沉稳 | 1.083 | .000 | .000 |
| B13当他人遇到困难时，我愿意伸出援手 | 1.170 | .000 | .000 |
| B14我公平地对待所有人，不管他们是什么身份 | 1.203 | .000 | .000 |
| B15我总能乐观地发现它积极的一面 | 1.000 | .000 | .000 |
| A12我目前的职业可以为我带来快乐 | 1.080 | 1.324 | .000 |
| A11如果放弃现在的工作，我会觉得失去了意义 | 1.043 | 1.289 | .000 |
| A10我会经常思考工作相关的事 | .874 | 1.049 | .000 |
| A9我的职业一直存在于我的内心深处 | .981 | 1.173 | .000 |
| A8我的职业让我充满使命感 | .963 | 1.142 | .000 |
| A7我觉得现在的工作是我生命中的一部分 | .984 | 1.196 | .000 |
| A6即便遇到阻碍，我仍然会选择继续现在的工作 | 1.073 | 1.295 | .000 |
| A5自我介绍时，我首先想起我的职业 | .923 | 1.116 | .000 |
| A4我愿意为现在的工作而作出一些牺牲 | .928 | 1.074 | .000 |
| A3我对我目前的职业感到非常满足 | .964 | 1.137 | .000 |
| A2与其它工作相比，我还是更喜欢现在的工作 | .908 | 1.059 | .000 |
| A1我热爱我现在的工作 | .953 | 1.000 | .000 |

## Total Effects - Two Tailed Significance (PC) (Group number 1 - Default model)

|  | Character\_strength | Career\_calling | Job\_performance |
| --- | --- | --- | --- |
| Career\_calling | .000 | ... | ... |
| Job\_performance | .000 | .000 | ... |
| Professionalidentity | .000 | .000 | .000 |
| Personalgrowth | .000 | .000 | .000 |
| Workcontribution | .000 | .000 | .000 |
| Interpersonalpromotion | .000 | .000 | .000 |
| Medicalserviceperformance | .000 | .000 | ... |
| B12同事和病人都认为我性格沉稳 | .000 | ... | ... |
| B13当他人遇到困难时，我愿意伸出援手 | .000 | ... | ... |
| B14我公平地对待所有人，不管他们是什么身份 | .000 | ... | ... |
| B15我总能乐观地发现它积极的一面 | ... | ... | ... |
| A12我目前的职业可以为我带来快乐 | .000 | .000 | ... |
| A11如果放弃现在的工作，我会觉得失去了意义 | .000 | .000 | ... |
| A10我会经常思考工作相关的事 | .000 | .000 | ... |
| A9我的职业一直存在于我的内心深处 | .000 | .000 | ... |
| A8我的职业让我充满使命感 | .000 | .000 | ... |
| A7我觉得现在的工作是我生命中的一部分 | .000 | .000 | ... |
| A6即便遇到阻碍，我仍然会选择继续现在的工作 | .000 | .000 | ... |
| A5自我介绍时，我首先想起我的职业 | .000 | .000 | ... |
| A4我愿意为现在的工作而作出一些牺牲 | .000 | .000 | ... |
| A3我对我目前的职业感到非常满足 | .000 | .000 | ... |
| A2与其它工作相比，我还是更喜欢现在的工作 | .000 | .000 | ... |
| A1我热爱我现在的工作 | .000 | ... | ... |

## Standardized Total Effects (Group number 1 - Default model)

## Standardized Total Effects - Lower Bounds (PC) (Group number 1 - Default model)

|  | Character\_strength | Career\_calling | Job\_performance |
| --- | --- | --- | --- |
| Career\_calling | .554 | .000 | .000 |
| Job\_performance | .698 | .294 | .000 |
| Professionalidentity | .510 | .212 | .691 |
| Personalgrowth | .554 | .236 | .761 |
| Workcontribution | .511 | .223 | .701 |
| Interpersonalpromotion | .513 | .226 | .693 |
| Medicalserviceperformance | .496 | .220 | .673 |
| B12同事和病人都认为我性格沉稳 | .614 | .000 | .000 |
| B13当他人遇到困难时，我愿意伸出援手 | .839 | .000 | .000 |
| B14我公平地对待所有人，不管他们是什么身份 | .832 | .000 | .000 |
| B15我总能乐观地发现它积极的一面 | .614 | .000 | .000 |
| A12我目前的职业可以为我带来快乐 | .442 | .735 | .000 |
| A11如果放弃现在的工作，我会觉得失去了意义 | .403 | .671 | .000 |
| A10我会经常思考工作相关的事 | .414 | .707 | .000 |
| A9我的职业一直存在于我的内心深处 | .478 | .823 | .000 |
| A8我的职业让我充满使命感 | .482 | .836 | .000 |
| A7我觉得现在的工作是我生命中的一部分 | .473 | .811 | .000 |
| A6即便遇到阻碍，我仍然会选择继续现在的工作 | .502 | .877 | .000 |
| A5自我介绍时，我首先想起我的职业 | .427 | .718 | .000 |
| A4我愿意为现在的工作而作出一些牺牲 | .405 | .697 | .000 |
| A3我对我目前的职业感到非常满足 | .408 | .671 | .000 |
| A2与其它工作相比，我还是更喜欢现在的工作 | .393 | .638 | .000 |
| A1我热爱我现在的工作 | .411 | .673 | .000 |

## Standardized Total Effects - Upper Bounds (PC) (Group number 1 - Default model)

|  | Character\_strength | Career\_calling | Job\_performance |
| --- | --- | --- | --- |
| Career\_calling | .745 | .000 | .000 |
| Job\_performance | .848 | .552 | .000 |
| Professionalidentity | .659 | .434 | .813 |
| Personalgrowth | .696 | .450 | .852 |
| Workcontribution | .665 | .423 | .813 |
| Interpersonalpromotion | .665 | .418 | .820 |
| Medicalserviceperformance | .646 | .408 | .795 |
| B12同事和病人都认为我性格沉稳 | .759 | .000 | .000 |
| B13当他人遇到困难时，我愿意伸出援手 | .920 | .000 | .000 |
| B14我公平地对待所有人，不管他们是什么身份 | .904 | .000 | .000 |
| B15我总能乐观地发现它积极的一面 | .763 | .000 | .000 |
| A12我目前的职业可以为我带来快乐 | .603 | .852 | .000 |
| A11如果放弃现在的工作，我会觉得失去了意义 | .559 | .793 | .000 |
| A10我会经常思考工作相关的事 | .599 | .836 | .000 |
| A9我的职业一直存在于我的内心深处 | .650 | .899 | .000 |
| A8我的职业让我充满使命感 | .653 | .901 | .000 |
| A7我觉得现在的工作是我生命中的一部分 | .640 | .891 | .000 |
| A6即便遇到阻碍，我仍然会选择继续现在的工作 | .674 | .928 | .000 |
| A5自我介绍时，我首先想起我的职业 | .592 | .834 | .000 |
| A4我愿意为现在的工作而作出一些牺牲 | .582 | .811 | .000 |
| A3我对我目前的职业感到非常满足 | .566 | .808 | .000 |
| A2与其它工作相比，我还是更喜欢现在的工作 | .562 | .809 | .000 |
| A1我热爱我现在的工作 | .580 | .820 | .000 |

## Standardized Total Effects - Two Tailed Significance (PC) (Group number 1 - Default model)

|  | Character\_strength | Career\_calling | Job\_performance |
| --- | --- | --- | --- |
| Career\_calling | .000 | ... | ... |
| Job\_performance | .000 | .000 | ... |
| Professionalidentity | .000 | .000 | .000 |
| Personalgrowth | .000 | .000 | .000 |
| Workcontribution | .000 | .000 | .000 |
| Interpersonalpromotion | .000 | .000 | .000 |
| Medicalserviceperformance | .000 | .000 | .000 |
| B12同事和病人都认为我性格沉稳 | .000 | ... | ... |
| B13当他人遇到困难时，我愿意伸出援手 | .000 | ... | ... |
| B14我公平地对待所有人，不管他们是什么身份 | .000 | ... | ... |
| B15我总能乐观地发现它积极的一面 | .000 | ... | ... |
| A12我目前的职业可以为我带来快乐 | .000 | .000 | ... |
| A11如果放弃现在的工作，我会觉得失去了意义 | .000 | .000 | ... |
| A10我会经常思考工作相关的事 | .000 | .000 | ... |
| A9我的职业一直存在于我的内心深处 | .000 | .000 | ... |
| A8我的职业让我充满使命感 | .000 | .000 | ... |
| A7我觉得现在的工作是我生命中的一部分 | .000 | .000 | ... |
| A6即便遇到阻碍，我仍然会选择继续现在的工作 | .000 | .000 | ... |
| A5自我介绍时，我首先想起我的职业 | .000 | .000 | ... |
| A4我愿意为现在的工作而作出一些牺牲 | .000 | .000 | ... |
| A3我对我目前的职业感到非常满足 | .000 | .000 | ... |
| A2与其它工作相比，我还是更喜欢现在的工作 | .000 | .000 | ... |
| A1我热爱我现在的工作 | .000 | .000 | ... |

## Direct Effects (Group number 1 - Default model)

## Direct Effects - Lower Bounds (PC) (Group number 1 - Default model)

|  | Character\_strength | Career\_calling | Job\_performance |
| --- | --- | --- | --- |
| Career\_calling | .660 | .000 | .000 |
| Job\_performance | .252 | .179 | .000 |
| Professionalidentity | .000 | .000 | 1.178 |
| Personalgrowth | .000 | .000 | 1.124 |
| Workcontribution | .000 | .000 | 1.140 |
| Interpersonalpromotion | .000 | .000 | .897 |
| Medicalserviceperformance | .000 | .000 | 1.000 |
| B12同事和病人都认为我性格沉稳 | .807 | .000 | .000 |
| B13当他人遇到困难时，我愿意伸出援手 | .871 | .000 | .000 |
| B14我公平地对待所有人，不管他们是什么身份 | .911 | .000 | .000 |
| B15我总能乐观地发现它积极的一面 | 1.000 | .000 | .000 |
| A12我目前的职业可以为我带来快乐 | .000 | 1.026 | .000 |
| A11如果放弃现在的工作，我会觉得失去了意义 | .000 | .953 | .000 |
| A10我会经常思考工作相关的事 | .000 | .797 | .000 |
| A9我的职业一直存在于我的内心深处 | .000 | .917 | .000 |
| A8我的职业让我充满使命感 | .000 | .923 | .000 |
| A7我觉得现在的工作是我生命中的一部分 | .000 | .925 | .000 |
| A6即便遇到阻碍，我仍然会选择继续现在的工作 | .000 | 1.033 | .000 |
| A5自我介绍时，我首先想起我的职业 | .000 | .825 | .000 |
| A4我愿意为现在的工作而作出一些牺牲 | .000 | .858 | .000 |
| A3我对我目前的职业感到非常满足 | .000 | .913 | .000 |
| A2与其它工作相比，我还是更喜欢现在的工作 | .000 | .866 | .000 |
| A1我热爱我现在的工作 | .000 | 1.000 | .000 |

## Direct Effects - Upper Bounds (PC) (Group number 1 - Default model)

|  | Character\_strength | Career\_calling | Job\_performance |
| --- | --- | --- | --- |
| Career\_calling | .953 | .000 | .000 |
| Job\_performance | .528 | .372 | .000 |
| Professionalidentity | .000 | .000 | 1.578 |
| Personalgrowth | .000 | .000 | 1.423 |
| Workcontribution | .000 | .000 | 1.505 |
| Interpersonalpromotion | .000 | .000 | 1.100 |
| Medicalserviceperformance | .000 | .000 | 1.000 |
| B12同事和病人都认为我性格沉稳 | 1.083 | .000 | .000 |
| B13当他人遇到困难时，我愿意伸出援手 | 1.170 | .000 | .000 |
| B14我公平地对待所有人，不管他们是什么身份 | 1.203 | .000 | .000 |
| B15我总能乐观地发现它积极的一面 | 1.000 | .000 | .000 |
| A12我目前的职业可以为我带来快乐 | .000 | 1.324 | .000 |
| A11如果放弃现在的工作，我会觉得失去了意义 | .000 | 1.289 | .000 |
| A10我会经常思考工作相关的事 | .000 | 1.049 | .000 |
| A9我的职业一直存在于我的内心深处 | .000 | 1.173 | .000 |
| A8我的职业让我充满使命感 | .000 | 1.142 | .000 |
| A7我觉得现在的工作是我生命中的一部分 | .000 | 1.196 | .000 |
| A6即便遇到阻碍，我仍然会选择继续现在的工作 | .000 | 1.295 | .000 |
| A5自我介绍时，我首先想起我的职业 | .000 | 1.116 | .000 |
| A4我愿意为现在的工作而作出一些牺牲 | .000 | 1.074 | .000 |
| A3我对我目前的职业感到非常满足 | .000 | 1.137 | .000 |
| A2与其它工作相比，我还是更喜欢现在的工作 | .000 | 1.059 | .000 |
| A1我热爱我现在的工作 | .000 | 1.000 | .000 |

## Direct Effects - Two Tailed Significance (PC) (Group number 1 - Default model)

|  | Character\_strength | Career\_calling | Job\_performance |
| --- | --- | --- | --- |
| Career\_calling | .000 | ... | ... |
| Job\_performance | .000 | .000 | ... |
| Professionalidentity | ... | ... | .000 |
| Personalgrowth | ... | ... | .000 |
| Workcontribution | ... | ... | .000 |
| Interpersonalpromotion | ... | ... | .000 |
| Medicalserviceperformance | ... | ... | ... |
| B12同事和病人都认为我性格沉稳 | .000 | ... | ... |
| B13当他人遇到困难时，我愿意伸出援手 | .000 | ... | ... |
| B14我公平地对待所有人，不管他们是什么身份 | .000 | ... | ... |
| B15我总能乐观地发现它积极的一面 | ... | ... | ... |
| A12我目前的职业可以为我带来快乐 | ... | .000 | ... |
| A11如果放弃现在的工作，我会觉得失去了意义 | ... | .000 | ... |
| A10我会经常思考工作相关的事 | ... | .000 | ... |
| A9我的职业一直存在于我的内心深处 | ... | .000 | ... |
| A8我的职业让我充满使命感 | ... | .000 | ... |
| A7我觉得现在的工作是我生命中的一部分 | ... | .000 | ... |
| A6即便遇到阻碍，我仍然会选择继续现在的工作 | ... | .000 | ... |
| A5自我介绍时，我首先想起我的职业 | ... | .000 | ... |
| A4我愿意为现在的工作而作出一些牺牲 | ... | .000 | ... |
| A3我对我目前的职业感到非常满足 | ... | .000 | ... |
| A2与其它工作相比，我还是更喜欢现在的工作 | ... | .000 | ... |
| A1我热爱我现在的工作 | ... | ... | ... |

## Standardized Direct Effects (Group number 1 - Default model)

## Standardized Direct Effects - Lower Bounds (PC) (Group number 1 - Default model)

|  | Character\_strength | Career\_calling | Job\_performance |
| --- | --- | --- | --- |
| Career\_calling | .554 | .000 | .000 |
| Job\_performance | .357 | .294 | .000 |
| Professionalidentity | .000 | .000 | .691 |
| Personalgrowth | .000 | .000 | .761 |
| Workcontribution | .000 | .000 | .701 |
| Interpersonalpromotion | .000 | .000 | .693 |
| Medicalserviceperformance | .000 | .000 | .673 |
| B12同事和病人都认为我性格沉稳 | .614 | .000 | .000 |
| B13当他人遇到困难时，我愿意伸出援手 | .839 | .000 | .000 |
| B14我公平地对待所有人，不管他们是什么身份 | .832 | .000 | .000 |
| B15我总能乐观地发现它积极的一面 | .614 | .000 | .000 |
| A12我目前的职业可以为我带来快乐 | .000 | .735 | .000 |
| A11如果放弃现在的工作，我会觉得失去了意义 | .000 | .671 | .000 |
| A10我会经常思考工作相关的事 | .000 | .707 | .000 |
| A9我的职业一直存在于我的内心深处 | .000 | .823 | .000 |
| A8我的职业让我充满使命感 | .000 | .836 | .000 |
| A7我觉得现在的工作是我生命中的一部分 | .000 | .811 | .000 |
| A6即便遇到阻碍，我仍然会选择继续现在的工作 | .000 | .877 | .000 |
| A5自我介绍时，我首先想起我的职业 | .000 | .718 | .000 |
| A4我愿意为现在的工作而作出一些牺牲 | .000 | .697 | .000 |
| A3我对我目前的职业感到非常满足 | .000 | .671 | .000 |
| A2与其它工作相比，我还是更喜欢现在的工作 | .000 | .638 | .000 |
| A1我热爱我现在的工作 | .000 | .673 | .000 |

## Standardized Direct Effects - Upper Bounds (PC) (Group number 1 - Default model)

|  | Character\_strength | Career\_calling | Job\_performance |
| --- | --- | --- | --- |
| Career\_calling | .745 | .000 | .000 |
| Job\_performance | .629 | .552 | .000 |
| Professionalidentity | .000 | .000 | .813 |
| Personalgrowth | .000 | .000 | .852 |
| Workcontribution | .000 | .000 | .813 |
| Interpersonalpromotion | .000 | .000 | .820 |
| Medicalserviceperformance | .000 | .000 | .795 |
| B12同事和病人都认为我性格沉稳 | .759 | .000 | .000 |
| B13当他人遇到困难时，我愿意伸出援手 | .920 | .000 | .000 |
| B14我公平地对待所有人，不管他们是什么身份 | .904 | .000 | .000 |
| B15我总能乐观地发现它积极的一面 | .763 | .000 | .000 |
| A12我目前的职业可以为我带来快乐 | .000 | .852 | .000 |
| A11如果放弃现在的工作，我会觉得失去了意义 | .000 | .793 | .000 |
| A10我会经常思考工作相关的事 | .000 | .836 | .000 |
| A9我的职业一直存在于我的内心深处 | .000 | .899 | .000 |
| A8我的职业让我充满使命感 | .000 | .901 | .000 |
| A7我觉得现在的工作是我生命中的一部分 | .000 | .891 | .000 |
| A6即便遇到阻碍，我仍然会选择继续现在的工作 | .000 | .928 | .000 |
| A5自我介绍时，我首先想起我的职业 | .000 | .834 | .000 |
| A4我愿意为现在的工作而作出一些牺牲 | .000 | .811 | .000 |
| A3我对我目前的职业感到非常满足 | .000 | .808 | .000 |
| A2与其它工作相比，我还是更喜欢现在的工作 | .000 | .809 | .000 |
| A1我热爱我现在的工作 | .000 | .820 | .000 |

## Standardized Direct Effects - Two Tailed Significance (PC) (Group number 1 - Default model)

|  | Character\_strength | Career\_calling | Job\_performance |
| --- | --- | --- | --- |
| Career\_calling | .000 | ... | ... |
| Job\_performance | .000 | .000 | ... |
| Professionalidentity | ... | ... | .000 |
| Personalgrowth | ... | ... | .000 |
| Workcontribution | ... | ... | .000 |
| Interpersonalpromotion | ... | ... | .000 |
| Medicalserviceperformance | ... | ... | .000 |
| B12同事和病人都认为我性格沉稳 | .000 | ... | ... |
| B13当他人遇到困难时，我愿意伸出援手 | .000 | ... | ... |
| B14我公平地对待所有人，不管他们是什么身份 | .000 | ... | ... |
| B15我总能乐观地发现它积极的一面 | .000 | ... | ... |
| A12我目前的职业可以为我带来快乐 | ... | .000 | ... |
| A11如果放弃现在的工作，我会觉得失去了意义 | ... | .000 | ... |
| A10我会经常思考工作相关的事 | ... | .000 | ... |
| A9我的职业一直存在于我的内心深处 | ... | .000 | ... |
| A8我的职业让我充满使命感 | ... | .000 | ... |
| A7我觉得现在的工作是我生命中的一部分 | ... | .000 | ... |
| A6即便遇到阻碍，我仍然会选择继续现在的工作 | ... | .000 | ... |
| A5自我介绍时，我首先想起我的职业 | ... | .000 | ... |
| A4我愿意为现在的工作而作出一些牺牲 | ... | .000 | ... |
| A3我对我目前的职业感到非常满足 | ... | .000 | ... |
| A2与其它工作相比，我还是更喜欢现在的工作 | ... | .000 | ... |
| A1我热爱我现在的工作 | ... | .000 | ... |

## Indirect Effects (Group number 1 - Default model)

## Indirect Effects - Lower Bounds (PC) (Group number 1 - Default model)

|  | Character\_strength | Career\_calling | Job\_performance |
| --- | --- | --- | --- |
| Career\_calling | .000 | .000 | .000 |
| Job\_performance | .143 | .000 | .000 |
| Professionalidentity | .651 | .226 | .000 |
| Personalgrowth | .608 | .219 | .000 |
| Workcontribution | .635 | .229 | .000 |
| Interpersonalpromotion | .475 | .177 | .000 |
| Medicalserviceperformance | .474 | .179 | .000 |
| B12同事和病人都认为我性格沉稳 | .000 | .000 | .000 |
| B13当他人遇到困难时，我愿意伸出援手 | .000 | .000 | .000 |
| B14我公平地对待所有人，不管他们是什么身份 | .000 | .000 | .000 |
| B15我总能乐观地发现它积极的一面 | .000 | .000 | .000 |
| A12我目前的职业可以为我带来快乐 | .789 | .000 | .000 |
| A11如果放弃现在的工作，我会觉得失去了意义 | .738 | .000 | .000 |
| A10我会经常思考工作相关的事 | .595 | .000 | .000 |
| A9我的职业一直存在于我的内心深处 | .692 | .000 | .000 |
| A8我的职业让我充满使命感 | .686 | .000 | .000 |
| A7我觉得现在的工作是我生命中的一部分 | .705 | .000 | .000 |
| A6即便遇到阻碍，我仍然会选择继续现在的工作 | .779 | .000 | .000 |
| A5自我介绍时，我首先想起我的职业 | .628 | .000 | .000 |
| A4我愿意为现在的工作而作出一些牺牲 | .622 | .000 | .000 |
| A3我对我目前的职业感到非常满足 | .677 | .000 | .000 |
| A2与其它工作相比，我还是更喜欢现在的工作 | .634 | .000 | .000 |
| A1我热爱我现在的工作 | .660 | .000 | .000 |

## Indirect Effects - Upper Bounds (PC) (Group number 1 - Default model)

|  | Character\_strength | Career\_calling | Job\_performance |
| --- | --- | --- | --- |
| Career\_calling | .000 | .000 | .000 |
| Job\_performance | .295 | .000 | .000 |
| Professionalidentity | .979 | .523 | .000 |
| Personalgrowth | .914 | .476 | .000 |
| Workcontribution | .927 | .496 | .000 |
| Interpersonalpromotion | .726 | .366 | .000 |
| Medicalserviceperformance | .735 | .372 | .000 |
| B12同事和病人都认为我性格沉稳 | .000 | .000 | .000 |
| B13当他人遇到困难时，我愿意伸出援手 | .000 | .000 | .000 |
| B14我公平地对待所有人，不管他们是什么身份 | .000 | .000 | .000 |
| B15我总能乐观地发现它积极的一面 | .000 | .000 | .000 |
| A12我目前的职业可以为我带来快乐 | 1.080 | .000 | .000 |
| A11如果放弃现在的工作，我会觉得失去了意义 | 1.043 | .000 | .000 |
| A10我会经常思考工作相关的事 | .874 | .000 | .000 |
| A9我的职业一直存在于我的内心深处 | .981 | .000 | .000 |
| A8我的职业让我充满使命感 | .963 | .000 | .000 |
| A7我觉得现在的工作是我生命中的一部分 | .984 | .000 | .000 |
| A6即便遇到阻碍，我仍然会选择继续现在的工作 | 1.073 | .000 | .000 |
| A5自我介绍时，我首先想起我的职业 | .923 | .000 | .000 |
| A4我愿意为现在的工作而作出一些牺牲 | .928 | .000 | .000 |
| A3我对我目前的职业感到非常满足 | .964 | .000 | .000 |
| A2与其它工作相比，我还是更喜欢现在的工作 | .908 | .000 | .000 |
| A1我热爱我现在的工作 | .953 | .000 | .000 |

## Indirect Effects - Two Tailed Significance (PC) (Group number 1 - Default model)

|  | Character\_strength | Career\_calling | Job\_performance |
| --- | --- | --- | --- |
| Career\_calling | ... | ... | ... |
| Job\_performance | .000 | ... | ... |
| Professionalidentity | .000 | .000 | ... |
| Personalgrowth | .000 | .000 | ... |
| Workcontribution | .000 | .000 | ... |
| Interpersonalpromotion | .000 | .000 | ... |
| Medicalserviceperformance | .000 | .000 | ... |
| B12同事和病人都认为我性格沉稳 | ... | ... | ... |
| B13当他人遇到困难时，我愿意伸出援手 | ... | ... | ... |
| B14我公平地对待所有人，不管他们是什么身份 | ... | ... | ... |
| B15我总能乐观地发现它积极的一面 | ... | ... | ... |
| A12我目前的职业可以为我带来快乐 | .000 | ... | ... |
| A11如果放弃现在的工作，我会觉得失去了意义 | .000 | ... | ... |
| A10我会经常思考工作相关的事 | .000 | ... | ... |
| A9我的职业一直存在于我的内心深处 | .000 | ... | ... |
| A8我的职业让我充满使命感 | .000 | ... | ... |
| A7我觉得现在的工作是我生命中的一部分 | .000 | ... | ... |
| A6即便遇到阻碍，我仍然会选择继续现在的工作 | .000 | ... | ... |
| A5自我介绍时，我首先想起我的职业 | .000 | ... | ... |
| A4我愿意为现在的工作而作出一些牺牲 | .000 | ... | ... |
| A3我对我目前的职业感到非常满足 | .000 | ... | ... |
| A2与其它工作相比，我还是更喜欢现在的工作 | .000 | ... | ... |
| A1我热爱我现在的工作 | .000 | ... | ... |

## Standardized Indirect Effects (Group number 1 - Default model)

## Standardized Indirect Effects - Lower Bounds (PC) (Group number 1 - Default model)

|  | Character\_strength | Career\_calling | Job\_performance |
| --- | --- | --- | --- |
| Career\_calling | .000 | .000 | .000 |
| Job\_performance | .190 | .000 | .000 |
| Professionalidentity | .510 | .212 | .000 |
| Personalgrowth | .554 | .236 | .000 |
| Workcontribution | .511 | .223 | .000 |
| Interpersonalpromotion | .513 | .226 | .000 |
| Medicalserviceperformance | .496 | .220 | .000 |
| B12同事和病人都认为我性格沉稳 | .000 | .000 | .000 |
| B13当他人遇到困难时，我愿意伸出援手 | .000 | .000 | .000 |
| B14我公平地对待所有人，不管他们是什么身份 | .000 | .000 | .000 |
| B15我总能乐观地发现它积极的一面 | .000 | .000 | .000 |
| A12我目前的职业可以为我带来快乐 | .442 | .000 | .000 |
| A11如果放弃现在的工作，我会觉得失去了意义 | .403 | .000 | .000 |
| A10我会经常思考工作相关的事 | .414 | .000 | .000 |
| A9我的职业一直存在于我的内心深处 | .478 | .000 | .000 |
| A8我的职业让我充满使命感 | .482 | .000 | .000 |
| A7我觉得现在的工作是我生命中的一部分 | .473 | .000 | .000 |
| A6即便遇到阻碍，我仍然会选择继续现在的工作 | .502 | .000 | .000 |
| A5自我介绍时，我首先想起我的职业 | .427 | .000 | .000 |
| A4我愿意为现在的工作而作出一些牺牲 | .405 | .000 | .000 |
| A3我对我目前的职业感到非常满足 | .408 | .000 | .000 |
| A2与其它工作相比，我还是更喜欢现在的工作 | .393 | .000 | .000 |
| A1我热爱我现在的工作 | .411 | .000 | .000 |

## Standardized Indirect Effects - Upper Bounds (PC) (Group number 1 - Default model)

|  | Character\_strength | Career\_calling | Job\_performance |
| --- | --- | --- | --- |
| Career\_calling | .000 | .000 | .000 |
| Job\_performance | .380 | .000 | .000 |
| Professionalidentity | .659 | .434 | .000 |
| Personalgrowth | .696 | .450 | .000 |
| Workcontribution | .665 | .423 | .000 |
| Interpersonalpromotion | .665 | .418 | .000 |
| Medicalserviceperformance | .646 | .408 | .000 |
| B12同事和病人都认为我性格沉稳 | .000 | .000 | .000 |
| B13当他人遇到困难时，我愿意伸出援手 | .000 | .000 | .000 |
| B14我公平地对待所有人，不管他们是什么身份 | .000 | .000 | .000 |
| B15我总能乐观地发现它积极的一面 | .000 | .000 | .000 |
| A12我目前的职业可以为我带来快乐 | .603 | .000 | .000 |
| A11如果放弃现在的工作，我会觉得失去了意义 | .559 | .000 | .000 |
| A10我会经常思考工作相关的事 | .599 | .000 | .000 |
| A9我的职业一直存在于我的内心深处 | .650 | .000 | .000 |
| A8我的职业让我充满使命感 | .653 | .000 | .000 |
| A7我觉得现在的工作是我生命中的一部分 | .640 | .000 | .000 |
| A6即便遇到阻碍，我仍然会选择继续现在的工作 | .674 | .000 | .000 |
| A5自我介绍时，我首先想起我的职业 | .592 | .000 | .000 |
| A4我愿意为现在的工作而作出一些牺牲 | .582 | .000 | .000 |
| A3我对我目前的职业感到非常满足 | .566 | .000 | .000 |
| A2与其它工作相比，我还是更喜欢现在的工作 | .562 | .000 | .000 |
| A1我热爱我现在的工作 | .580 | .000 | .000 |

## Standardized Indirect Effects - Two Tailed Significance (PC) (Group number 1 - Default model)

|  | Character\_strength | Career\_calling | Job\_performance |
| --- | --- | --- | --- |
| Career\_calling | ... | ... | ... |
| Job\_performance | .000 | ... | ... |
| Professionalidentity | .000 | .000 | ... |
| Personalgrowth | .000 | .000 | ... |
| Workcontribution | .000 | .000 | ... |
| Interpersonalpromotion | .000 | .000 | ... |
| Medicalserviceperformance | .000 | .000 | ... |
| B12同事和病人都认为我性格沉稳 | ... | ... | ... |
| B13当他人遇到困难时，我愿意伸出援手 | ... | ... | ... |
| B14我公平地对待所有人，不管他们是什么身份 | ... | ... | ... |
| B15我总能乐观地发现它积极的一面 | ... | ... | ... |
| A12我目前的职业可以为我带来快乐 | .000 | ... | ... |
| A11如果放弃现在的工作，我会觉得失去了意义 | .000 | ... | ... |
| A10我会经常思考工作相关的事 | .000 | ... | ... |
| A9我的职业一直存在于我的内心深处 | .000 | ... | ... |
| A8我的职业让我充满使命感 | .000 | ... | ... |
| A7我觉得现在的工作是我生命中的一部分 | .000 | ... | ... |
| A6即便遇到阻碍，我仍然会选择继续现在的工作 | .000 | ... | ... |
| A5自我介绍时，我首先想起我的职业 | .000 | ... | ... |
| A4我愿意为现在的工作而作出一些牺牲 | .000 | ... | ... |
| A3我对我目前的职业感到非常满足 | .000 | ... | ... |
| A2与其它工作相比，我还是更喜欢现在的工作 | .000 | ... | ... |
| A1我热爱我现在的工作 | .000 | ... | ... |

## Bias-corrected percentile method (Group number 1 - Default model)

## 95% confidence intervals (bias-corrected percentile method)

## Scalar Estimates (Group number 1 - Default model)

## Regression Weights: (Group number 1 - Default model)

| Parameter | | | Estimate | Lower | Upper | P |
| --- | --- | --- | --- | --- | --- | --- |
| Career\_calling | <--- | Character\_strength | .801 | .667 | .968 | .000 |
| Job\_performance | <--- | Career\_calling | .265 | .176 | .369 | .000 |
| Job\_performance | <--- | Character\_strength | .382 | .256 | .533 | .000 |
| A6即便遇到阻碍，我仍然会选择继续现在的工作 | <--- | Career\_calling | 1.151 | 1.030 | 1.292 | .000 |
| A7我觉得现在的工作是我生命中的一部分 | <--- | Career\_calling | 1.047 | .927 | 1.198 | .000 |
| A8我的职业让我充满使命感 | <--- | Career\_calling | 1.020 | .920 | 1.137 | .000 |
| B15我总能乐观地发现它积极的一面 | <--- | Character\_strength | 1.000 | 1.000 | 1.000 | ... |
| B14我公平地对待所有人，不管他们是什么身份 | <--- | Character\_strength | 1.044 | .917 | 1.212 | .000 |
| B13当他人遇到困难时，我愿意伸出援手 | <--- | Character\_strength | 1.011 | .873 | 1.173 | .000 |
| Interpersonalpromotion | <--- | Job\_performance | .991 | .898 | 1.101 | .000 |
| Workcontribution | <--- | Job\_performance | 1.305 | 1.144 | 1.511 | .000 |
| Personalgrowth | <--- | Job\_performance | 1.257 | 1.123 | 1.422 | .000 |
| A11如果放弃现在的工作，我会觉得失去了意义 | <--- | Career\_calling | 1.107 | .952 | 1.289 | .000 |
| A3我对我目前的职业感到非常满足 | <--- | Career\_calling | 1.016 | .913 | 1.136 | .000 |
| A4我愿意为现在的工作而作出一些牺牲 | <--- | Career\_calling | .959 | .858 | 1.074 | .000 |
| A2与其它工作相比，我还是更喜欢现在的工作 | <--- | Career\_calling | .957 | .865 | 1.057 | .000 |
| A1我热爱我现在的工作 | <--- | Career\_calling | 1.000 | 1.000 | 1.000 | ... |
| A9我的职业一直存在于我的内心深处 | <--- | Career\_calling | 1.037 | .916 | 1.169 | .000 |
| A5自我介绍时，我首先想起我的职业 | <--- | Career\_calling | .963 | .821 | 1.110 | .000 |
| A12我目前的职业可以为我带来快乐 | <--- | Career\_calling | 1.163 | 1.025 | 1.323 | .000 |
| A10我会经常思考工作相关的事 | <--- | Career\_calling | .915 | .799 | 1.051 | .000 |
| B12同事和病人都认为我性格沉稳 | <--- | Character\_strength | .938 | .810 | 1.086 | .000 |
| Professionalidentity | <--- | Job\_performance | 1.354 | 1.182 | 1.588 | .000 |
| Medicalserviceperformance | <--- | Job\_performance | 1.000 | 1.000 | 1.000 | ... |

## Standardized Regression Weights: (Group number 1 - Default model)

| Parameter | | | Estimate | Lower | Upper | P |
| --- | --- | --- | --- | --- | --- | --- |
| Career\_calling | <--- | Character\_strength | .656 | .549 | .743 | .001 |
| Job\_performance | <--- | Career\_calling | .422 | .289 | .548 | .001 |
| Job\_performance | <--- | Character\_strength | .499 | .355 | .628 | .000 |
| A6即便遇到阻碍，我仍然会选择继续现在的工作 | <--- | Career\_calling | .904 | .877 | .928 | .000 |
| A7我觉得现在的工作是我生命中的一部分 | <--- | Career\_calling | .855 | .809 | .889 | .001 |
| A8我的职业让我充满使命感 | <--- | Career\_calling | .869 | .834 | .899 | .001 |
| B15我总能乐观地发现它积极的一面 | <--- | Character\_strength | .691 | .608 | .758 | .001 |
| B14我公平地对待所有人，不管他们是什么身份 | <--- | Character\_strength | .870 | .830 | .903 | .001 |
| B13当他人遇到困难时，我愿意伸出援手 | <--- | Character\_strength | .884 | .836 | .918 | .001 |
| Interpersonalpromotion | <--- | Job\_performance | .761 | .690 | .819 | .001 |
| Workcontribution | <--- | Job\_performance | .760 | .697 | .810 | .001 |
| Personalgrowth | <--- | Job\_performance | .809 | .759 | .851 | .000 |
| A11如果放弃现在的工作，我会觉得失去了意义 | <--- | Career\_calling | .735 | .668 | .791 | .001 |
| A3我对我目前的职业感到非常满足 | <--- | Career\_calling | .745 | .666 | .805 | .001 |
| A4我愿意为现在的工作而作出一些牺牲 | <--- | Career\_calling | .756 | .696 | .811 | .000 |
| A2与其它工作相比，我还是更喜欢现在的工作 | <--- | Career\_calling | .730 | .635 | .807 | .001 |
| A1我热爱我现在的工作 | <--- | Career\_calling | .755 | .667 | .816 | .001 |
| A9我的职业一直存在于我的内心深处 | <--- | Career\_calling | .865 | .821 | .898 | .000 |
| A5自我介绍时，我首先想起我的职业 | <--- | Career\_calling | .779 | .715 | .831 | .001 |
| A12我目前的职业可以为我带来快乐 | <--- | Career\_calling | .800 | .731 | .849 | .001 |
| A10我会经常思考工作相关的事 | <--- | Career\_calling | .778 | .709 | .838 | .000 |
| B12同事和病人都认为我性格沉稳 | <--- | Character\_strength | .691 | .613 | .757 | .000 |
| Professionalidentity | <--- | Job\_performance | .756 | .687 | .811 | .001 |
| Medicalserviceperformance | <--- | Job\_performance | .739 | .673 | .795 | .000 |

## Covariances: (Group number 1 - Default model)

| Parameter | | | Estimate | Lower | Upper | P |
| --- | --- | --- | --- | --- | --- | --- |
| e1 | <--> | e2 | .157 | .087 | .278 | .000 |
| e17 | <--> | e18 | .056 | .037 | .076 | .000 |
| e3 | <--> | e4 | .103 | .059 | .168 | .000 |
| e11 | <--> | e12 | .123 | .071 | .202 | .000 |
| e2 | <--> | e3 | .139 | .068 | .259 | .000 |
| e1 | <--> | e3 | .111 | .049 | .219 | .000 |
| e20 | <--> | e21 | .080 | .050 | .118 | .000 |
| e6 | <--> | e10 | -.044 | -.071 | -.020 | .000 |
| e2 | <--> | e8 | -.036 | -.063 | -.011 | .003 |
| e3 | <--> | e12 | .051 | .020 | .085 | .001 |
| e1 | <--> | e4 | .053 | .019 | .091 | .001 |
| e13 | <--> | e16 | .058 | .018 | .101 | .004 |

## Correlations: (Group number 1 - Default model)

| Parameter | | | Estimate | Lower | Upper | P |
| --- | --- | --- | --- | --- | --- | --- |
| e1 | <--> | e2 | .506 | .326 | .671 | .000 |
| e17 | <--> | e18 | .464 | .335 | .575 | .000 |
| e3 | <--> | e4 | .341 | .200 | .492 | .000 |
| e11 | <--> | e12 | .348 | .223 | .479 | .000 |
| e2 | <--> | e3 | .426 | .252 | .611 | .000 |
| e1 | <--> | e3 | .351 | .164 | .546 | .000 |
| e20 | <--> | e21 | .471 | .330 | .590 | .000 |
| e6 | <--> | e10 | -.274 | -.420 | -.125 | .000 |
| e2 | <--> | e8 | -.176 | -.295 | -.052 | .005 |
| e3 | <--> | e12 | .160 | .060 | .263 | .001 |
| e1 | <--> | e4 | .185 | .058 | .305 | .001 |
| e13 | <--> | e16 | .210 | .067 | .343 | .005 |

## Variances: (Group number 1 - Default model)

| Parameter | | | Estimate | Lower | Upper | P |
| --- | --- | --- | --- | --- | --- | --- |
| Character\_strength |  |  | .268 | .194 | .349 | .000 |
| e22 |  |  | .228 | .157 | .326 | .000 |
| e23 |  |  | .047 | .031 | .070 | .000 |
| e1 |  |  | .301 | .221 | .431 | .000 |
| e2 |  |  | .320 | .223 | .464 | .000 |
| e3 |  |  | .331 | .250 | .444 | .000 |
| e4 |  |  | .276 | .210 | .358 | .000 |
| e5 |  |  | .239 | .192 | .306 | .000 |
| e6 |  |  | .118 | .094 | .146 | .000 |
| e7 |  |  | .161 | .121 | .216 | .000 |
| e8 |  |  | .134 | .106 | .175 | .000 |
| e9 |  |  | .145 | .116 | .181 | .000 |
| e10 |  |  | .218 | .166 | .281 | .000 |
| e11 |  |  | .416 | .331 | .519 | .000 |
| e12 |  |  | .303 | .228 | .411 | .000 |
| e13 |  |  | .293 | .234 | .369 | .000 |
| e14 |  |  | .094 | .070 | .121 | .000 |
| e15 |  |  | .077 | .056 | .105 | .000 |
| e16 |  |  | .258 | .202 | .329 | .000 |
| e17 |  |  | .131 | .108 | .156 | .000 |
| e18 |  |  | .113 | .090 | .138 | .000 |
| e19 |  |  | .197 | .158 | .243 | .000 |
| e20 |  |  | .132 | .106 | .163 | .000 |
| e21 |  |  | .217 | .168 | .284 | .000 |

## Matrices (Group number 1 - Default model)

## Total Effects (Group number 1 - Default model)

## Total Effects - Lower Bounds (BC) (Group number 1 - Default model)

|  | Character\_strength | Career\_calling | Job\_performance |
| --- | --- | --- | --- |
| Career\_calling | .667 | .000 | .000 |
| Job\_performance | .477 | .176 | .000 |
| Professionalidentity | .654 | .224 | 1.182 |
| Personalgrowth | .611 | .217 | 1.123 |
| Workcontribution | .640 | .229 | 1.144 |
| Interpersonalpromotion | .475 | .175 | .898 |
| Medicalserviceperformance | .477 | .176 | 1.000 |
| B12同事和病人都认为我性格沉稳 | .810 | .000 | .000 |
| B13当他人遇到困难时，我愿意伸出援手 | .873 | .000 | .000 |
| B14我公平地对待所有人，不管他们是什么身份 | .917 | .000 | .000 |
| B15我总能乐观地发现它积极的一面 | 1.000 | .000 | .000 |
| A12我目前的职业可以为我带来快乐 | .795 | 1.025 | .000 |
| A11如果放弃现在的工作，我会觉得失去了意义 | .745 | .952 | .000 |
| A10我会经常思考工作相关的事 | .599 | .799 | .000 |
| A9我的职业一直存在于我的内心深处 | .699 | .916 | .000 |
| A8我的职业让我充满使命感 | .689 | .920 | .000 |
| A7我觉得现在的工作是我生命中的一部分 | .709 | .927 | .000 |
| A6即便遇到阻碍，我仍然会选择继续现在的工作 | .783 | 1.030 | .000 |
| A5自我介绍时，我首先想起我的职业 | .632 | .821 | .000 |
| A4我愿意为现在的工作而作出一些牺牲 | .629 | .858 | .000 |
| A3我对我目前的职业感到非常满足 | .683 | .913 | .000 |
| A2与其它工作相比，我还是更喜欢现在的工作 | .639 | .865 | .000 |
| A1我热爱我现在的工作 | .667 | 1.000 | .000 |

## Total Effects - Upper Bounds (BC) (Group number 1 - Default model)

|  | Character\_strength | Career\_calling | Job\_performance |
| --- | --- | --- | --- |
| Career\_calling | .968 | .000 | .000 |
| Job\_performance | .739 | .369 | .000 |
| Professionalidentity | .983 | .521 | 1.588 |
| Personalgrowth | .918 | .472 | 1.422 |
| Workcontribution | .932 | .496 | 1.511 |
| Interpersonalpromotion | .727 | .363 | 1.101 |
| Medicalserviceperformance | .739 | .369 | 1.000 |
| B12同事和病人都认为我性格沉稳 | 1.086 | .000 | .000 |
| B13当他人遇到困难时，我愿意伸出援手 | 1.173 | .000 | .000 |
| B14我公平地对待所有人，不管他们是什么身份 | 1.212 | .000 | .000 |
| B15我总能乐观地发现它积极的一面 | 1.000 | .000 | .000 |
| A12我目前的职业可以为我带来快乐 | 1.088 | 1.323 | .000 |
| A11如果放弃现在的工作，我会觉得失去了意义 | 1.049 | 1.289 | .000 |
| A10我会经常思考工作相关的事 | .881 | 1.051 | .000 |
| A9我的职业一直存在于我的内心深处 | .988 | 1.169 | .000 |
| A8我的职业让我充满使命感 | .967 | 1.137 | .000 |
| A7我觉得现在的工作是我生命中的一部分 | .987 | 1.198 | .000 |
| A6即便遇到阻碍，我仍然会选择继续现在的工作 | 1.078 | 1.292 | .000 |
| A5自我介绍时，我首先想起我的职业 | .926 | 1.110 | .000 |
| A4我愿意为现在的工作而作出一些牺牲 | .933 | 1.074 | .000 |
| A3我对我目前的职业感到非常满足 | .974 | 1.136 | .000 |
| A2与其它工作相比，我还是更喜欢现在的工作 | .916 | 1.057 | .000 |
| A1我热爱我现在的工作 | .968 | 1.000 | .000 |

## Total Effects - Two Tailed Significance (BC) (Group number 1 - Default model)

|  | Character\_strength | Career\_calling | Job\_performance |
| --- | --- | --- | --- |
| Career\_calling | .000 | ... | ... |
| Job\_performance | .000 | .000 | ... |
| Professionalidentity | .000 | .000 | .000 |
| Personalgrowth | .000 | .000 | .000 |
| Workcontribution | .000 | .000 | .000 |
| Interpersonalpromotion | .000 | .001 | .000 |
| Medicalserviceperformance | .000 | .000 | ... |
| B12同事和病人都认为我性格沉稳 | .000 | ... | ... |
| B13当他人遇到困难时，我愿意伸出援手 | .000 | ... | ... |
| B14我公平地对待所有人，不管他们是什么身份 | .000 | ... | ... |
| B15我总能乐观地发现它积极的一面 | ... | ... | ... |
| A12我目前的职业可以为我带来快乐 | .000 | .000 | ... |
| A11如果放弃现在的工作，我会觉得失去了意义 | .000 | .000 | ... |
| A10我会经常思考工作相关的事 | .000 | .000 | ... |
| A9我的职业一直存在于我的内心深处 | .000 | .000 | ... |
| A8我的职业让我充满使命感 | .000 | .000 | ... |
| A7我觉得现在的工作是我生命中的一部分 | .000 | .000 | ... |
| A6即便遇到阻碍，我仍然会选择继续现在的工作 | .000 | .000 | ... |
| A5自我介绍时，我首先想起我的职业 | .000 | .000 | ... |
| A4我愿意为现在的工作而作出一些牺牲 | .000 | .000 | ... |
| A3我对我目前的职业感到非常满足 | .000 | .000 | ... |
| A2与其它工作相比，我还是更喜欢现在的工作 | .000 | .000 | ... |
| A1我热爱我现在的工作 | .000 | ... | ... |

## Standardized Total Effects (Group number 1 - Default model)

## Standardized Total Effects - Lower Bounds (BC) (Group number 1 - Default model)

|  | Character\_strength | Career\_calling | Job\_performance |
| --- | --- | --- | --- |
| Career\_calling | .549 | .000 | .000 |
| Job\_performance | .696 | .289 | .000 |
| Professionalidentity | .508 | .210 | .687 |
| Personalgrowth | .551 | .232 | .759 |
| Workcontribution | .508 | .220 | .697 |
| Interpersonalpromotion | .513 | .225 | .690 |
| Medicalserviceperformance | .496 | .220 | .673 |
| B12同事和病人都认为我性格沉稳 | .613 | .000 | .000 |
| B13当他人遇到困难时，我愿意伸出援手 | .836 | .000 | .000 |
| B14我公平地对待所有人，不管他们是什么身份 | .830 | .000 | .000 |
| B15我总能乐观地发现它积极的一面 | .608 | .000 | .000 |
| A12我目前的职业可以为我带来快乐 | .441 | .731 | .000 |
| A11如果放弃现在的工作，我会觉得失去了意义 | .401 | .668 | .000 |
| A10我会经常思考工作相关的事 | .413 | .709 | .000 |
| A9我的职业一直存在于我的内心深处 | .477 | .821 | .000 |
| A8我的职业让我充满使命感 | .478 | .834 | .000 |
| A7我觉得现在的工作是我生命中的一部分 | .473 | .809 | .000 |
| A6即便遇到阻碍，我仍然会选择继续现在的工作 | .499 | .877 | .000 |
| A5自我介绍时，我首先想起我的职业 | .426 | .715 | .000 |
| A4我愿意为现在的工作而作出一些牺牲 | .404 | .696 | .000 |
| A3我对我目前的职业感到非常满足 | .409 | .666 | .000 |
| A2与其它工作相比，我还是更喜欢现在的工作 | .393 | .635 | .000 |
| A1我热爱我现在的工作 | .410 | .667 | .000 |

## Standardized Total Effects - Upper Bounds (BC) (Group number 1 - Default model)

|  | Character\_strength | Career\_calling | Job\_performance |
| --- | --- | --- | --- |
| Career\_calling | .743 | .000 | .000 |
| Job\_performance | .846 | .548 | .000 |
| Professionalidentity | .656 | .431 | .811 |
| Personalgrowth | .694 | .447 | .851 |
| Workcontribution | .663 | .417 | .810 |
| Interpersonalpromotion | .665 | .416 | .819 |
| Medicalserviceperformance | .646 | .407 | .795 |
| B12同事和病人都认为我性格沉稳 | .757 | .000 | .000 |
| B13当他人遇到困难时，我愿意伸出援手 | .918 | .000 | .000 |
| B14我公平地对待所有人，不管他们是什么身份 | .903 | .000 | .000 |
| B15我总能乐观地发现它积极的一面 | .758 | .000 | .000 |
| A12我目前的职业可以为我带来快乐 | .603 | .849 | .000 |
| A11如果放弃现在的工作，我会觉得失去了意义 | .558 | .791 | .000 |
| A10我会经常思考工作相关的事 | .598 | .838 | .000 |
| A9我的职业一直存在于我的内心深处 | .648 | .898 | .000 |
| A8我的职业让我充满使命感 | .650 | .899 | .000 |
| A7我觉得现在的工作是我生命中的一部分 | .639 | .889 | .000 |
| A6即便遇到阻碍，我仍然会选择继续现在的工作 | .673 | .928 | .000 |
| A5自我介绍时，我首先想起我的职业 | .592 | .831 | .000 |
| A4我愿意为现在的工作而作出一些牺牲 | .581 | .811 | .000 |
| A3我对我目前的职业感到非常满足 | .567 | .805 | .000 |
| A2与其它工作相比，我还是更喜欢现在的工作 | .562 | .807 | .000 |
| A1我热爱我现在的工作 | .579 | .816 | .000 |

## Standardized Total Effects - Two Tailed Significance (BC) (Group number 1 - Default model)

|  | Character\_strength | Career\_calling | Job\_performance |
| --- | --- | --- | --- |
| Career\_calling | .001 | ... | ... |
| Job\_performance | .000 | .001 | ... |
| Professionalidentity | .001 | .001 | .001 |
| Personalgrowth | .001 | .000 | .000 |
| Workcontribution | .000 | .001 | .001 |
| Interpersonalpromotion | .000 | .000 | .001 |
| Medicalserviceperformance | .000 | .000 | .000 |
| B12同事和病人都认为我性格沉稳 | .000 | ... | ... |
| B13当他人遇到困难时，我愿意伸出援手 | .001 | ... | ... |
| B14我公平地对待所有人，不管他们是什么身份 | .001 | ... | ... |
| B15我总能乐观地发现它积极的一面 | .001 | ... | ... |
| A12我目前的职业可以为我带来快乐 | .000 | .001 | ... |
| A11如果放弃现在的工作，我会觉得失去了意义 | .000 | .001 | ... |
| A10我会经常思考工作相关的事 | .000 | .000 | ... |
| A9我的职业一直存在于我的内心深处 | .000 | .000 | ... |
| A8我的职业让我充满使命感 | .001 | .001 | ... |
| A7我觉得现在的工作是我生命中的一部分 | .000 | .001 | ... |
| A6即便遇到阻碍，我仍然会选择继续现在的工作 | .001 | .000 | ... |
| A5自我介绍时，我首先想起我的职业 | .000 | .001 | ... |
| A4我愿意为现在的工作而作出一些牺牲 | .000 | .000 | ... |
| A3我对我目前的职业感到非常满足 | .000 | .001 | ... |
| A2与其它工作相比，我还是更喜欢现在的工作 | .000 | .001 | ... |
| A1我热爱我现在的工作 | .000 | .001 | ... |

## Direct Effects (Group number 1 - Default model)

## Direct Effects - Lower Bounds (BC) (Group number 1 - Default model)

|  | Character\_strength | Career\_calling | Job\_performance |
| --- | --- | --- | --- |
| Career\_calling | .667 | .000 | .000 |
| Job\_performance | .256 | .176 | .000 |
| Professionalidentity | .000 | .000 | 1.182 |
| Personalgrowth | .000 | .000 | 1.123 |
| Workcontribution | .000 | .000 | 1.144 |
| Interpersonalpromotion | .000 | .000 | .898 |
| Medicalserviceperformance | .000 | .000 | 1.000 |
| B12同事和病人都认为我性格沉稳 | .810 | .000 | .000 |
| B13当他人遇到困难时，我愿意伸出援手 | .873 | .000 | .000 |
| B14我公平地对待所有人，不管他们是什么身份 | .917 | .000 | .000 |
| B15我总能乐观地发现它积极的一面 | 1.000 | .000 | .000 |
| A12我目前的职业可以为我带来快乐 | .000 | 1.025 | .000 |
| A11如果放弃现在的工作，我会觉得失去了意义 | .000 | .952 | .000 |
| A10我会经常思考工作相关的事 | .000 | .799 | .000 |
| A9我的职业一直存在于我的内心深处 | .000 | .916 | .000 |
| A8我的职业让我充满使命感 | .000 | .920 | .000 |
| A7我觉得现在的工作是我生命中的一部分 | .000 | .927 | .000 |
| A6即便遇到阻碍，我仍然会选择继续现在的工作 | .000 | 1.030 | .000 |
| A5自我介绍时，我首先想起我的职业 | .000 | .821 | .000 |
| A4我愿意为现在的工作而作出一些牺牲 | .000 | .858 | .000 |
| A3我对我目前的职业感到非常满足 | .000 | .913 | .000 |
| A2与其它工作相比，我还是更喜欢现在的工作 | .000 | .865 | .000 |
| A1我热爱我现在的工作 | .000 | 1.000 | .000 |

## Direct Effects - Upper Bounds (BC) (Group number 1 - Default model)

|  | Character\_strength | Career\_calling | Job\_performance |
| --- | --- | --- | --- |
| Career\_calling | .968 | .000 | .000 |
| Job\_performance | .533 | .369 | .000 |
| Professionalidentity | .000 | .000 | 1.588 |
| Personalgrowth | .000 | .000 | 1.422 |
| Workcontribution | .000 | .000 | 1.511 |
| Interpersonalpromotion | .000 | .000 | 1.101 |
| Medicalserviceperformance | .000 | .000 | 1.000 |
| B12同事和病人都认为我性格沉稳 | 1.086 | .000 | .000 |
| B13当他人遇到困难时，我愿意伸出援手 | 1.173 | .000 | .000 |
| B14我公平地对待所有人，不管他们是什么身份 | 1.212 | .000 | .000 |
| B15我总能乐观地发现它积极的一面 | 1.000 | .000 | .000 |
| A12我目前的职业可以为我带来快乐 | .000 | 1.323 | .000 |
| A11如果放弃现在的工作，我会觉得失去了意义 | .000 | 1.289 | .000 |
| A10我会经常思考工作相关的事 | .000 | 1.051 | .000 |
| A9我的职业一直存在于我的内心深处 | .000 | 1.169 | .000 |
| A8我的职业让我充满使命感 | .000 | 1.137 | .000 |
| A7我觉得现在的工作是我生命中的一部分 | .000 | 1.198 | .000 |
| A6即便遇到阻碍，我仍然会选择继续现在的工作 | .000 | 1.292 | .000 |
| A5自我介绍时，我首先想起我的职业 | .000 | 1.110 | .000 |
| A4我愿意为现在的工作而作出一些牺牲 | .000 | 1.074 | .000 |
| A3我对我目前的职业感到非常满足 | .000 | 1.136 | .000 |
| A2与其它工作相比，我还是更喜欢现在的工作 | .000 | 1.057 | .000 |
| A1我热爱我现在的工作 | .000 | 1.000 | .000 |

## Direct Effects - Two Tailed Significance (BC) (Group number 1 - Default model)

|  | Character\_strength | Career\_calling | Job\_performance |
| --- | --- | --- | --- |
| Career\_calling | .000 | ... | ... |
| Job\_performance | .000 | .000 | ... |
| Professionalidentity | ... | ... | .000 |
| Personalgrowth | ... | ... | .000 |
| Workcontribution | ... | ... | .000 |
| Interpersonalpromotion | ... | ... | .000 |
| Medicalserviceperformance | ... | ... | ... |
| B12同事和病人都认为我性格沉稳 | .000 | ... | ... |
| B13当他人遇到困难时，我愿意伸出援手 | .000 | ... | ... |
| B14我公平地对待所有人，不管他们是什么身份 | .000 | ... | ... |
| B15我总能乐观地发现它积极的一面 | ... | ... | ... |
| A12我目前的职业可以为我带来快乐 | ... | .000 | ... |
| A11如果放弃现在的工作，我会觉得失去了意义 | ... | .000 | ... |
| A10我会经常思考工作相关的事 | ... | .000 | ... |
| A9我的职业一直存在于我的内心深处 | ... | .000 | ... |
| A8我的职业让我充满使命感 | ... | .000 | ... |
| A7我觉得现在的工作是我生命中的一部分 | ... | .000 | ... |
| A6即便遇到阻碍，我仍然会选择继续现在的工作 | ... | .000 | ... |
| A5自我介绍时，我首先想起我的职业 | ... | .000 | ... |
| A4我愿意为现在的工作而作出一些牺牲 | ... | .000 | ... |
| A3我对我目前的职业感到非常满足 | ... | .000 | ... |
| A2与其它工作相比，我还是更喜欢现在的工作 | ... | .000 | ... |
| A1我热爱我现在的工作 | ... | ... | ... |

## Standardized Direct Effects (Group number 1 - Default model)

## Standardized Direct Effects - Lower Bounds (BC) (Group number 1 - Default model)

|  | Character\_strength | Career\_calling | Job\_performance |
| --- | --- | --- | --- |
| Career\_calling | .549 | .000 | .000 |
| Job\_performance | .355 | .289 | .000 |
| Professionalidentity | .000 | .000 | .687 |
| Personalgrowth | .000 | .000 | .759 |
| Workcontribution | .000 | .000 | .697 |
| Interpersonalpromotion | .000 | .000 | .690 |
| Medicalserviceperformance | .000 | .000 | .673 |
| B12同事和病人都认为我性格沉稳 | .613 | .000 | .000 |
| B13当他人遇到困难时，我愿意伸出援手 | .836 | .000 | .000 |
| B14我公平地对待所有人，不管他们是什么身份 | .830 | .000 | .000 |
| B15我总能乐观地发现它积极的一面 | .608 | .000 | .000 |
| A12我目前的职业可以为我带来快乐 | .000 | .731 | .000 |
| A11如果放弃现在的工作，我会觉得失去了意义 | .000 | .668 | .000 |
| A10我会经常思考工作相关的事 | .000 | .709 | .000 |
| A9我的职业一直存在于我的内心深处 | .000 | .821 | .000 |
| A8我的职业让我充满使命感 | .000 | .834 | .000 |
| A7我觉得现在的工作是我生命中的一部分 | .000 | .809 | .000 |
| A6即便遇到阻碍，我仍然会选择继续现在的工作 | .000 | .877 | .000 |
| A5自我介绍时，我首先想起我的职业 | .000 | .715 | .000 |
| A4我愿意为现在的工作而作出一些牺牲 | .000 | .696 | .000 |
| A3我对我目前的职业感到非常满足 | .000 | .666 | .000 |
| A2与其它工作相比，我还是更喜欢现在的工作 | .000 | .635 | .000 |
| A1我热爱我现在的工作 | .000 | .667 | .000 |

## Standardized Direct Effects - Upper Bounds (BC) (Group number 1 - Default model)

|  | Character\_strength | Career\_calling | Job\_performance |
| --- | --- | --- | --- |
| Career\_calling | .743 | .000 | .000 |
| Job\_performance | .628 | .548 | .000 |
| Professionalidentity | .000 | .000 | .811 |
| Personalgrowth | .000 | .000 | .851 |
| Workcontribution | .000 | .000 | .810 |
| Interpersonalpromotion | .000 | .000 | .819 |
| Medicalserviceperformance | .000 | .000 | .795 |
| B12同事和病人都认为我性格沉稳 | .757 | .000 | .000 |
| B13当他人遇到困难时，我愿意伸出援手 | .918 | .000 | .000 |
| B14我公平地对待所有人，不管他们是什么身份 | .903 | .000 | .000 |
| B15我总能乐观地发现它积极的一面 | .758 | .000 | .000 |
| A12我目前的职业可以为我带来快乐 | .000 | .849 | .000 |
| A11如果放弃现在的工作，我会觉得失去了意义 | .000 | .791 | .000 |
| A10我会经常思考工作相关的事 | .000 | .838 | .000 |
| A9我的职业一直存在于我的内心深处 | .000 | .898 | .000 |
| A8我的职业让我充满使命感 | .000 | .899 | .000 |
| A7我觉得现在的工作是我生命中的一部分 | .000 | .889 | .000 |
| A6即便遇到阻碍，我仍然会选择继续现在的工作 | .000 | .928 | .000 |
| A5自我介绍时，我首先想起我的职业 | .000 | .831 | .000 |
| A4我愿意为现在的工作而作出一些牺牲 | .000 | .811 | .000 |
| A3我对我目前的职业感到非常满足 | .000 | .805 | .000 |
| A2与其它工作相比，我还是更喜欢现在的工作 | .000 | .807 | .000 |
| A1我热爱我现在的工作 | .000 | .816 | .000 |

## Standardized Direct Effects - Two Tailed Significance (BC) (Group number 1 - Default model)

|  | Character\_strength | Career\_calling | Job\_performance |
| --- | --- | --- | --- |
| Career\_calling | .001 | ... | ... |
| Job\_performance | .000 | .001 | ... |
| Professionalidentity | ... | ... | .001 |
| Personalgrowth | ... | ... | .000 |
| Workcontribution | ... | ... | .001 |
| Interpersonalpromotion | ... | ... | .001 |
| Medicalserviceperformance | ... | ... | .000 |
| B12同事和病人都认为我性格沉稳 | .000 | ... | ... |
| B13当他人遇到困难时，我愿意伸出援手 | .001 | ... | ... |
| B14我公平地对待所有人，不管他们是什么身份 | .001 | ... | ... |
| B15我总能乐观地发现它积极的一面 | .001 | ... | ... |
| A12我目前的职业可以为我带来快乐 | ... | .001 | ... |
| A11如果放弃现在的工作，我会觉得失去了意义 | ... | .001 | ... |
| A10我会经常思考工作相关的事 | ... | .000 | ... |
| A9我的职业一直存在于我的内心深处 | ... | .000 | ... |
| A8我的职业让我充满使命感 | ... | .001 | ... |
| A7我觉得现在的工作是我生命中的一部分 | ... | .001 | ... |
| A6即便遇到阻碍，我仍然会选择继续现在的工作 | ... | .000 | ... |
| A5自我介绍时，我首先想起我的职业 | ... | .001 | ... |
| A4我愿意为现在的工作而作出一些牺牲 | ... | .000 | ... |
| A3我对我目前的职业感到非常满足 | ... | .001 | ... |
| A2与其它工作相比，我还是更喜欢现在的工作 | ... | .001 | ... |
| A1我热爱我现在的工作 | ... | .001 | ... |

## Indirect Effects (Group number 1 - Default model)

## Indirect Effects - Lower Bounds (BC) (Group number 1 - Default model)

|  | Character\_strength | Career\_calling | Job\_performance |
| --- | --- | --- | --- |
| Career\_calling | .000 | .000 | .000 |
| Job\_performance | .146 | .000 | .000 |
| Professionalidentity | .654 | .224 | .000 |
| Personalgrowth | .611 | .217 | .000 |
| Workcontribution | .640 | .229 | .000 |
| Interpersonalpromotion | .475 | .175 | .000 |
| Medicalserviceperformance | .477 | .176 | .000 |
| B12同事和病人都认为我性格沉稳 | .000 | .000 | .000 |
| B13当他人遇到困难时，我愿意伸出援手 | .000 | .000 | .000 |
| B14我公平地对待所有人，不管他们是什么身份 | .000 | .000 | .000 |
| B15我总能乐观地发现它积极的一面 | .000 | .000 | .000 |
| A12我目前的职业可以为我带来快乐 | .795 | .000 | .000 |
| A11如果放弃现在的工作，我会觉得失去了意义 | .745 | .000 | .000 |
| A10我会经常思考工作相关的事 | .599 | .000 | .000 |
| A9我的职业一直存在于我的内心深处 | .699 | .000 | .000 |
| A8我的职业让我充满使命感 | .689 | .000 | .000 |
| A7我觉得现在的工作是我生命中的一部分 | .709 | .000 | .000 |
| A6即便遇到阻碍，我仍然会选择继续现在的工作 | .783 | .000 | .000 |
| A5自我介绍时，我首先想起我的职业 | .632 | .000 | .000 |
| A4我愿意为现在的工作而作出一些牺牲 | .629 | .000 | .000 |
| A3我对我目前的职业感到非常满足 | .683 | .000 | .000 |
| A2与其它工作相比，我还是更喜欢现在的工作 | .639 | .000 | .000 |
| A1我热爱我现在的工作 | .667 | .000 | .000 |

## Indirect Effects - Upper Bounds (BC) (Group number 1 - Default model)

|  | Character\_strength | Career\_calling | Job\_performance |
| --- | --- | --- | --- |
| Career\_calling | .000 | .000 | .000 |
| Job\_performance | .299 | .000 | .000 |
| Professionalidentity | .983 | .521 | .000 |
| Personalgrowth | .918 | .472 | .000 |
| Workcontribution | .932 | .496 | .000 |
| Interpersonalpromotion | .727 | .363 | .000 |
| Medicalserviceperformance | .739 | .369 | .000 |
| B12同事和病人都认为我性格沉稳 | .000 | .000 | .000 |
| B13当他人遇到困难时，我愿意伸出援手 | .000 | .000 | .000 |
| B14我公平地对待所有人，不管他们是什么身份 | .000 | .000 | .000 |
| B15我总能乐观地发现它积极的一面 | .000 | .000 | .000 |
| A12我目前的职业可以为我带来快乐 | 1.088 | .000 | .000 |
| A11如果放弃现在的工作，我会觉得失去了意义 | 1.049 | .000 | .000 |
| A10我会经常思考工作相关的事 | .881 | .000 | .000 |
| A9我的职业一直存在于我的内心深处 | .988 | .000 | .000 |
| A8我的职业让我充满使命感 | .967 | .000 | .000 |
| A7我觉得现在的工作是我生命中的一部分 | .987 | .000 | .000 |
| A6即便遇到阻碍，我仍然会选择继续现在的工作 | 1.078 | .000 | .000 |
| A5自我介绍时，我首先想起我的职业 | .926 | .000 | .000 |
| A4我愿意为现在的工作而作出一些牺牲 | .933 | .000 | .000 |
| A3我对我目前的职业感到非常满足 | .974 | .000 | .000 |
| A2与其它工作相比，我还是更喜欢现在的工作 | .916 | .000 | .000 |
| A1我热爱我现在的工作 | .968 | .000 | .000 |

## Indirect Effects - Two Tailed Significance (BC) (Group number 1 - Default model)

|  | Character\_strength | Career\_calling | Job\_performance |
| --- | --- | --- | --- |
| Career\_calling | ... | ... | ... |
| Job\_performance | .000 | ... | ... |
| Professionalidentity | .000 | .000 | ... |
| Personalgrowth | .000 | .000 | ... |
| Workcontribution | .000 | .000 | ... |
| Interpersonalpromotion | .000 | .001 | ... |
| Medicalserviceperformance | .000 | .000 | ... |
| B12同事和病人都认为我性格沉稳 | ... | ... | ... |
| B13当他人遇到困难时，我愿意伸出援手 | ... | ... | ... |
| B14我公平地对待所有人，不管他们是什么身份 | ... | ... | ... |
| B15我总能乐观地发现它积极的一面 | ... | ... | ... |
| A12我目前的职业可以为我带来快乐 | .000 | ... | ... |
| A11如果放弃现在的工作，我会觉得失去了意义 | .000 | ... | ... |
| A10我会经常思考工作相关的事 | .000 | ... | ... |
| A9我的职业一直存在于我的内心深处 | .000 | ... | ... |
| A8我的职业让我充满使命感 | .000 | ... | ... |
| A7我觉得现在的工作是我生命中的一部分 | .000 | ... | ... |
| A6即便遇到阻碍，我仍然会选择继续现在的工作 | .000 | ... | ... |
| A5自我介绍时，我首先想起我的职业 | .000 | ... | ... |
| A4我愿意为现在的工作而作出一些牺牲 | .000 | ... | ... |
| A3我对我目前的职业感到非常满足 | .000 | ... | ... |
| A2与其它工作相比，我还是更喜欢现在的工作 | .000 | ... | ... |
| A1我热爱我现在的工作 | .000 | ... | ... |

## Standardized Indirect Effects (Group number 1 - Default model)

## Standardized Indirect Effects - Lower Bounds (BC) (Group number 1 - Default model)

|  | Character\_strength | Career\_calling | Job\_performance |
| --- | --- | --- | --- |
| Career\_calling | .000 | .000 | .000 |
| Job\_performance | .190 | .000 | .000 |
| Professionalidentity | .508 | .210 | .000 |
| Personalgrowth | .551 | .232 | .000 |
| Workcontribution | .508 | .220 | .000 |
| Interpersonalpromotion | .513 | .225 | .000 |
| Medicalserviceperformance | .496 | .220 | .000 |
| B12同事和病人都认为我性格沉稳 | .000 | .000 | .000 |
| B13当他人遇到困难时，我愿意伸出援手 | .000 | .000 | .000 |
| B14我公平地对待所有人，不管他们是什么身份 | .000 | .000 | .000 |
| B15我总能乐观地发现它积极的一面 | .000 | .000 | .000 |
| A12我目前的职业可以为我带来快乐 | .441 | .000 | .000 |
| A11如果放弃现在的工作，我会觉得失去了意义 | .401 | .000 | .000 |
| A10我会经常思考工作相关的事 | .413 | .000 | .000 |
| A9我的职业一直存在于我的内心深处 | .477 | .000 | .000 |
| A8我的职业让我充满使命感 | .478 | .000 | .000 |
| A7我觉得现在的工作是我生命中的一部分 | .473 | .000 | .000 |
| A6即便遇到阻碍，我仍然会选择继续现在的工作 | .499 | .000 | .000 |
| A5自我介绍时，我首先想起我的职业 | .426 | .000 | .000 |
| A4我愿意为现在的工作而作出一些牺牲 | .404 | .000 | .000 |
| A3我对我目前的职业感到非常满足 | .409 | .000 | .000 |
| A2与其它工作相比，我还是更喜欢现在的工作 | .393 | .000 | .000 |
| A1我热爱我现在的工作 | .410 | .000 | .000 |

## Standardized Indirect Effects - Upper Bounds (BC) (Group number 1 - Default model)

|  | Character\_strength | Career\_calling | Job\_performance |
| --- | --- | --- | --- |
| Career\_calling | .000 | .000 | .000 |
| Job\_performance | .380 | .000 | .000 |
| Professionalidentity | .656 | .431 | .000 |
| Personalgrowth | .694 | .447 | .000 |
| Workcontribution | .663 | .417 | .000 |
| Interpersonalpromotion | .665 | .416 | .000 |
| Medicalserviceperformance | .646 | .407 | .000 |
| B12同事和病人都认为我性格沉稳 | .000 | .000 | .000 |
| B13当他人遇到困难时，我愿意伸出援手 | .000 | .000 | .000 |
| B14我公平地对待所有人，不管他们是什么身份 | .000 | .000 | .000 |
| B15我总能乐观地发现它积极的一面 | .000 | .000 | .000 |
| A12我目前的职业可以为我带来快乐 | .603 | .000 | .000 |
| A11如果放弃现在的工作，我会觉得失去了意义 | .558 | .000 | .000 |
| A10我会经常思考工作相关的事 | .598 | .000 | .000 |
| A9我的职业一直存在于我的内心深处 | .648 | .000 | .000 |
| A8我的职业让我充满使命感 | .650 | .000 | .000 |
| A7我觉得现在的工作是我生命中的一部分 | .639 | .000 | .000 |
| A6即便遇到阻碍，我仍然会选择继续现在的工作 | .673 | .000 | .000 |
| A5自我介绍时，我首先想起我的职业 | .592 | .000 | .000 |
| A4我愿意为现在的工作而作出一些牺牲 | .581 | .000 | .000 |
| A3我对我目前的职业感到非常满足 | .567 | .000 | .000 |
| A2与其它工作相比，我还是更喜欢现在的工作 | .562 | .000 | .000 |
| A1我热爱我现在的工作 | .579 | .000 | .000 |

## Standardized Indirect Effects - Two Tailed Significance (BC) (Group number 1 - Default model)

|  | Character\_strength | Career\_calling | Job\_performance |
| --- | --- | --- | --- |
| Career\_calling | ... | ... | ... |
| Job\_performance | .000 | ... | ... |
| Professionalidentity | .001 | .001 | ... |
| Personalgrowth | .001 | .000 | ... |
| Workcontribution | .000 | .001 | ... |
| Interpersonalpromotion | .000 | .000 | ... |
| Medicalserviceperformance | .000 | .000 | ... |
| B12同事和病人都认为我性格沉稳 | ... | ... | ... |
| B13当他人遇到困难时，我愿意伸出援手 | ... | ... | ... |
| B14我公平地对待所有人，不管他们是什么身份 | ... | ... | ... |
| B15我总能乐观地发现它积极的一面 | ... | ... | ... |
| A12我目前的职业可以为我带来快乐 | .000 | ... | ... |
| A11如果放弃现在的工作，我会觉得失去了意义 | .000 | ... | ... |
| A10我会经常思考工作相关的事 | .000 | ... | ... |
| A9我的职业一直存在于我的内心深处 | .000 | ... | ... |
| A8我的职业让我充满使命感 | .001 | ... | ... |
| A7我觉得现在的工作是我生命中的一部分 | .000 | ... | ... |
| A6即便遇到阻碍，我仍然会选择继续现在的工作 | .001 | ... | ... |
| A5自我介绍时，我首先想起我的职业 | .000 | ... | ... |
| A4我愿意为现在的工作而作出一些牺牲 | .000 | ... | ... |
| A3我对我目前的职业感到非常满足 | .000 | ... | ... |
| A2与其它工作相比，我还是更喜欢现在的工作 | .000 | ... | ... |
| A1我热爱我现在的工作 | .000 | ... | ... |

## Minimization History (Default model)

| Iteration |  | Negative eigenvalues | Condition # | Smallest eigenvalue | Diameter | F | NTries | Ratio |
| --- | --- | --- | --- | --- | --- | --- | --- | --- |
| 0 | e | 16 |  | -2.502 | 9999.000 | 7235.423 | 0 | 9999.000 |
| 1 | e | 19 |  | -.561 | 1.859 | 4704.908 | 18 | .414 |
| 2 | e\* | 14 |  | -.359 | 1.111 | 3062.036 | 4 | .984 |
| 3 | e | 7 |  | -.266 | .483 | 2534.272 | 5 | .784 |
| 4 | e\* | 2 |  | -.154 | .785 | 1668.432 | 5 | .901 |
| 5 | e | 0 | 3570.112 |  | .695 | 1173.434 | 5 | .868 |
| 6 | e | 0 | 454.391 |  | .816 | 934.733 | 5 | .000 |
| 7 | e | 0 | 171.550 |  | .775 | 735.895 | 2 | .000 |
| 8 | e | 0 | 191.844 |  | .568 | 642.215 | 1 | 1.170 |
| 9 | e | 0 | 251.158 |  | .311 | 628.491 | 1 | 1.130 |
| 10 | e | 0 | 284.763 |  | .102 | 627.682 | 1 | 1.053 |
| 11 | e | 0 | 282.496 |  | .011 | 627.675 | 1 | 1.007 |
| 12 | e | 0 | 292.946 |  | .000 | 627.675 | 1 | 1.000 |

## Bootstrap (Default model)

## Summary of Bootstrap Iterations (Default model)

## (Default model)

| Iterations | Method 0 | Method 1 | Method 2 |
| --- | --- | --- | --- |
| 1 | 0 | 0 | 0 |
| 2 | 0 | 0 | 0 |
| 3 | 0 | 0 | 0 |
| 4 | 0 | 0 | 0 |
| 5 | 0 | 0 | 0 |
| 6 | 0 | 0 | 0 |
| 7 | 0 | 5 | 0 |
| 8 | 0 | 166 | 0 |
| 9 | 0 | 959 | 0 |
| 10 | 0 | 1432 | 0 |
| 11 | 0 | 1389 | 0 |
| 12 | 0 | 602 | 0 |
| 13 | 0 | 330 | 0 |
| 14 | 0 | 86 | 0 |
| 15 | 0 | 26 | 0 |
| 16 | 0 | 4 | 0 |
| 17 | 0 | 1 | 0 |
| 18 | 0 | 0 | 0 |
| 19 | 0 | 0 | 0 |
| Total | 0 | 5000 | 0 |

0 bootstrap samples were unused because of a singular covariance matrix.

0 bootstrap samples were unused because a solution was not found.

5000 usable bootstrap samples were obtained.

## Bootstrap Distributions (Default model)

## ML discrepancy (implied vs sample) (Default model)

|  |  |  |
| --- | --- | --- |
|  |  | |-------------------- |
|  | 590.984 | |\* |
|  | 653.948 | |\* |
|  | 716.912 | |\*\*\*\*\* |
|  | 779.877 | |\*\*\*\*\*\*\*\*\*\*\*\*\*\* |
|  | 842.841 | |\*\*\*\*\*\*\*\*\*\*\*\*\*\*\*\*\*\*\*\* |
|  | 905.805 | |\*\*\*\*\*\*\*\*\*\*\*\*\*\*\*\*\*\*\*\* |
|  | 968.770 | |\*\*\*\*\*\*\*\*\*\*\*\*\*\*\* |
| N = 5000 | 1031.734 | |\*\*\*\*\*\*\*\*\* |
| Mean = 901.885 | 1094.698 | |\*\*\*\* |
| S. e. = 1.576 | 1157.663 | |\*\* |
|  | 1220.627 | |\* |
|  | 1283.591 | |\* |
|  | 1346.556 | |\* |
|  | 1409.520 | |\* |
|  | 1472.485 | |\* |
|  |  | |-------------------- |

## ML discrepancy (implied vs pop) (Default model)

|  |  |  |
| --- | --- | --- |
|  |  | |-------------------- |
|  | 681.434 | |\* |
|  | 707.370 | |\*\*\*\*\* |
|  | 733.305 | |\*\*\*\*\*\*\*\*\*\*\*\*\*\*\*\* |
|  | 759.241 | |\*\*\*\*\*\*\*\*\*\*\*\*\*\*\*\*\*\*\*\* |
|  | 785.177 | |\*\*\*\*\*\*\*\*\*\*\*\*\*\*\*\* |
|  | 811.112 | |\*\*\*\*\*\*\*\*\*\* |
|  | 837.048 | |\*\*\*\*\* |
| N = 5000 | 862.984 | |\*\* |
| Mean = 774.390 | 888.919 | |\*\* |
| S. e. = .600 | 914.855 | |\* |
|  | 940.791 | |\* |
|  | 966.726 | |\* |
|  | 992.662 | |\* |
|  | 1018.598 | | |
|  | 1044.534 | |\* |
|  |  | |-------------------- |

## K-L overoptimism (unstabilized) (Default model)

|  |  |  |
| --- | --- | --- |
|  |  | |-------------------- |
|  | -1255.176 | |\* |
|  | -1016.575 | |\* |
|  | -777.974 | |\* |
|  | -539.373 | |\*\*\*\* |
|  | -300.772 | |\*\*\*\*\*\*\*\*\*\* |
|  | -62.170 | |\*\*\*\*\*\*\*\*\*\*\*\*\*\*\* |
|  | 176.431 | |\*\*\*\*\*\*\*\*\*\*\*\*\*\*\*\*\*\*\*\* |
| N = 5000 | 415.032 | |\*\*\*\*\*\*\*\*\*\*\*\*\*\*\*\*\*\* |
| Mean = 280.586 | 653.633 | |\*\*\*\*\*\*\*\*\*\*\*\*\*\* |
| S. e. = 6.302 | 892.235 | |\*\*\*\*\*\*\*\* |
|  | 1130.836 | |\*\*\*\* |
|  | 1369.437 | |\*\* |
|  | 1608.038 | |\* |
|  | 1846.640 | |\* |
|  | 2085.241 | |\* |
|  |  | |-------------------- |

## K-L overoptimism (stabilized) (Default model)

|  |  |  |
| --- | --- | --- |
|  |  | |-------------------- |
|  | -102.547 | |\* |
|  | -29.942 | |\* |
|  | 42.664 | |\*\*\* |
|  | 115.269 | |\*\*\*\*\*\*\*\*\*\* |
|  | 187.874 | |\*\*\*\*\*\*\*\*\*\*\*\*\*\*\*\*\* |
|  | 260.480 | |\*\*\*\*\*\*\*\*\*\*\*\*\*\*\*\*\*\*\*\* |
|  | 333.085 | |\*\*\*\*\*\*\*\*\*\*\*\*\*\*\*\* |
| N = 5000 | 405.690 | |\*\*\*\*\*\*\*\*\*\*\* |
| Mean = 286.283 | 478.296 | |\*\*\*\*\*\*\* |
| S. e. = 1.865 | 550.901 | |\*\*\* |
|  | 623.506 | |\*\* |
|  | 696.112 | |\* |
|  | 768.717 | |\* |
|  | 841.322 | |\* |
|  | 913.928 | |\* |
|  |  | |-------------------- |

## Model Fit Summary

## CMIN

| Model | NPAR | CMIN | DF | P | CMIN/DF |
| --- | --- | --- | --- | --- | --- |
| Default model | 57 | 627.675 | 174 | .000 | 3.607 |
| Saturated model | 231 | .000 | 0 |
| Independence model | 21 | 7645.318 | 210 | .000 | 36.406 |

## RMR, GFI

| Model | RMR | GFI | AGFI | PGFI |
| --- | --- | --- | --- | --- |
| Default model | .031 | .865 | .820 | .651 |
| Saturated model | .000 | 1.000 |  |  |
| Independence model | .299 | .146 | .061 | .133 |

## Baseline Comparisons

| Model | NFI Delta1 | RFI rho1 | IFI Delta2 | TLI rho2 | CFI |
| --- | --- | --- | --- | --- | --- |
| Default model | .918 | .901 | .939 | .926 | .939 |
| Saturated model | 1.000 |  | 1.000 |  | 1.000 |
| Independence model | .000 | .000 | .000 | .000 | .000 |

## Parsimony-Adjusted Measures

| Model | PRATIO | PNFI | PCFI |
| --- | --- | --- | --- |
| Default model | .829 | .761 | .778 |
| Saturated model | .000 | .000 | .000 |
| Independence model | 1.000 | .000 | .000 |

## NCP

| Model | NCP | LO 90 | HI 90 |
| --- | --- | --- | --- |
| Default model | 453.675 | 380.933 | 533.997 |
| Saturated model | .000 | .000 | .000 |
| Independence model | 7435.318 | 7152.835 | 7724.138 |

## FMIN

| Model | FMIN | F0 | LO 90 | HI 90 |
| --- | --- | --- | --- | --- |
| Default model | 1.520 | 1.098 | .922 | 1.293 |
| Saturated model | .000 | .000 | .000 | .000 |
| Independence model | 18.512 | 18.003 | 17.319 | 18.703 |

## RMSEA

| Model | RMSEA | LO 90 | HI 90 | PCLOSE |
| --- | --- | --- | --- | --- |
| Default model | .079 | .073 | .086 | .000 |
| Independence model | .293 | .287 | .298 | .000 |

## AIC

| Model | AIC | BCC | BIC | CAIC |
| --- | --- | --- | --- | --- |
| Default model | 741.675 | 748.090 | 971.150 | 1028.150 |
| Saturated model | 462.000 | 487.995 | 1391.975 | 1622.975 |
| Independence model | 7687.318 | 7689.681 | 7771.861 | 7792.861 |

## ECVI

| Model | ECVI | LO 90 | HI 90 | MECVI |
| --- | --- | --- | --- | --- |
| Default model | 1.796 | 1.620 | 1.990 | 1.811 |
| Saturated model | 1.119 | 1.119 | 1.119 | 1.182 |
| Independence model | 18.613 | 17.929 | 19.313 | 18.619 |

## HOELTER

| Model | HOELTER .05 | HOELTER .01 |
| --- | --- | --- |
| Default model | 136 | 145 |
| Independence model | 14 | 15 |

## Execution time summary

|  |  |
| --- | --- |
| Minimization: | .031 |
| Miscellaneous: | .396 |
| Bootstrap: | 4.256 |
| Total: | 4.683 |
