# Supplementary material for: The impact of medical staff’s character strengths on job performance in Hangzhou hospitals
Source: Front Psychol. 2023 Nov 23;14:1291851. doi: 10.3389/fpsyg.2023.1291851 (PMC10701392; doi:10.3389/fpsyg.2023.1291851)
Supplement: Supplementary file 1 [file Data_Sheet_1.ZIP › statistical analysis process/Dummy variable coding.docx]

|  | 1 | 2 |  |
| --- | --- | --- | --- |
| 性别 | 男（参照） | 女 |  |
| 女 | 0 | 1 | 01 |
| 男 |  |  | 00 |

|  | 1 | 2 | 3 | 4 |  |
| --- | --- | --- | --- | --- | --- |
| 年龄 | 29 岁及以下（参照） | 30~39 岁 | 40~49 岁 | 50 岁及以上 |  |
| 30~39 岁 | 0 | 1 | 0 | 0 | 0100 |
| 40~49 岁 | 0 | 0 | 1 | 0 | 0010 |
| 50 岁及以上 | 0 | 0 | 0 | 1 | 0001 |
| 29 岁及以下 |  |  |  |  | 0000 |

|  | 1 | 2 | 3 |  |
| --- | --- | --- | --- | --- |
| 职业岗位 | 临床医生（参照） | 护士 | 医技人员 |  |
| 护士 | 0 | 1 | 0 | 010 |
| 医技人员 | 0 | 0 | 1 | 001 |
| 临床医生 | 0 | 0 | 0 | 000 |

|  | 1 | 2 | 3 | 4 |  |
| --- | --- | --- | --- | --- | --- |
| 学历 | 大专及以下（参照） | 本科 | 硕士研究生 | 博士研究生 |  |
| 本科 | 0 | 1 | 0 | 0 | 0100 |
| 硕士研究生 | 0 | 0 | 1 | 0 | 0010 |
| 博士研究生 | 0 | 0 | 0 | 1 | 0001 |
| 大专及以下 | 0 | 0 | 0 | 0 | 0000 |

|  | 1 | 2 | 3 | 4 | 5 |  |
| --- | --- | --- | --- | --- | --- | --- |
| 从业时间 | 5 年及以下（参照） | 6~10 年 | 11~15 年 | 16~20 年 | 21年及以上 |  |
| 6~10 年 | 0 | 1 | 0 | 0 | 0 | 01000 |
| 11~15 年 | 0 | 0 | 1 | 0 | 0 | 00100 |
| 16~20 年 | 0 | 0 | 0 | 1 | 0 | 00010 |
| 21年及以上 | 0 | 0 | 0 | 0 | 1 | 00001 |
| 5 年及以下 | 0 | 0 | 0 | 0 | 0 | 00000 |

|  | 1 | 2 | 3 | 4 | 5 |  |
| --- | --- | --- | --- | --- | --- | --- |
| 职称 | 无职称（参照） | 初级 | 中级 | 副高级 | 正高级 |  |
| 初级 | 0 | 1 | 0 | 0 | 0 | 01000 |
| 中级 | 0 | 0 | 1 | 0 | 0 | 00100 |
| 副高级 | 0 | 0 | 0 | 1 | 0 | 00010 |
| 正高级 | 0 | 0 | 0 | 0 | 1 | 00001 |
| 无职称 | 0 | 0 | 0 | 0 | 0 | 00000 |

|  | 1 | 2 |  |
| --- | --- | --- | --- |
| 编制情况 | 有编制（参照） | 合同聘用 |  |
| 合同聘用 | 0 | 1 | 01 |
| 有编制 | 0 | 0 | 00 |

|  | 1 | 2 | 3 |  |
| --- | --- | --- | --- | --- |
| 职务 | 无职务（参照） | 治疗/责任组长 | 科室负责人 |  |
| 治疗/责任组长 | 0 | 1 | 0 | 0100 |
| 科室负责人 | 0 | 0 | 1 | 0010 |
| 无职务 | 0 | 0 | 0 | 0000 |

|  | 1 | 2 | 3 | 4 | 5 | 6 | 7 | 8 |  |
| --- | --- | --- | --- | --- | --- | --- | --- | --- | --- |
| 科室 | 内科（参照） | 外科 | 急诊科 | 儿科 | 妇产科 | 重症监护室 | 医技科室（检验、放射等） | 其他 |  |
| 外科 | 0 | 1 | 0 | 0 | 0 | 0 | 0 | 0 | 01000000 |
| 急诊科 | 0 | 0 | 1 | 0 | 0 | 0 | 0 | 0 | 00100000 |
| 儿科 | 0 | 0 | 0 | 1 | 0 | 0 | 0 | 0 | 00010000 |
| 妇产科 | 0 | 0 | 0 | 0 | 1 | 0 | 0 | 0 | 00001000 |
| 重症监护室 | 0 | 0 | 0 | 0 | 0 | 1 | 0 | 0 | 00000100 |
| 医技科室 | 0 | 0 | 0 | 0 | 0 | 0 | 1 | 0 | 00000010 |
| 其他 | 0 | 0 | 0 | 0 | 0 | 0 | 0 | 1 | 00000001 |
| 内科 | 0 | 0 | 0 | 0 | 0 | 0 | 0 | 0 | 00000000 |

|  | 1 | 2 | 3 |  |
| --- | --- | --- | --- | --- |
| 医院级别 | 三级医院（参照） | 二级医院 | 社区卫生服务中心 |  |
| 二级医院 | 0 | 1 | 0 | 010 |
| 社区卫生服务中心 | 0 | 0 | 1 | 001 |
| 三级医院 | 0 | 0 | 0 | 000 |
