# Supplementary material for: The impact of medical staff’s character strengths on job performance in Hangzhou hospitals
Source: Front Psychol. 2023 Nov 23;14:1291851. doi: 10.3389/fpsyg.2023.1291851 (PMC10701392; doi:10.3389/fpsyg.2023.1291851)
Supplement: Supplementary file 2 [file Data_Sheet_2.docx]

**Supplementary Information**

**Title:** The Impact of Medical Staff’s Character Strengths on Job Performance in Hangzhou Hospitals

**Journal name:***Frontiers in Psychology*

Authors:

Xin Zhou^1,2^, Yinrui Zhang^3^,Yuhang Wang^4^, Haixia Wang^5^, Shuaijun Sun^6^ ,Yongjian Xu^1^, Xianhong Huang^5*^

Author affiliations:

1. School of Public Policy and Administration,Xi'an Jiaotong University,Xi'an, China

2.Affiliated Hospital of Hangzhou Normal University, Hangzhou, China

3.The First Affiliated Hospital Zhejiang University School of Medicine liangzhu Branch,Hangzhou, China

4.Tianlai Primary School, Qibin District, Hebi, China

5.Department of Health Policy and Management, School of Public Health, Hangzhou Normal University, Hangzhou, China

6.Jiangsu Key Laboratory for Pharmacology and Safety Evaluation of Chinese Materia Medica, School of Pharmacy, Nanjing University of Chinese Medicine, Nanjing, Jiangsu, P.R. China

*Corresponding authors:
Xianhong Huang (email: hxh974291@163.com; Tel.:13588126044)

**Supplementary Table1**

| **RELIABILITY OF QUWSTIONNAIRE** | | | | |
| --- | --- | --- | --- | --- |
| **Dimension** | **item** | **Corrected Item-Total Correlation** | **Cronbach's Alpha if Item Deleted** | **Cronbach's α factor** |
| Career calling | A1 | 0.776 | 0.952 | 0.955 |
|  | A2 | 0.761 | 0.952 |  |
|  | A3 | 0.78 | 0.951 |  |
|  | A4 | 0.774 | 0.952 |  |
|  | A5 | 0.735 | 0.953 |  |
|  | A6 | 0.865 | 0.949 |  |
|  | A7 | 0.82 | 0.95 |  |
|  | A8 | 0.829 | 0.95 |  |
|  | A9 | 0.813 | 0.951 |  |
|  | A10 | 0.728 | 0.953 |  |
|  | A11 | 0.721 | 0.954 |  |
|  | A12 | 0.809 | 0.951 |  |
| Character strength | B12 | 0.675 | 0.84 | 0.863 |
|  | B13 | 0.77 | 0.806 |  |
|  | B14 | 0.751 | 0.81 |  |
|  | B15 | 0.674 | 0.845 |  |
| Job performance | E1 | 0.733 | 0.95 | 0.953 |
|  | E2 | 0.755 | 0.95 |  |
|  | E3 | 0.746 | 0.95 |  |
|  | E4 | 0.74 | 0.95 |  |
|  | E5 | 0.751 | 0.95 |  |
|  | E6 | 0.754 | 0.95 |  |
|  | E7 | 0.754 | 0.95 |  |
|  | E8 | 0.758 | 0.95 |  |
|  | E9 | 0.696 | 0.951 |  |
|  | E10 | 0.666 | 0.951 |  |
|  | E11 | 0.805 | 0.948 |  |
|  | E12 | 0.798 | 0.948 |  |
|  | E13 | 0.78 | 0.949 |  |
|  | E14 | 0.78 | 0.949 |  |
|  | E15 | 0.757 | 0.949 |  |
|  | E16 | 0.599 | 0.955 |  |

**Supplementary Table2**

| **Rotated Component Matrix** | | | | | | |
| --- | --- | --- | --- | --- | --- | --- |
| **item** | **1** | **2** | **3** | **4** | **5** | **Extraction** |
| A1 | 0.782 |  |  |  |  | 0.733 |
| A2 | 0.792 |  |  |  |  | 0.694 |
| A3 | 0.762 |  |  |  |  | 0.717 |
| A4 | 0.731 |  |  |  |  | 0.671 |
| A5 | 0.71 |  |  |  |  | 0.624 |
| A6 | 0.808 |  |  |  |  | 0.805 |
| A7 | 0.769 |  |  |  |  | 0.747 |
| A8 | 0.8 |  |  |  |  | 0.763 |
| A9 | 0.763 |  |  |  |  | 0.738 |
| A10 | 0.633 |  |  |  |  | 0.64 |
| A11 | 0.613 |  |  |  |  | 0.686 |
| A12 | 0.728 |  |  |  |  | 0.746 |
| B12 |  |  |  | 0.604 |  | 0.593 |
| B13 |  |  |  | 0.759 |  | 0.836 |
| B14 |  |  |  | 0.766 |  | 0.81 |
| B15 |  |  |  | 0.663 |  | 0.757 |
| E1 |  | 0.777 |  |  |  | 0.716 |
| E2 |  | 0.828 |  |  |  | 0.795 |
| E3 |  | 0.784 |  |  |  | 0.756 |
| E4 |  | 0.769 |  |  |  | 0.715 |
| E5 |  | 0.792 |  |  |  | 0.76 |
| E6 |  | 0.767 |  |  |  | 0.733 |
| E7 |  | 0.814 |  |  |  | 0.79 |
| E8 |  | 0.764 |  |  |  | 0.733 |
| E9 |  |  |  |  | 0.661 | 0.781 |
| E10 |  |  |  |  | 0.636 | 0.756 |
| E11 |  |  | 0.723 |  |  | 0.831 |
| E12 |  |  | 0.765 |  |  | 0.849 |
| E13 |  |  | 0.734 |  |  | 0.835 |
| E14 |  |  | 0.731 |  |  | 0.819 |
| E15 |  |  | 0.734 |  |  | 0.786 |
| E16 |  |  | 0.64 |  |  | 0.768 |
| **Rotation Sums of Squared Loadings Cumulative %** | 74.944 | | | | | |

**Supplementary Table3**

| **Linear regression model of job performance** | | | |
| --- | --- | --- | --- |
| **Variable** | **Model 1** | **Model 2** | **Model 3** |
| (Constant) | 4.165 | 1.497 | 1.155 |
| Gender |  |  |  |
| Male（reference group） |  |  |  |
| Female | 0.05 | 0.053 | 0.056 |
| Age |  |  |  |
| 29 years old and below |  |  |  |
| 30-39 | 0.119 | 0.115 | 0.078 |
| 40-49 | 0.003 | 0.05 | 0.001 |
| 50 years old and above | -0.061 | 0.023 | -0.107 |
| Professional post |  |  |  |
| Clinician（reference group） |  |  |  |
| Nurse | 0.015 | 0.051 | 0.073 |
| Medical and technical staff | -0.037 | 0.063 | 0.054 |
| Academic degree |  |  |  |
| College degree or below（reference group） |  |  |  |
| Undergraduate course | 0.05 | 0.27 | 0.232 |
| Master | 0.105 | 0.338 | 0.291 |
| PhD | 0.325 | 0.512* | 0.399* |
| Working time |  |  |  |
| 5 years and below（reference group） |  |  |  |
| 6-10 years | 0.055 | -0.052 | -0.038 |
| 11-15 years | -0.027 | -0.108 | -0.079 |
| 16-20 years | 0.139 | 0.024 | 0.061 |
| 21 years and above | 0.122 | -0.056 | 0.029 |
| Professional title |  |  |  |
| No professional title（reference group） |  |  |  |
| Primary | -0.052 | -0.058 | -0.032 |
| Middle | -0.215* | -0.152 | -0.113 |
| Deputy senior | -0.194 | -0.155 | -0.1 |
| Positive advanced | -0.239 | -0.029 | 0.047 |
| Authorized strength |  |  |  |
| Have a system（reference group） |  |  |  |
| Contract employment | -0.099 | -0.06 | -0.027 |
| Position |  |  |  |
| No position（reference group） |  |  |  |
| Treatment/Responsibility Team Leader | 0.104 | -0.018 | 0.002 |
| Department leader | 0.353* | 0.152 | 0.072 |
| Departments |  |  |  |
| Internal medicine（reference group） |  |  |  |
| Surgery department | 0.11 | 0.09 | 0.109* |
| Emergency department | -0.061 | -0.122 | -0.095 |
| Pediatrics | 0.112 | 0.157 | 0.137 |
| Gynecology and obstetrics | 0.292** | 0.119 | 0.112 |
| Intensive care unit | -0.11 | -0.075 | -0.101 |
| Medical department (inspection, radiation, etc.) | 0.139 | -0.033 | -0.016 |
| Others | 0.058 | 0.023 | 0.069 |
| Hospital level |  |  |  |
| Tertiary hospital（reference group） |  |  |  |
| Secondary hospital | -0.209 | -0.159 | -0.081 |
| community health service center | -0.585*** | -0.23 | -0.18 |
| Character strength |  | 0.576*** | 0.351*** |
| Career calling |  |  | 0.316*** |
| R Square | 0.147 | 0.509 | 0.614 |
| ΔR Square | 0.147 | 0.362 | 0.105 |
| F | 2.283*** | 13.215*** | 19.602*** |

*P<0.05,**P<0.01,***P<0.001

**Supplementary Table4**

Explanation of variables in a Figure 2:

| Career calling | | A1 I love my current job. |
| --- | --- | --- |
|  |  | A2 Compared to other jobs, I still prefer my current job. |
|  |  | A3 I feel extremely satisfied with my current profession. |
|  |  | A4 I am willing to make some sacrifices for my current job. |
|  |  | A5 When introducing myself, my profession is the first thing that comes to mind. |
|  |  | A6 Even in the face of obstacles, I would still choose to continue my current job. |
|  |  | A7 I feel that my current job is a part of my life. |
|  |  | A8 My profession fills me with a sense of purpose. |
|  |  | A9 My profession has always existed deep within my heart. |
|  |  | A10 Even when I'm not doing my current job, I often find myself thinking about work-related matters. |
|  |  | A11 If I were to give up my current job, I would feel a sense of loss of purpose. |
|  |  | A12 My current profession brings me joy. |
| Character strength | | B12 Both my colleagues and patients perceive me as having a calm personality. |
|  |  | B13 I am willing to lend a helping hand when others encounter difficulties. |
|  |  | B14 I treat everyone fairly, regardless of their status or identity. |
|  |  | B15 When others see the negative side of things, I always optimistically find the positive aspect. |
| Job performance | Medical service performance | E1 I always adhere to the standard procedures and protocols of medical service when carrying out my work. |
|  |  | E2 I consistently deliver high-quality medical services. |
|  |  | E3 I always meet the requirements of hospital performance assessments. |
|  |  | E4 I consistently adhere to the core quality and safety regulations related to medical services. |
|  | Interpersonal promotion | E5 I have a harmonious relationship with my colleagues. |
|  |  | E6 I frequently provide assistance to my colleagues in their work. |
|  |  | E7 I have good collaboration with my colleagues. |
|  |  | E8 I always communicate patiently with patients during the process of providing medical services. |
|  | Work contribution | E9 I proactively seek opportunities to take on challenging tasks. |
|  |  | E10 I often take initiative to undertake responsibilities beyond my role. |
|  | Personal growth | E11 I continuously learn and acquire new knowledge and skills in my work. |
|  |  | E12 I feel that I am constantly improving and making progress in my work. |
|  |  | E13 I am constantly growing and developing in my work. |
|  | Professional identity | E14 The work I am currently engaged in gives me a sense of purpose and value. |
|  |  | E15 When I experience professional burnout, I proactively seek ways to overcome it. |
|  |  | E16 If given the chance to choose again, I would still choose this job. |
